# BUB1 Is Identified as a Potential Therapeutic Target for Pancreatic Cancer Treatment

**DOI:** 10.3389/fpubh.2022.900853

**Published:** 2022-06-13

**Authors:** Ming Li, Xiaoyang Duan, Yajie Xiao, Meng Yuan, Zhikun Zhao, Xiaoli Cui, Dongfang Wu, Jian Shi

**Affiliations:** ^1^Department of General Surgery, Shijiazhuang People's Hospital, Shijiazhuang, China; ^2^Department of Medical Oncology, The Fourth Hospital of Hebei Medical University, Hebei Tumor Hospital, Shijiazhuang, China; ^3^Translational Medicine, YuceBio Technology Co., Ltd., Shenzhen, China; ^4^Internal Medical, University of Occupational and Environmental Health, Fukuoka, Japan

**Keywords:** pancreatic cancer, single-cell data, dendritic cells, ligand–receptor interactions, CD74, Bub1, therapeutic targets, bioinformatics analysis

## Abstract

Pancreatic cancer is one of the most challenging cancer types in clinical treatment worldwide. This study aimed to understand the tumorigenesis mechanism and explore potential therapeutic targets for patients with pancreatic cancer. Single-cell data and expression profiles of pancreatic cancer samples and normal tissues from multiple databases were included. Comprehensive bioinformatics analyses were applied to clarify tumor microenvironment and identify key genes involved in cancer development. Immense difference of cell types was shown between tumor and normal samples. Four cell types (B cell_1, B cell_2, cancer cell_3, and CD1C+_B dendritic cell_3) were screened to be significantly associated with prognosis. Three ligand–receptor pairs, including CD74-MIF, CD74-COPA, and CD74-APP, greatly contributed to tumorigenesis. High expression of BUB1 (BUB1 Mitotic Checkpoint Serine/Threonine Kinase) was closely correlated with worse prognosis. CD1C+_B dendritic cell_3 played a key role in tumorigenesis and cancer progression possibly through CD74-MIF. BUB1 can serve as a prognostic biomarker and a therapeutic target for patients with pancreatic cancer. The study provided a novel insight into studying the molecular mechanism of pancreatic cancer development and proposed a potential strategy for exploiting new drugs.

## Introduction

In 2020, 495,773 new cases of pancreatic cancer were diagnosed all over the world, showing an increase of more than 1/3 cases compared with the data in 2015 (1, 2). High-income countries, especially Europe and Northern America, have a high incidence of pancreatic cancer, which was partially attributed to the improved diagnosis and an aging population (1). Smoking is the most studied risk factor and has been demonstrated to be highly associated with pancreatic cancer (3). Evidence showed that smokers have two- to three-fold risk much higher than non-smokers, and an obvious dosage–risk relationship is observed (3). Other risk factors, such as dietary factors (4), low physical activity (5), and obesity (6), are also associated with pancreatic cancer.

Pancreatic cancer is one of the most lethal cancers with high mortality, ranking the seventh in cancer-related mortality. Patients with pancreatic cancer were already in an advanced stage when diagnosed, which increases difficulty in treatment. In addition, pancreatic cancer is not sensitive to conventional therapies such as chemotherapy, radiotherapy, or molecular-targeted therapy. Surgery is still the main strategy for non-metastatic or local advanced patients. The 5-year survival rate is about 15–25% of patients accepting surgery compared with <7% of an averaged 5-year survival in all individuals (7). However, not more than 20% of patients with pancreatic cancer could be treated by surgery, whereas over 60% of surgery-managed patients will undergo relapse in the first year (8). Therefore, effective biomarkers for early screening pancreatic cancer or molecular targets for personalized therapy are urgently needed.

CA19-9 is a well-known biomarker of pancreatic cancer with a sensitivity of about 80% for diagnosis. It can also serve as a monitor to evaluate the response systematic treatment in the neoadjuvant or surgery (9). However, CA19-9 level may be affected by other diseases such as biliary obstruction (10). Other biomarkers related to transforming growth factor-beta (TGF-β), angiogenesis, inflammation, and immune response have been explored (11). For example, a meta-analysis demonstrated that loss of SMAD4 (SMAD Family Member 4) expression was associated with worse prognosis of patients with pancreatic cancer (12). Although a number of biomarkers for predicting prognosis have been developed, the mechanisms and treatments of pancreatic cancer should be improved. Molecular-targeted therapy is promising strategy for managing pancreatic cancer.

**Figure 1:**
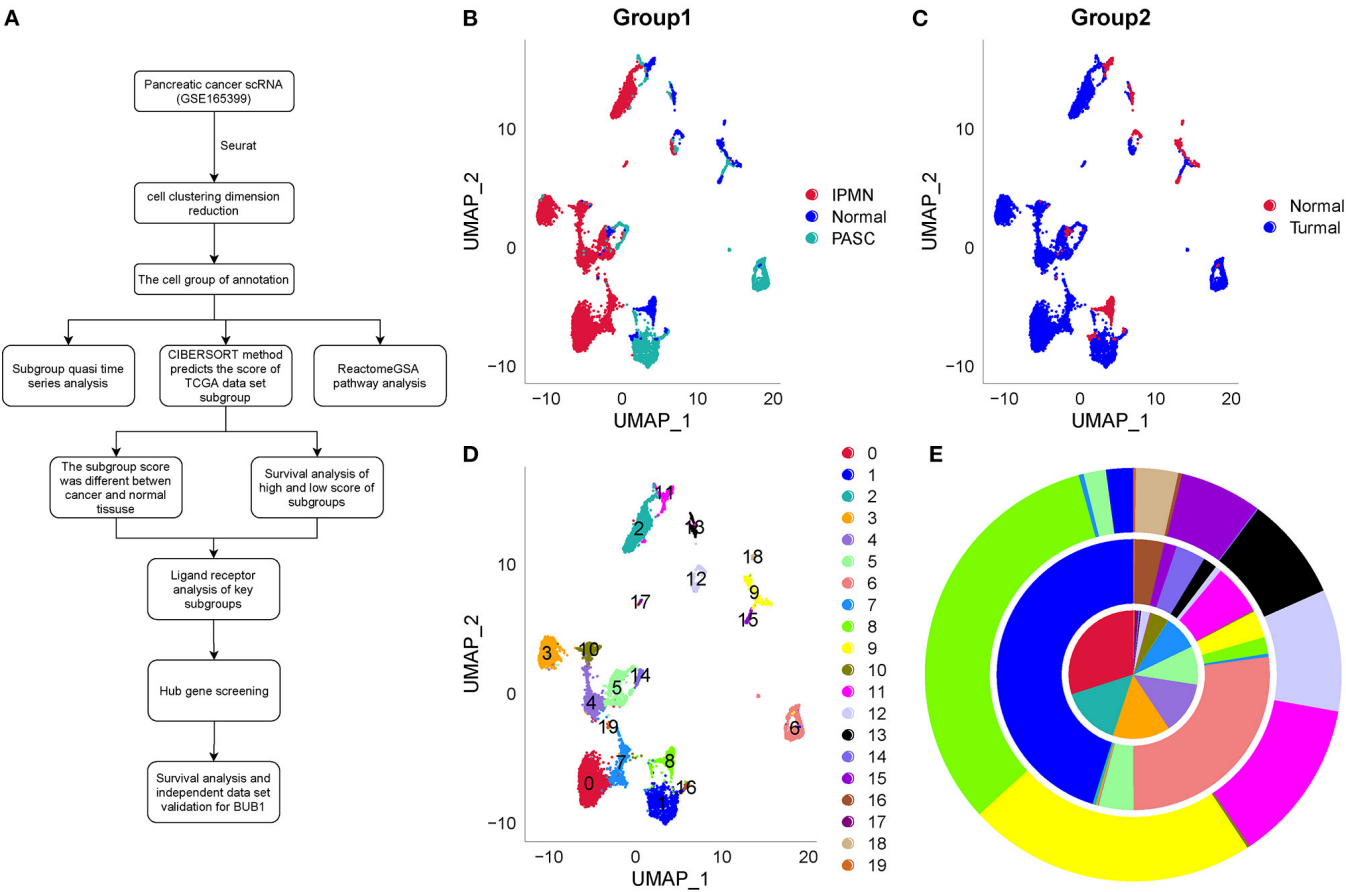
Work flowchart and clustering and dimensionality reduction of one normal sample and two tumor samples by UMAP. **(A)** Work flowchart. **(B)** The distribution of three samples labeled by three colors after dimensionality reduction. **(C)** The distribution of one normal sample and tumor samples. **(D)** 20 subgroups from C0 to C19 of three samples. **(E)** The proportion of different subgroups in each sample. IPMN, PASC, and the normal sample located from inside to outside, respectively. IPMN, intraductal papillary mucinous neoplasm; PASC, pancreatic adenosquamous carcinoma.

The *BUB1* gene is located on human chromosome 2q14. Its encoded protein is a platform protein for spindle physical examination. It is the basis for other cell components to be accurately located in the spindle. *BUB1* gene plays an indispensable role in maintaining correct chromosome separation and reducing aneuploid formation during mitosis. Increasing studies have shown that *BUB1* plays an important role in the occurrence and development of tumors. Qi et al. (13) identified the expression of *BUB1* in liver hepatocellular carcinoma (LIHC) tissue by immunohistochemistry. BUB1 expression is accompanied by immune cell infiltration into LIHC tissue. Yun et al. (14) found that *BUB1* is a key gene in colorectal cancer. Gao et al. (15) observed that *BUB1* can be used as a prognostic marker of gastric cancer. Alam et al. (16) found that BUB1 may be used as a biomarker in breast cancer. Jiang et al. (17) reported that BUB1 mediates STAT3 signaling pathway to drive the occurrence and development of bladder cancer. These studies have shown that *BUB1* is an important gene in cell cycle control and DNA damage repair, but it is rarely reported in pancreatic cancer. The development of single-cell sequencing technology allows an in-depth understanding of the occurrence and development mechanism of pancreatic cancer, the heterogeneity of tumor microenvironment, and the formation mechanism of drug resistance, so as to find new therapeutic targets. Earlier, Zhao et al. (18) identified the metabolic reprogramming of human colon immune cells in different locations and disease states by using single-cell RNA sequencing (scRNA-seq). Lai et al. (19) constructed a prognostic model for predicting the survival rate of human glioblastoma by comprehensive analysis of scRNA-seq dataset and RNA-seq dataset. Wang et al. (20) identified the heterogeneity of CD8^+^ T cells and novel biomarker genes in hepatocellular carcinoma by single-cell sequencing. These studies have shown that it is effective to use single-cell technology to find tumor heterogeneity and key biomarkers. In the recent years, single-cell technology has been greatly improved and applied in studying mechanisms from different aspects in pancreatic cancer (21–23). Single-cell data provide an expanded space for exploring tumor microenvironment, heterogeneity, and other aspects in cancer. Therefore, in this study, we applied the scRNA-seq data of pancreatic cancer to distinguish differential cell types between normal and tumor samples. Workflow is as shown in Figure 1A. Together with other independent data, we discovered hub genes highly associated with prognosis. The study provides a new insight into understanding the mechanisms of pancreatic cancer development.

## Materials and Methods

### Data Source

The scRNA-seq data (GSE165399) (24) of an intraductal papillary mucinous neoplasm (IPMN) sample, a pancreatic adenosquamous carcinoma (PASC) sample, and a normal sample were downloaded from Gene Expression Omnibus (GEO) database (https://www.ncbi.nlm.nih.gov/geo/). The Cancer Genome Atlas (TCGA) dataset, including 177 tumor samples, was downloaded from TCGA database (https://portal.gdc.cancer.gov/). Other normal samples of pancreatic tissue were downloaded from UCSC Xena database (http://xena.ucsc.edu/). Other pancreatic adenocarcinoma (PAAD) samples, including GSE21501 (25), GSE28735 (26), GSE57495 (27), GSE62452 (28), and GSE85916, were downloaded from GEO database. The clinical characteristics of each dataset are shown in Table 1.

**Table 1:** Clinical characteristics of each dataset.

	**TCGA**	**GSE21501**	**GSE28735**	**GSE57495**	**GSE62452**	**GSE85916**
**Event**
Alive	85	36	13	21	16	23
Dead	100	66	29	42	50	57
**T.Stage**
T1	7	2	NA	NA	NA	NA
T2	24	16	NA	NA	NA	NA
T3	148	79	NA	NA	NA	NA
T4	4	1	NA	NA	NA	NA
Unknown	2	0	42	63	66	80
**N.Stage**
N0	50	NA	NA	NA	NA	NA
N1	130	NA	NA	NA	NA	NA
Unknown	5	102	42	63	66	80
**M.Stage**
M0	85	NA	NA	NA	NA	NA
M1	5	NA	NA	NA	NA	NA
Unknown	95	102	42	63	66	80
**Stage**
I	21	73	NA	13	4	NA
II	152	0	NA	50	45	NA
III	4	0	NA	0	13	NA
IV	5	0	NA	0	6	NA
**Gender**
Female	83	NA	NA	NA	NA	NA
Male	102	NA	NA	NA	NA	NA
**Age**
>65	89	NA	NA	NA	NA	NA
< =65	96	NA	NA	NA	NA	NA
**Grade**
G1	NA	NA	NA	NA	2	NA
G2	NA	NA	NA	NA	35	NA
G3	NA	NA	NA	NA	30	NA
G4	NA	NA	NA	NA	1	NA

### Data Preprocessing

For scRNA-seq data, quality control was performed, where each gene expressed at least three cells and each cell expressed at least 250 genes. “PercentageFeatureSet” in SEURAT R package (29) was conducted to calculate the proportion of mitochondria and rRNA. Mitochondria fraction <30% and each cell expressing at least 500 genes were set to screen data. Samples from TCGA and UCSC Xena were combined and grouped by normal and tumor samples. “NormalizeBetweenArrays” in limma R package (30) was performed to normalize the data. Finally, 177 tumor samples and 167 normal samples remained with a total of 24,210 genes, and they were defined as TCGA-PAAD dataset. For GSE21501, GSE28735, GSE57495, GSE62452, and GSE85916 datasets, samples without clinical information were excluded, and only tumor samples remained. Probes were converted to gene symbol. “RemoveBatchEffect” function was conducted to remove batch effects and “NormalizeBetweenArrays” function was used to normalize the data.

### Dimensionality Reduction by UMAP

Three single-cell samples were merged and normalized by log-normalization. “FindVariableFeatures” function was conducted to identify highly variable genes. “ScaleData” function was used to scale data and principal component analysis (PCA) was applied to reduce dimensionality. “FindNeighbors” and “FindClusters” functions were performed to cluster cells under the conditions of dim = 40 and resolution = 0.5. The top 40 components were selected, and uniform manifold approximation and projection (UMAP) was used to further reduce dimensionality. UMAP is a nonlinear dimensionality-reduction technique for visualizing single-cell data and process high-dimensional data (31).

### Definition of Cell Types

Specific marker genes of different tissues were obtained from CellMarker database (http://biocc.hrbmu.edu.cn/CellMarker/). Markers of pancreas, pancreatic acinar tissue, fetal pancreas, peripheral blood, and blood were selected. By using enricher function in clusterProfiler R package (32), the enrichment score of these marker genes of each subgroup was calculated. According to the high or low enrichment of these marker genes in different subgroups, different cell types were defined. One cell type having multiple subgroups was classified into different cell types. “Minimum.spanning.tree” function was used to calculate the minimum distance between two cell types, and a minimum-cost spanning tree (MST) was constructed. MST and UMAP plots were used to estimate whether two subgroups could be combined.

### Analysis of Cell Trajectory and Identification of key Regulator Genes

Cell trajectory reflected the development time and differentiation degree of different cell types. Monocle 2 toolkit was applied to generate the trajectory of cell development (33). Monocle is an unsupervised algorithm for processing high-dimensionality data and dynamically presents single-cell data. Branched expression analysis modeling (BEAM) measurement in monocle 2 was employed to identify regulator genes (33). BEAM is a regression model for detecting critical genes involved in cell development.

### ReactomeGSA for Functional Analysis

When analyzing functional pathways of subgroups, ReactomeGSA R package (34) was implemented to calculate the enrichment score of each pathway in each subgroup. The top 20 differentially enriched pathways were selected. ReactomeGSA linking to reactome database can allow to analyze functional pathways from multi-omics and derive novel biomedical insights (34).

### Ligand–Receptor Interactions Analyzed by CellPhoneDB

CellPhoneDB, a public tool with curated receptors and ligands, was applied to assess cell–cell communication (35). Databases, including UniProt, Ensembl, PDB, the IMEx consortium, and IUPHAR, were utilized by CellPhoneDB for comprehensively assessing ligand–receptor interactions. CellPhoneDB is convenient to screen important interactions between different cell types for single-cell data. Mean expression of each ligand–receptor and their *p-*values were calculated. Ligand–receptor network was used to manifest the overall interactions among cell types. Thick and thin interactions between two cell types represent strong and weak interactions between them, respectively. Dot plots presenting specific ligand–receptor pairs were used to display interactions between specific cell types under a condition of mean interaction >1.

### Constructing Protein–Protein Interaction Network

Univariate Cox regression analysis was performed to screen marker genes of C4, C10, C16, and C18 associated with prognosis (*p* < 0.001). For these screened genes, rcorr function in Hmisc R package (https://cran.r-project.org/web/packages/Hmisc/index.html) was applied to analyze the correlation among each genes. Correlation coefficient| > 0.9 and *p* < 0.001 were selected to screen gene pairs. Based on the screened marker genes associated with prognosis, STRING database (https://string-db.org/) and Cytoscape software (version 3.6.1) (36) were introduced to construct protein–protein interaction (PPI) network for identifying hub genes involved in cancer development.

### Statistical Analysis

All statistical analyses were performed in R platform (version 3.4.2). Student's *t-*test was conducted to test the significance of expression between two groups. ANOVA was performed to test the distribution of cell types between normal and tumor samples. Log-rank test was conducted in the Kaplan–Meier survival analysis and univariate Cox regression analysis. *p* < 0.05 was considered as significant. All parameters of tools not specifically shown were default.

## Results

### Clustering Single-Cell Data of Pancreatic Cancer Based on Dimensionality Reduction

Single-cell data (GSE165399), including three samples (one normal samples and two tumor samples), was preprocessed by SEURAT R package. Parameters of one gene expressing at least in three cells and one cell expressing at least 250 genes were set to screen gene expression data. “PercentageFeatureSet” function was conducted to calculate the proportion of mitochondria and rRNA in cells. One cell expressing at least 500 genes with not more than 30% percentile mitochondria was set to ensure the quality. The quality control and total cell counts before and after filtration were displayed (Supplementary Figures S1–S3). Then, data of three samples were merged and normalized by log-normalization. High-variable genes were identified by “FindVariableFeatures” function, and the top 20 high-variable genes were shown (Supplementary Figure S4).

**Figure 2:**
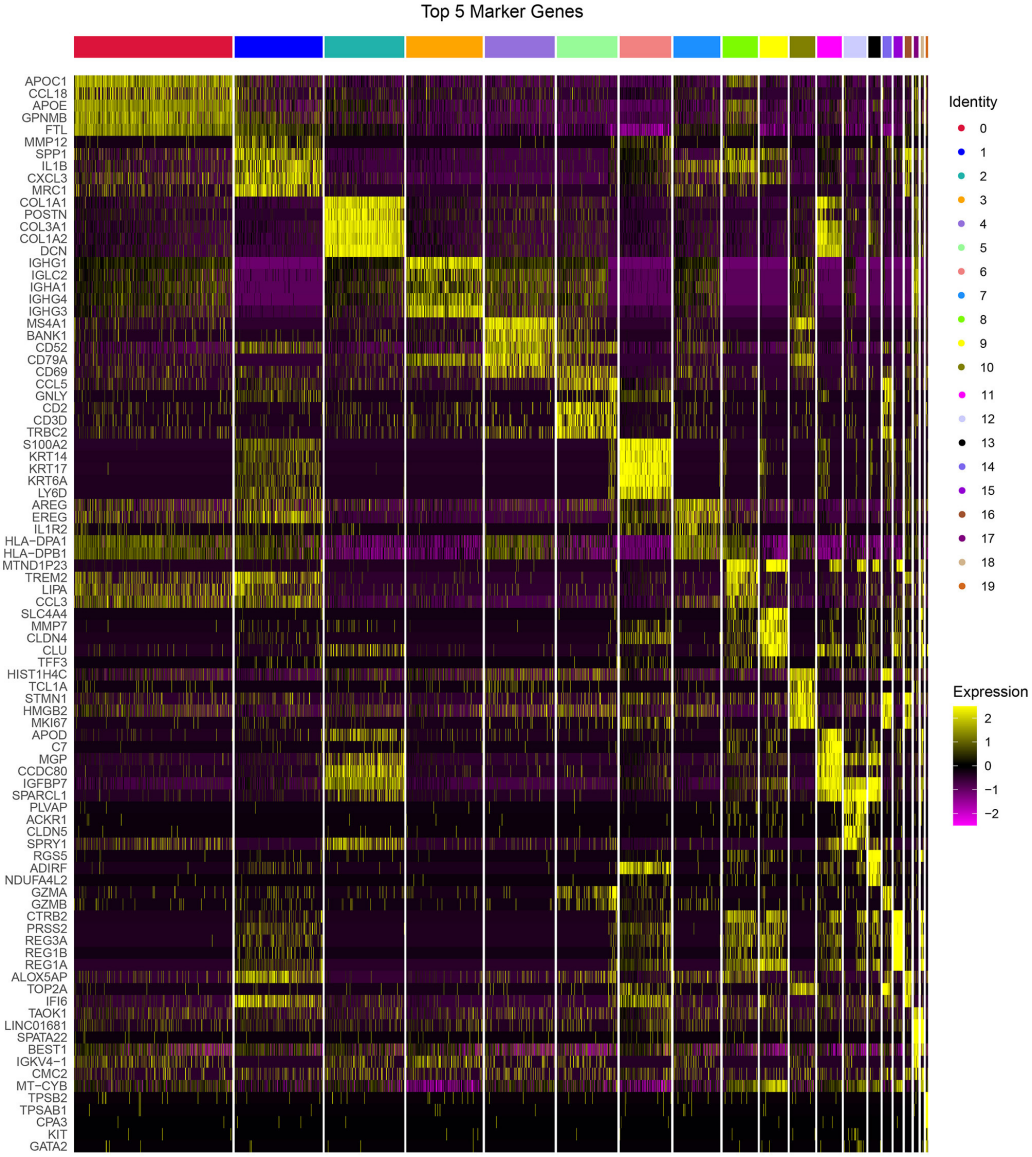
The top 5 markers of 20 subgroups. Yellow represents relatively high expression and plum represents relatively low expression.

The PCA was performed to construct a two-dimensional distribution of gene expression (Supplementary Figure S5). Then, function of “FindNeighbors” and “FindClusters” was performed to cluster cells, and 20 subgroups were generated. The dimensionality of expression data was further reduced by UMAP function based on the top 40 components (Figures 1B,C). In total, 20 subgroups from C0 to C19 were identified, and their proportions in each sample were shown (Figures 1D,E). We observed that three samples had significantly different distribution of 20 subgroups even within two tumor samples, indicating that there was a difference in cancer development within difference cancer types. Using “FindAllMarkers” function, we screened the marker genes of each subgroup significantly differential expressed among the subgroups (*p* < 0.05), and only the top five marker genes were exhibited (Figure 2).

### Defining Cell Types for 20 Subgroups

To accurately classify 20 subgroups into different cell types, we downloaded cell markers of specific tissues, including pancreas, pancreatic acinar tissue, fetal pancreas, peripheral blood, and blood, from CellMarker database. By implementing “enricher” function in clusterProfiler R package, 20 subgroups were defined into 12 different cell types, and 6 out of 12 cell types could be further classified into different cell subtypes (Table 2).

**Table 2:** The information of 20 cell types.

**Seraut cluster**	**Cell types**	**New cell types**
0	CD1C-CD141- dendritic cell	CD1C-CD141- dendritic cell_1
1	CD1C-CD141- dendritic cell	CD1C-CD141- dendritic cell_2
2	Fibroblast	Fibroblast_1
3	Plasmacytoid dendritic cell	Plasmacytoid dendritic cell
4	B cell	B cell_1
5	Natural killer cell	Natural killer cell_1
6	CD1C+_B dendritic cell	CD1C+_B dendritic cell_1
7	CD1C+_B dendritic cell	CD1C+_B dendritic cell_2
8	Myeloid dendritic cell	Myeloid dendritic cell
9	Cancer cell	Cancer cell_1
10	B cell	B cell_2
11	Fibroblast	Fibroblast_2
12	Endothelial cell	Endothelial cell
13	AXL+SIGLEC6+ dendritic cell	AXL+SIGLEC6+ dendritic cell
14	Natural killer cell	Natural killer cell_2
15	Cancer cell	Cancer cell_2
16	CD1C+_B dendritic cell	CD1C+_B dendritic cell_3
17	Beta cell	Beta cell
18	Cancer cell	Cancer cell_3
19	Basophil	Basophil

**Figure 3:**
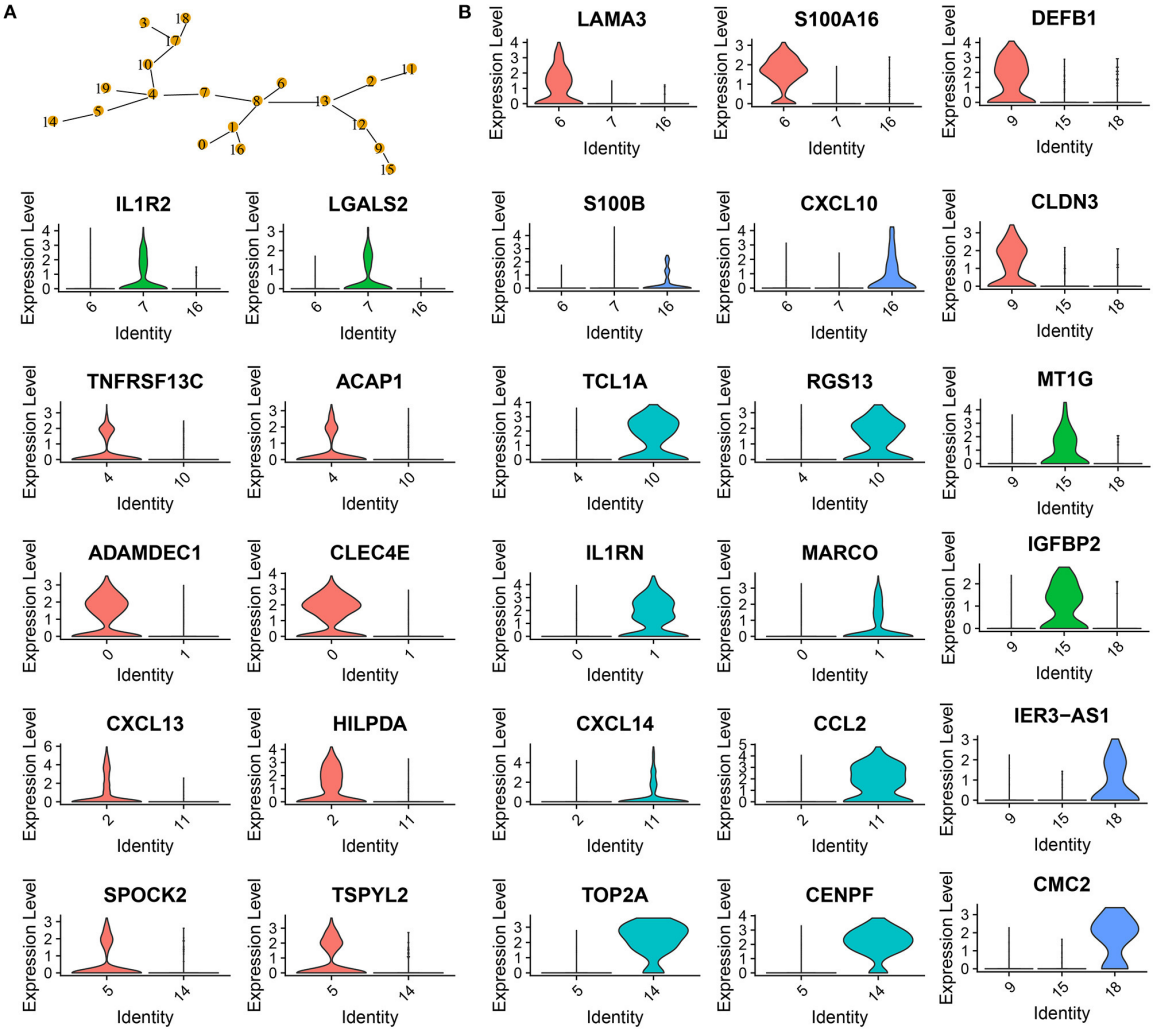
Confirmation of 20 subgroups. **(A)** MST of 20 subgroups. Yellow points labeled with different numbers represents different subgroups. **(B)** Violin plots showing marker genes expressed in different subgroups.

To further classify the 6 cell types, MST was used to calculate the minimum distance between the two subgroups (Figure 3A). C4 and C10 could not be combined, as the closest distance to C4 was C19. C4 and C10 were defined as B cell_1 and B cell_2, respectively. Cancer cells had three subgroups (C9, C15, and C18), where C18 was the closest to C17, and C9 was close to both C12 and C15. Therefore, three subgroups of cancer cells were defined into three cell types. CD1C-CD141-dendritic cells and CD1C+_B dendritic cells (DCs) had multiple subgroups, but their distance was not close. Fibroblasts (C2 and C11) were close to C13, and natural killer cells (C5 and C14) were close to C4. The multiple branches of C4 and C13 indicated that they may develop into different subgroups. The development of one cell type was affected by the cancer microenvironment; therefore, we did not merge different subgroups in both fibroblasts and natural killer cells.

Analysis of marker genes in these cell types demonstrated that different cell types expressed specific markers, supporting that these subgroups should be classified into different cell types (Figure 3B). For example, CD1C+_B dendritic cell_1 specifically expressed LAMA3 and S100A16, CD1C+_B dendritic cell_2 specifically expressed IL1R2 and LGALS2, and CD1C+_B dendritic cell_3 specifically expressed S100B and CXCL10 (Figure 3B).

### Cell Development and Key Regulatory Genes of 20 Subgroups

**Figure 4:**
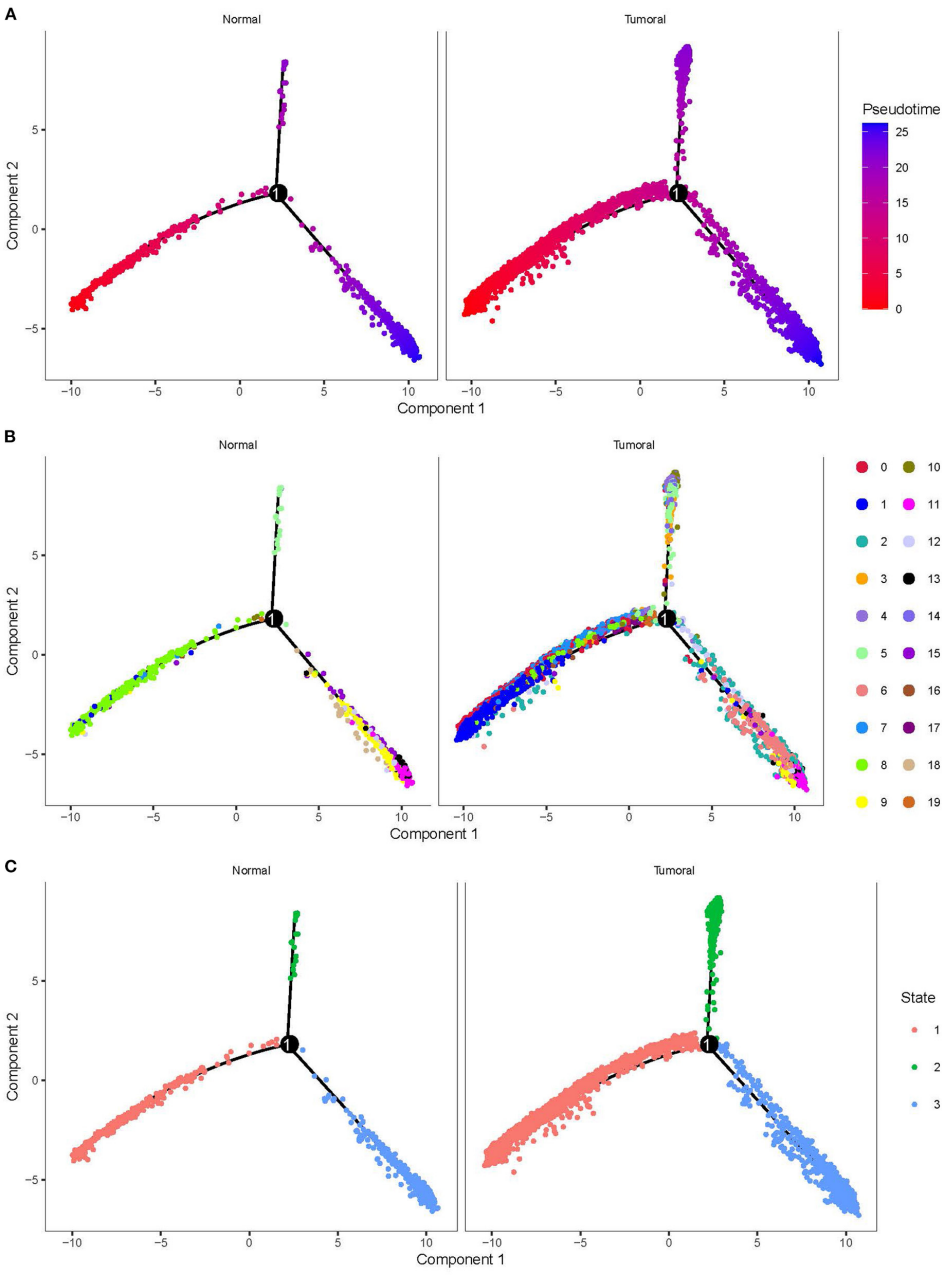
Cell trajectory of 20 subgroups grouped by normal and tumoral. **(A)** Pseudotime of different subgroups. Colors from red to blue indicates pseudotime from early to late. **(B)** The location of 20 subgroups in the trajectory. **(C)** Three states with different colors defined by three branches.

To delineate the cell development of different cell types, we applied monocle to predict their cell trajectory. Pseudotime was used to evaluate the degree of their cell division, and 20 subgroups were divided into three major branches (Figure 4). An obvious difference of cell distribution was observed between normal and tumor samples. Tumor samples had a significantly higher proportion of cells locating in the early pseudotime than the normal sample, suggesting that tumor samples had a large number of undifferentiated or incompletely differentiated cells, which probably led to high potential of cell proliferation. Three branches were defined as three different status (states 1, 2, and 3).

By using branched expression analysis modeling, we then screened a series of regulatory genes of three branches. The result showed a total of 127 key regulatory genes highly associated with cell development (corrected *p* < 0.0001). Gene expression of 127 regulatory genes were shown in a heatmap grouped by three status (Supplementary Figure S6). We found that the expression of 127 genes significantly varied by the pseudotime, indicating that these 127 genes may serve as key regulators in the development of different cell types. There was also an obvious difference in the expression pattern of 127 genes among three branches, with cluster 1 mostly expressed in state 2, cluster 2 mostly expressed in states 1 and 3, and cluster 3 mostly expressed in state 3 (Supplementary Figure S7). The distribution of different 20 cell types in the three branches is shown in Table 3. State 1 included the majority of CD1C-CD141- DCs, CD1C+_B dendritic cell_2, myeloid DCs, CD1C+_B dendritic cell_3, and beta cells. Plasmacytoid DCs, B cells, and natural killer cells were mostly accumulated in state 2. Fibroblasts, CD1C+_B dendritic cell_1, cancer cells, endothelial cells, and AXL+SIGLEC6+ DCs were mostly enriched in state 3. The results suggested that these regulatory genes may play different roles in the different status of cancer cell development.

**Table 3:** The cell counts of 20 subgroups in three types of status.

**Cell type**	**State 1**	**State 2**	**State 3**
0	1,712	3	2
1	951	0	4
2	19	1	842
3	64	762	1
4	19	736	3
5	31	623	3
6	2	0	558
7	473	35	1
8	381	0	1
9	4	0	297
10	21	253	2
11	0	0	265
12	10	1	231
13	0	0	132
14	4	92	1
15	3	0	94
16	74	0	0
17	44	8	0
18	1	0	33
19	13	12	0

### ReactomeGSA for Identifying Differential Enriched Pathways

**Figure 5:**
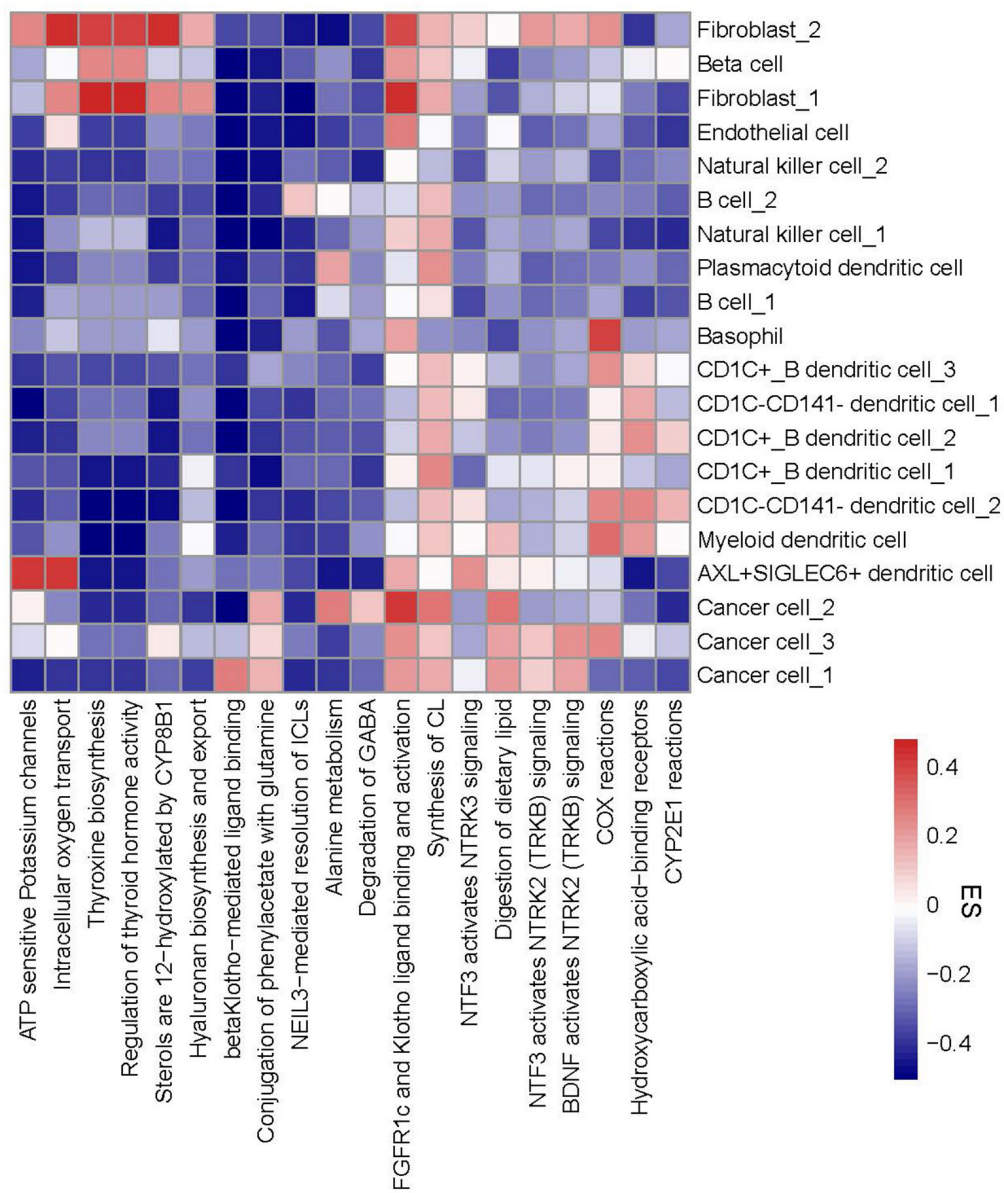
A heatmap of functional pathways differentially enriched in 20 subgroups. Enrichment score from high to low was labeled as colors from red to blue. The right line indicates 20 subgroups and the bottom line indicates 20 functional pathways. ES, enrichment score.

To evaluate functional pathways of 20 cell types, we applied ReactomeGSA linking to reactome database to analyze the enrichment score of each pathway in each cell type. The top 20 differentially enriched pathways of 20 cell types were visualized in a heatmap (Figure 5). We observed that different cell types in one cell type manifested different enrichment patterns of these pathways. For instance, CD1C-CD141- dendritic cell_2 had two more enriched pathways (COX reactions and CYP2E1 reactions) than CD1C-CD141- dendritic cell_1. Three subgroups of cancer cells exhibited obviously different enrichment in six pathways, for example, beta Klotho-mediated ligand binding was enriched in cancer cell_1, alanine metabolism and degradation of GABA were enriched in cancer cell_2, and COX reactions were enriched in cancer cell_3. The specific enrichment score of all 20 pathways in each subgroup was also displayed (Supplementary Figure S8).

### Differential Distribution of Subgroups Between Normal and Tumor Samples

**Figure 6:**
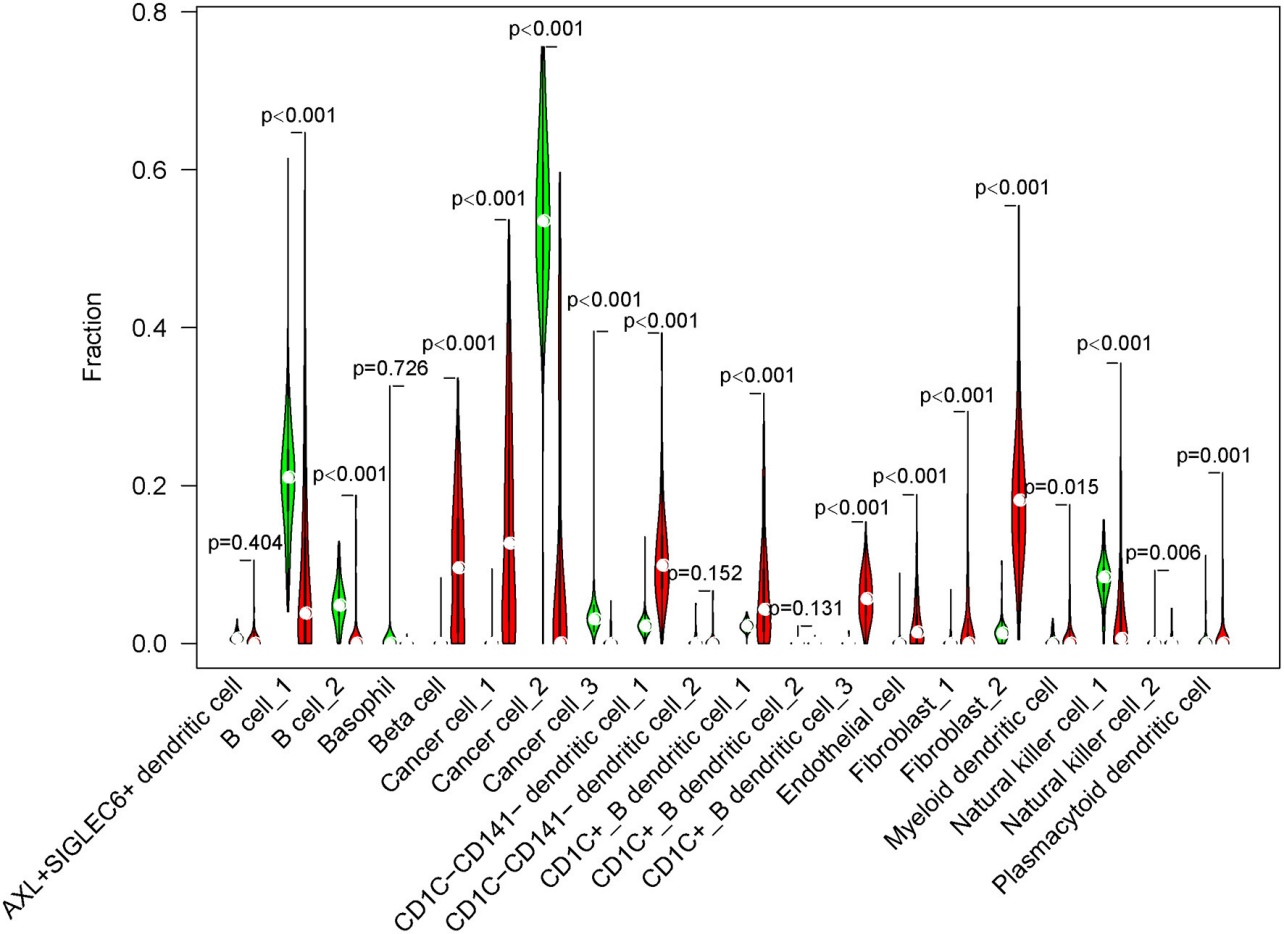
The fraction of 20 cell subgroups in TCGA-PAAD dataset. Red indicates tumor samples and green indicates normal samples.

Next, we used CIBERSORT measurement to analyze the proportion of 20 subgroups in normal cells and tumor cells in an independent dataset (TCGA-PAAD) according to the screened marker genes in single-cell data. The results showed that 16 of 20 subgroups were differentially enriched between normal cells and tumor cells (*p* < 0.01, Figure 6). B cells, cancer cell_2, cancer cell_3, and natural killer cell_1 were more enriched in normal cells, whereas beta cells, cancer cell_1, DCs, endothelial cells, and fibroblasts were more enriched in tumor cells.

**Figure 7:**
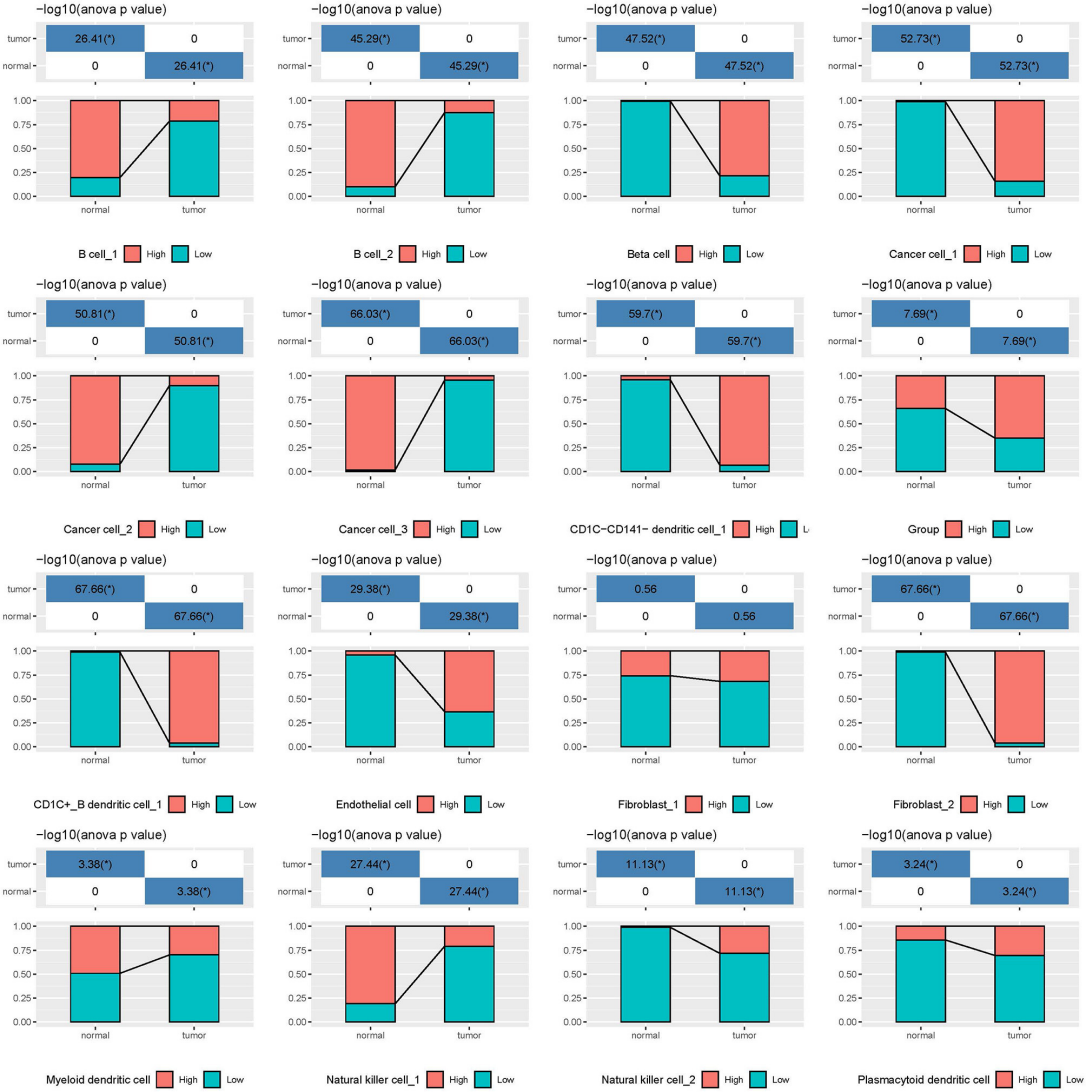
The independent proportion of 16 subgroups in normal and tumor samples. High and low indicates the enrichment of cell types. *p < 0.05.

In the independent comparison of the proportion of 16 subgroups between normal and tumor cells, we found that except for fibroblast_1, 15 out of 16 subgroups were significantly distributed between them (*p* < 0.05, Figure 7). Interestingly, normal cells had limited proportion of beta cells, cancer cell_1, CD1C-CD141- dendritic cell_1, CD1C+_B dendritic cell_1, endothelial cells, fibroblast_2, and natural killer cell_2. The proportion of these cell types were all markedly elevated in tumor samples. Furthermore, we analyzed the relation between 16 subgroups and survival in tumor samples, and observed that only 4 subgroups were associated with prognosis (*p* < 0.05, Supplementary Figure S9). B cell_1, B cell_2, and cancer cell_3 were positively associated with prognosis, whereas low enrichment of CD1C+_B dendritic cell_3 had more favorable prognosis. We considered that these four cell types possibly played critical roles in cancer development.

### Ligand–Receptor Interactions Among Different Subgroups

**Figure 8:**
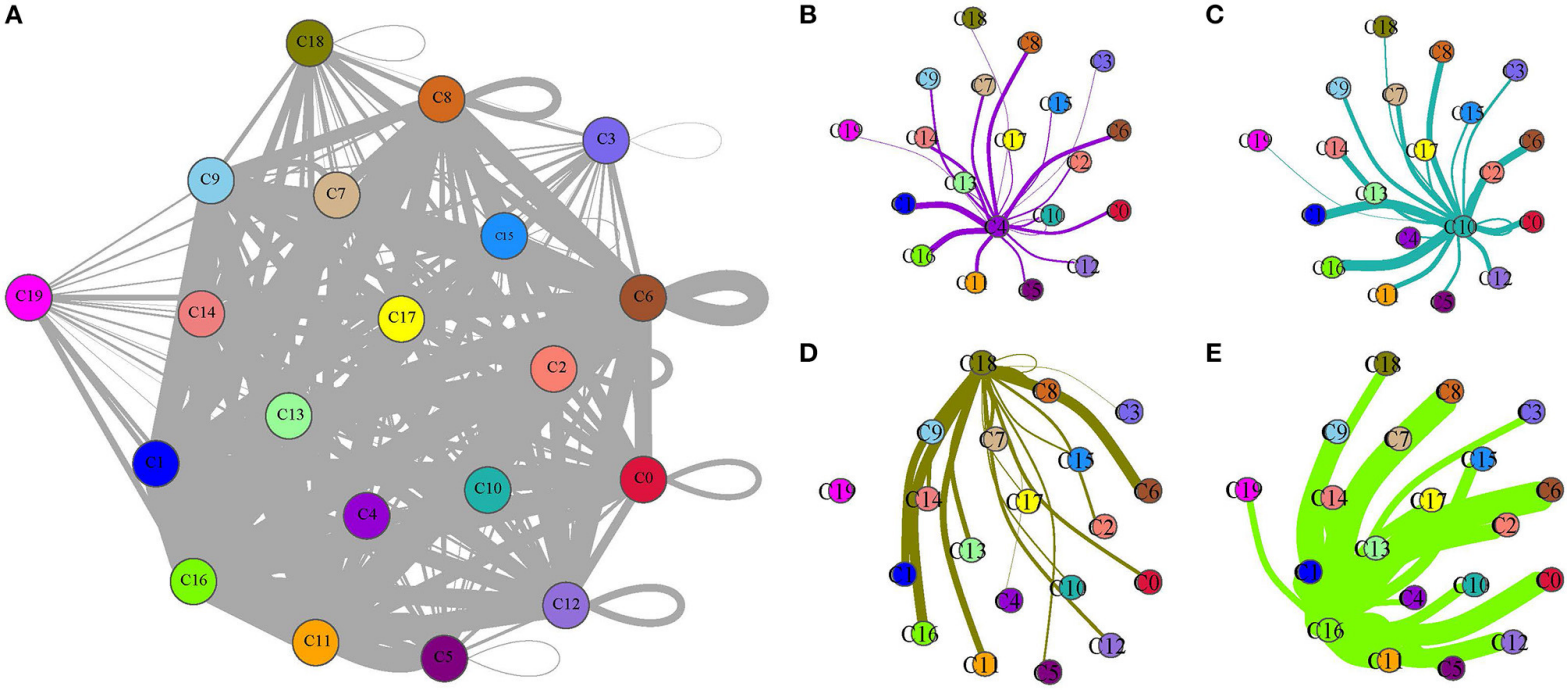
Ligand–receptor interactions among 20 subgroups analyzed by CellPhoneDB. **(A)** A network of ligand–receptor interactions among 20 subgroups. **(B–E)** Ligand–receptor interactions of B cell_1 (C4), B cell_2 (C10), cancer cell_3 (C18), and CD1C+_B dendritic cell_3 (C16) to other subgroups, respectively. Circles with different colors indicate different subgroups. Thick lines and fine lines between subgroups indicate strong and weak interactions between them.

As B cell_1 (C4), B cell_2 (C10), cancer cell_3 (C18), and CD1C+_B dendritic cell_3 (C16) were identified to be significantly associated with prognosis, we therefore analyzed their interactions among them and with other subgroups based on CellPhoneDB. The ligand–receptor interactions among 20 cell types were displayed in a network (Figure 8A). The interactions of four cell types (C4, C10, C18, and C16) with other types were independently displayed (Figures 8B–E). Within these four cell types, CD1C+_B dendritic cell_3 (C16) had the most interactions with other types, especially CD1C-CD141- dendritic cell_1 (C0), CD1C+_B dendritic cell_1 (C6), and myeloid DC (C8) (Figure 8E). Both B cell_1 (C4) and B cell_2 (C10) had close interactions with CD1C-CD141- dendritic cell_2 (C1) and CD1C+_B dendritic cell_3 (C16) (Figures 8B,C). Cancer cell_3 (C18) strongly interacted with CD1C+_B dendritic cell_1 (C6) and CD1C+_B dendritic cell_3 (C16) (Figure 8D).

**Figure 9:**
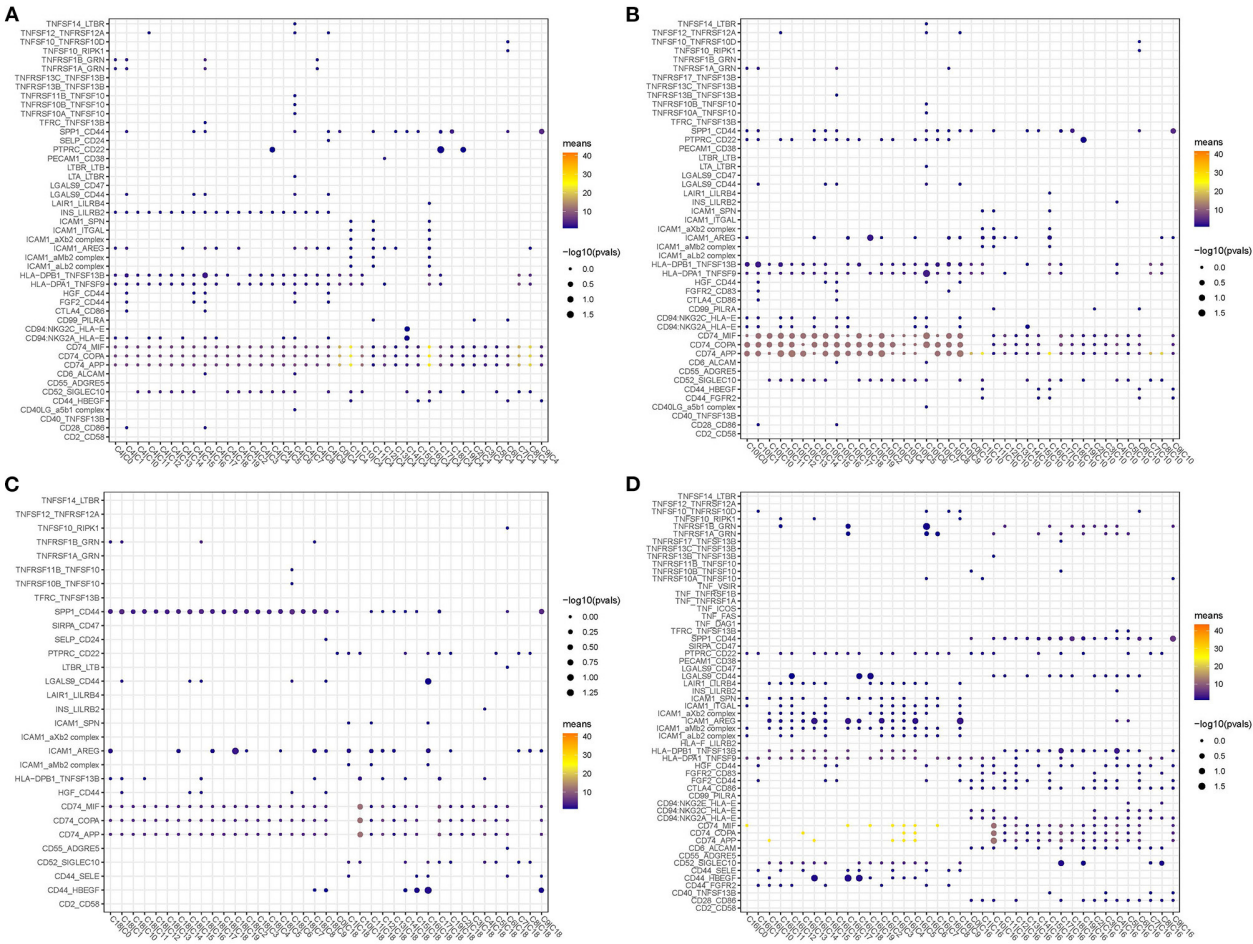
Dot plots of specific ligand–receptor interactions of C4 **(A)**, C10 **(B)**, C18 **(C)**, and C16 **(D)** to other subgroups.

In addition, we screened the specific ligand–receptor interactions of four cell types to others under the condition of averaged interaction value >1 (Figure 9). Three ligand–receptor pairs, including CD74_MIF, CD74_COPA, and CD74_APP, were the most active among these interactions. Overall, these four cell types had close interactions with DCs, indicating that different types of DCs probably played important roles in constructing microenvironment beneficial to tumor growth.

### Screening the hub Gene BUB1 of Pancreatic Cancer Through PPI Networks

**Figure 10:**
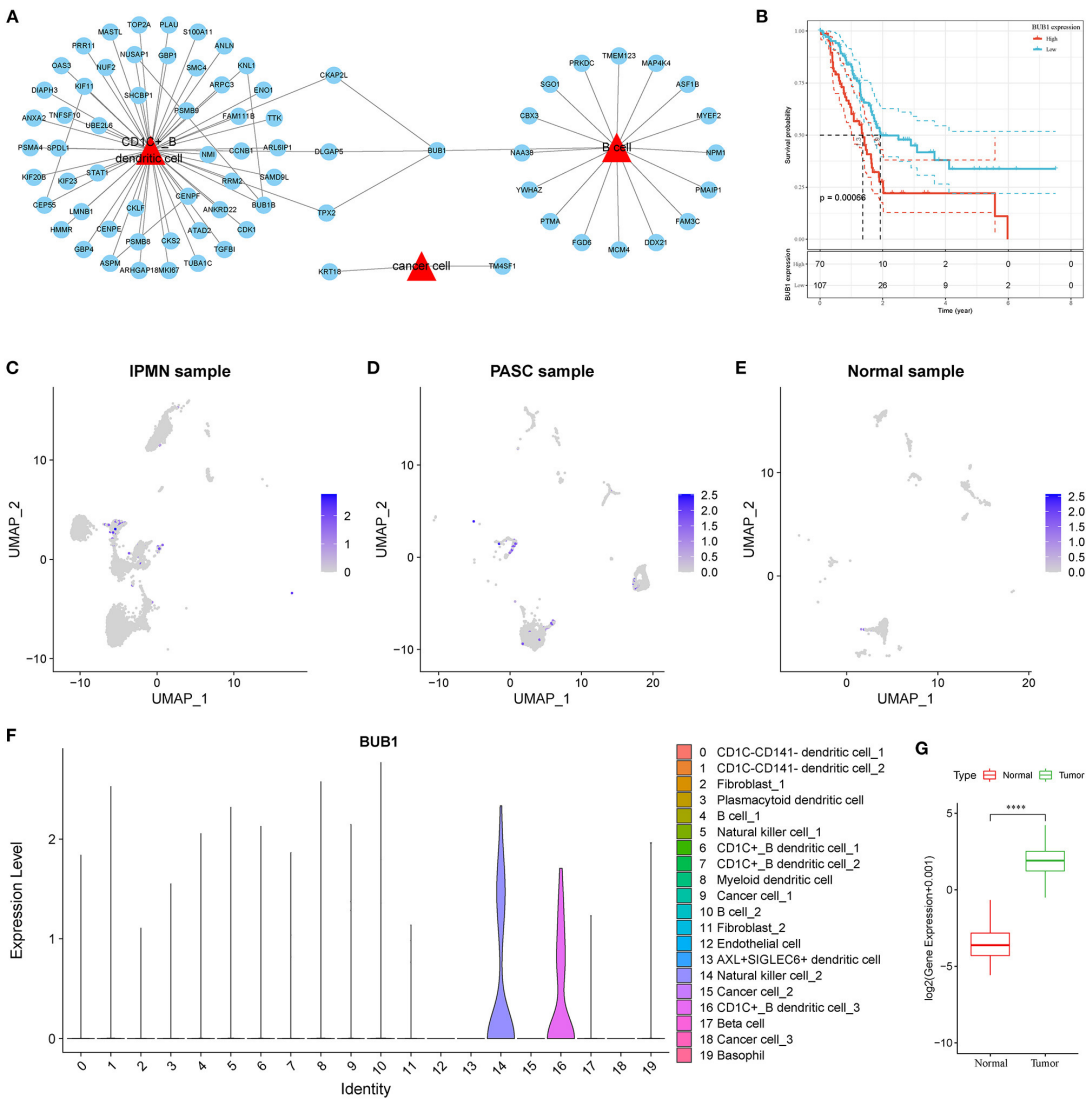
Screening of hub genes highly contributing to cancer development. **(A)** A PPI network of marker genes of C4, C10, C16 and C18. Red triangle indicates cell types and blue circle indicates marker genes. **(B)** Kaplan–Meier survival plot of BUB1 grouped by high and low expression. **(C–E)** UMAP plots showing distribution and expression of BUB1 in IPMN, PASC and normal samples. Colors from gray to blue indicate expression from low to high. **(F)** Violin plot of BUB1 expression in 20 subtypes. **(G)** Difference in expression and distribution of BUB1 between cancer and normal samples.

**Figure 11:**
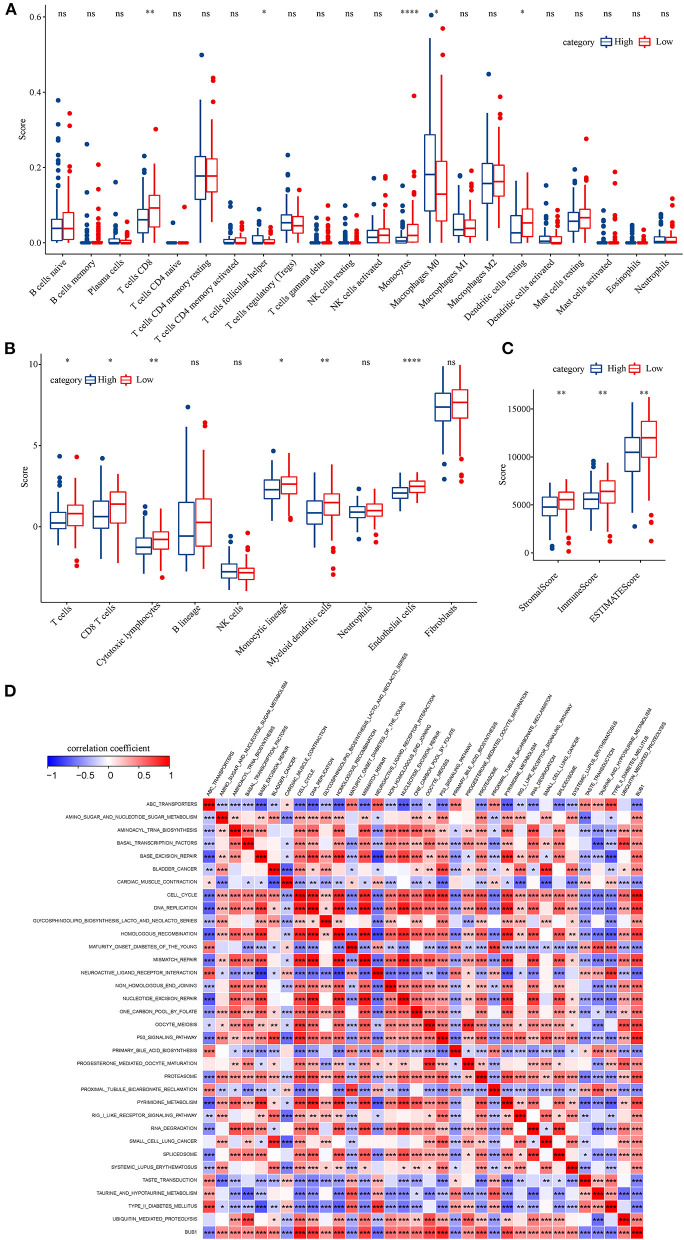
BUB1 expression and immune and functional analysis. **(A)** Distribution difference of 22 kinds of immune cell infiltration in patients with high and low expression of BUB1. **(B)** Distribution difference of immune cell infiltration in 10 kinds of patients with high and low expression of BUB1. **(C)** Differences in the distribution of immune infiltration in patients with high and low expression of BUB1. **(D)** BUB1 expression was significantly related to KEGG pathway.

To identify the hub genes responsible for cancer development, we focused on four cell types (C4, C10, C16, and C18) and conducted univariate Cox regression analysis for their marker genes. A total of 73 genes were strongly correlated with prognosis, with 2 marker genes in C4, 15 genes in C10, 54 genes in C16, and 2 genes in C18. Through conducting rcorr function in Hmisc package, the correlation and significance among these marker genes were calculated. In total, 14 pairs of marker genes were screened under |correlation coefficient > 0.9 and *p* < 0.001 (Supplementary Table S1). The PPI network revealed that *BUB1* gene was in the center, and it was the bridge to link CD1C+_B DCs and B cells (Figure 10A). Survival analysis of the relation between BUB1 expression and survival showed a significant association, with low BUB1 expression showing more favorable prognosis (*p* = 0.00066, Figure 10B). Moreover, we determined BUB1 expression in two tumor samples and one normal sample. It almost had no expression in the normal sample but was obviously expressed in tumor samples, specially expressed in natural killer cell_2 and CD1C+_B dendritic cell_3 (Figures 10C–F). Integrating the data of normal samples and tumor tissues of pancreas from GTEX and TCGA showed that BUB1 in tumor samples was significantly higher than that in normal samples (Figure 10G). In addition, in the transcriptome samples, we also analyzed the relationship between BUB1 expression and immunity. By evaluating the immune cell infiltration score of patients with ciberport and mcpcounter, we can observe that the monocytes, CD8 T cells, endothelial cells, cytotoxic lymphocytes, and T-cell infiltration of patients with low expression of BUB1 were significantly higher than that of patients with high expression of BUB1 (Figures 11A,B). Similarly, estimate was used to evaluate the immune microenvironment infiltration of patients, it can be observed that the immune microenvironment infiltration in patients with low BUB1 expression was significantly higher than that in patients with high BUB1 expression (Figure 11C). Furthermore, the enrichment score of each patient in Kyoto Encyclopedia of Genes and Genomes (KEGG) pathway was evaluated by single-sample gene set enrichment analysis. We calculated the correlation between BUB1 expression and pathway, selected KEGG pathway with significant correlation and correlation coefficient >0.4, and identified a total of 33 key pathways, which included cell_ Cycle, DNA_REPLICATION and MISMATCH_REPAIR and other important pathways related to cell proliferation, such as P53_SIGNALING_PATHWAY, SMALL_CELL_LUNG_CANCER and BLADDER_CANCER, and other pathways closely related to tumors (Figure 11D). These results showed that the abnormality of BUB1 was closely related to the occurrence and development of tumors. To validate the above results, we introduced other expression data, including GSE21501, GSE28735, GSE57495, GSE62452, and GSE85916, to evaluate BUB1 expression and its relation to prognosis. Five datasets were preprocessed and normalized for removing batch effects. The expression profiles of five datasets were combined without bias, and their distribution before and after preprocessing was shown in PCA plots (Supplementary Figures S10A,B). The Kaplan–Meier survival analysis demonstrated that low BUB1 expression was still significantly correlated with overall survival (*p* = 0.023, Supplementary Figure S10C). In addition, using SangerBox (http://vip.sangerbox.com) to analyze the relationship between BUB1 expression and clinical features, we observed that although there was no significant expression difference of BUB1 in different clinical stages, but there were significant expression differences in patients with different depth of invasion (Supplementary Figure S10D). We also evaluated the relationship between the expression of BUB1 in Pan-cancer and prognosis. It can be observed that BUB1 had a significant prognostic correlation in many other tumors, such as renal cancer, glioma, liver cancer, leukemia, lung cancer, and so on (Supplementary Figure S10E), indicating that BUB1 acted as an essential role in pancreatic cancer.

## Discussion

The application of single-cell sequencing technology improves the past understanding of tumor microenvironment (TME), pathological process, molecular characteristics in different subtypes of pancreatic cancer (21, 23). In the current study, we characterized the distribution of different cell types between normal samples and tumor samples, and aimed to identify a molecular target highly associated with pancreatic cancer development for potentially used in drug exploration.

We included three samples (IPMN, PASC, and normal samples) with scRNA-seq data and identified 20 subgroups (C0 to C19). Obvious differences of subgroup distribution among three samples were observed, indicating the possible distinct mechanisms of cancer development between IPMN and PASC and great alternation of TME. In the normal pancreatic tissue, myeloid DCs (C8), cancer cell_1 (C9), fibroblast_2 (C11), and endothelial cells (C12) contributed to an over 3/4 proportion of all cells. On the contrary, these cell types were less enriched or almost disappeared in two tumor samples. In IPMN, CD1C-CD141- dendritic cell_1 (C0), fibroblast_1 (C2), plasmacytoid DCs (C3), B cell_1 (C4), and natural killer cell_1 (C5) were the most enriched. In PASC, two cell types of CD1C-CD141- dendritic cell_2 (C1) and CD1C+_B dendritic cell_1 (C6) consisted of about 3/4 in all cell types. Notably, (DCs comprise a majority number in all cell types, and it largely varied in these three samples, suggesting that DCs underwent immense transformation or abnormal division during tumorigenesis.

A line of studies have demonstrated that DCs play key roles in activating immune response in tumors, such as capturing antigens, processing, and presenting them as antigenic peptides to T cells (37). However, its function can be impaired by tumors through suppressing DC accumulation, activation, and antigen presentation (38). With the exploring study on DCs, DCs have been considered as promising vaccines for integrating tumor peptides and presenting antigens against tumors (37). In the present study, different types of DCs in TME may play different roles in tumor development.

The distinct distribution of cell types between normal and tumor samples was also validated in the independent dataset. Importantly, we discovered that four cell types (B cell_1, B cell_2, cancer cell_3, and CD1C+_B dendritic cell_3) were significantly associated with prognosis. We assumed that these four cell types may be essential in tumor progression. Therefore, ligand–receptor interactions among different cell types were analyzed. Within the above four cell types, CD1C+_B dendritic cell_3 was found to have strong cell–cell communications with other cell types. Especially, three ligand–receptor pairs, including CD74-MIF, CD74-COPA, and CD74-APP, were much more enriched in the interactions of CD1C+_B dendritic cell_3 to other cell types.

CD74-MIF has been reported to regulate immune activity (39). CD74 is an essential receptor to DCs for their migration and mediating immune response and regulates the development of T and B cells (40). Macrophage migration inhibitory factor (MIF) is a key cytokine involving in inflammatory diseases such as pulmonary inflammation through CD74 signaling (41). Tanese et al. (42) found that CD74-MIF serves as mediators to activate PI3K/AKT signaling pathway in melanoma. Moreover, a study illustrated that blockading CD74-MIF on macrophages and DCs can recover antitumor activity of immune system in metastatic melanoma (43). Apart from melanoma, CD74 and MIF have been considered as therapeutic targets in other cancer types such as prostate cancer and gastric cancer (44, 45). In pancreatic cancer, CD74-MIF is possibly a promising target for molecular therapy, but further experimental study is needed. CD74-COPA and CD74-APP were seldom reported, so their high enrichment also indicated the important function in tumor progression in pancreatic cancer, but this needs further verification.

As four cell types were identified to be associated with prognosis, we considered that there were key marker genes in them regulating tumorigenesis in pancreatic cancer. Therefore, the marker genes of B cell_1, B cell_2, cancer cell_3, and CD1C+_B dendritic cell_3 were screened by regression analysis. Through correlation analysis and constructing the PPI network, we discovered that *BUB1* gene displayed as a bridge to communicate CD1C+_B dendritic cell_3 and B cells.

BUB1 is a multitask protein kinase for chromosome segregation in eukaryotes. BUB1 impairment or dysregulation leads to chromosomal instability and results in tumorigenesis (46, 47). BUB1 has been widely reported to be associated with tumorigenesis in various cancer types including gastric cancer (48), breast cancer (49), and pancreatic ductal adenocarcinoma (50). Piao et al. (50) proved that high expression of BUB1 was associated with worse overall survival of pancreatic ductal adenocarcinoma, which was consistent with our observation. BUB1 could serve as a biomarker for predicting prognosis of patients with pancreatic cancer, as it also presented robust performance in the independent dataset. Importantly, we discovered that BUB1 was specifically expressed in natural killer cell_2 and CD1C+_B dendritic cell_3, suggesting that BUB1 probably played a critical role in modulating the expression of MIF that had close relation to tumorigenesis. To date, no study has reported this novel possible mechanism in cancer development. Therefore, BUB1 had great potential to be a therapeutic target for molecular therapy, but this needs more evidence in cell or animal experiments.

Immunotherapy is recognized as a promising strategy in many cancer types. According to our observations, DCs play a critical role against tumor cells, and they were impaired in TME, probably resulted from the dysregulation of cytokines or chemokines in TME. Currently, surgery is the only treatment with curative possibility for patients with pancreatic cancer. Considering the insensitivity of pancreatic cancer to chemotherapy and radiotherapy, other molecular-targeted drugs are particularly needed. In this study, CD74-MIF and BUB1 were found to be potential therapeutic targets for treating pancreatic cancer.

In conclusion, this study identified four cell types that are significantly associated with pancreatic cancer prognosis based on single-cell data and integrated bioinformatics analysis. Cell types largely varied in different cancer types and between normal and tumor samples. DCs, especially CD1C+_B dendritic cell_3, played a critical role in cancer progression probably through CD74 signaling. Three pairs of ligand–receptor interactions (CD74-MIF, CD74-COPA, and CD74-APP) were considered to be closely involved in tumorigenesis. Importantly, BUB1 could serve as a biomarker for predicting prognosis and a therapeutic target for treating pancreatic cancer, but this needs further experiments.

## Data Availability Statement

The datasets presented in this study can be found in online repositories. The names of the repository/repositories and accession number(s) can be found in the article/Supplementary Material.

## Author Contributions

DW and JS designed the study, reviewed, and edited the manuscript. MY contributed to the literature research. ZZ, XC, and JS analyzed and interpreted the data. ML, XD, and YX wrote the initial draft of the manuscript. All the authors read and approved the manuscript and its publication. All authors contributed to the completion of this study.

## Conflict of Interest

YX, ZZ, XC, and DW were employed by YuceBio Technology Co., Ltd. The remaining authors declare that the research was conducted in the absence of any commercial or financial relationships that could be construed as a potential conflict of interest.

## Publisher's Note

All claims expressed in this article are solely those of the authors and do not necessarily represent those of their affiliated organizations, or those of the publisher, the editors and the reviewers. Any product that may be evaluated in this article, or claim that may be made by its manufacturer, is not guaranteed or endorsed by the publisher.
