# Supplementary material for: BUB1 Is Identified as a Potential Therapeutic Target for Pancreatic Cancer Treatment
Source: Front Public Health. 2022 Jun 13;10:900853. doi: 10.3389/fpubh.2022.900853 (PMC9235519; doi:10.3389/fpubh.2022.900853)
Supplement: Supplementary file 1 [file Table_1.DOCX]

Supplementary Table S1. The correlation of 14 gene pairs associated with prognosis.

| Gene 1 | Gene 2 | Correlation | *P* |
| --- | --- | --- | --- |
| *ASPM* | *CENPF* | 0.904983419 | 0 |
| *BUB1* | *CKAP2L* | 0.928032163 | 0 |
| *BUB1* | *DLGAP5* | 0.917780354 | 0 |
| *BUB1* | *TPX2* | 0.905596673 | 0 |
| *BUB1B* | *KNL1* | 0.912419713 | 0 |
| *BUB1B* | *NUSAP1* | 0.908640759 | 0 |
| *CENPF* | *ASPM* | 0.904983419 | 0 |
| *CEP55* | *KIF11* | 0.906489561 | 0 |
| *CKAP2L* | *BUB1* | 0.928032163 | 0 |
| *DLGAP5* | *BUB1* | 0.917780354 | 0 |
| *KIF11* | *CEP55* | 0.906489561 | 0 |
| *KNL1* | *BUB1B* | 0.912419713 | 0 |
| *NUSAP1* | *BUB1B* | 0.908640759 | 0 |
| *TPX2* | *BUB1* | 0.905596673 | 0 |
