# Supplementary material for: BUB1 Is Identified as a Potential Therapeutic Target for Pancreatic Cancer Treatment
Source: Front Public Health. 2022 Jun 13;10:900853. doi: 10.3389/fpubh.2022.900853 (PMC9235519; doi:10.3389/fpubh.2022.900853)
Supplement: Supplementary file 2 [file Table_2.docx]

options(stringsAsFactors = F)

source('/pub1/data/mg_projects/projects/codes/mg_base.R')

#单细胞####

library(Seurat)

library(dplyr)

library(ggplot2)

library(magrittr)

library(gtools)

library(stringr)

library(Matrix)

library(tidyverse)

library(patchwork)

library(data.table)

library(RColorBrewer)

library(ggpubr)

#1、读取数据####

#非10x的数据

matrix_to_seurat <- function(file_id){

file_name = paste("origin_datas/scRNA/",file_id,"_matrix.txt.gz",sep ="")

counts=fread(file = file_name,data.table = T,sep = '\t',check.names = F)

counts=data.frame(counts)

rownames(counts)=counts[,1]

counts=counts[,-1]

# 在细胞前添加样本名

colnames(counts)=paste0(file_id,"_",colnames(counts))

genename <- rownames(counts)

# 将基因名中"_"替换为"-",防止seurat识别的更改

genename<-gsub("_","-",genename)

rownames(counts)=genename

# 构建seurat对象，保留至少在3个细胞中表达的基因 & 至少有250基因表达的细胞

sce <- CreateSeuratObject(counts=counts,project = file_id,min.cells = 3, min.features = 250)

return(sce)

}

file_id <- list.files("origin_datas/scRNA/",pattern='_matrix.txt.gz$', full=FALSE)

file_id<- gsub("_matrix.txt.gz","",file_id)

datalist=list()

for (i in 1:length(file_id)){

datalist[[i]]=matrix_to_seurat(file_id[i])}

names(datalist)=file_id

#2、数据整合及质控####

for (i in 1:length(datalist)){

sce <- datalist[[i]]

sce[["percent.mt"]] <- PercentageFeatureSet(sce, pattern = "^MT-")# 计算线粒体占比

sce[["percent.Ribo"]] <- PercentageFeatureSet(sce, pattern = "^RP[SL]")# 计算rRNA占比

datalist[[i]] <- sce

rm(sce)

}

#质控前的

violin=list()

for (i in 1:length(datalist)){

violin[[i]] <- VlnPlot(datalist[[i]],

features = c("nFeature_RNA", "nCount_RNA", "percent.mt","percent.Ribo"),

pt.size = 0.1,

ncol = 4)

}

pearplot_befor <- CombinePlots(plots = violin , nrow=length(datalist), legend="none")

pearplot_befor

savePDF(filename = 'PDFs/QC_before.pdf',plot = pearplot_befor,he=15,wi=15)

sce <- merge(datalist[[1]],y=datalist[2:length(datalist)])

raw_count <- table(sce@meta.data$orig.ident)

table(sce@meta.data$orig.ident)

rm (sce)

#过滤

datalist <- lapply(X = datalist, FUN = function(x) {

x<-subset(x,subset = nFeature_RNA > 500 &

nFeature_RNA < 6000 &

quantile(percent.mt, 0.98) > percent.mt & percent.mt < 35 &

quantile(percent.Ribo, 0.99) > percent.Ribo & percent.Ribo > quantile(percent.Ribo, 0.01) &

nCount_RNA < quantile(nCount_RNA, 0.97) & nCount_RNA > 1000 )

})

#合并数据

sce <- merge(datalist[[1]],y=datalist[2:length(datalist)])

clean_count <- table(sce@meta.data$orig.ident)

table(sce@meta.data$orig.ident)

#GSM5032771 GSM5032772 GSM5032773

#5720 2064 1038

#过滤前后样本细胞数据的统计

summary_cells <- as.data.frame(cbind(raw_count,clean_count))

counts <- rbind(as.data.frame(cbind(summary_cells[,1],rep("raw",each = length(summary_cells[,1])))),

as.data.frame(cbind(summary_cells[,2],rep("clean",each = length(summary_cells[,2])))))

counts$sample <- rep(rownames(summary_cells),times =2)

colnames(counts)<- c("count","Stat","sample")

counts[,1] <- as.numeric(counts[,1])

counts$Stat <- factor(counts$Stat, levels=c("raw", "clean"), ordered=TRUE)

fit_cell_count <- ggplot(data =counts, mapping = aes(x = sample, y=count))+

geom_bar(aes(fill = Stat),stat = 'identity', position = 'dodge') + scale_fill_brewer(palette = "Set1") +

theme(text=element_text(size=10),legend.title=element_blank(),

panel.background = element_rect(fill = "white", colour = "black",size = 0.2),

legend.key = element_rect(fill = "white", colour = "white"),

legend.background = (element_rect(colour= "white",fill = "white")))

fit_cell_count

savePDF(filename = 'PDFs/fit_cell_count.pdf',plot = fit_cell_count,width = 9,height = 9)

#质控后的小提琴图

violin_after=list()

for (i in 1:length(datalist)){

violin_after[[i]] <- VlnPlot(datalist[[i]],

features = c("nFeature_RNA", "nCount_RNA", "percent.mt","percent.Ribo"),

pt.size = 0.1,

ncol = 4)

}

pearplot_after <- CombinePlots(plots = violin_after , nrow=length(datalist), legend="none")

pearplot_after

savePDF(filename = 'PDFs/QC_after.pdf',plot = pearplot_after,he=15,wi=15)

save(datalist,file = 'origin_datas/scRNA/datalist.RData')

rm(datalist)

#3、数据预处理####

#执行标准预处理（log-normalization）并基于方差稳定化转换（“vst”）识别变量特征，接下来对集成数据进行归一化、运行PCA并使用UMAP可视化结果

# Normalizing the data

load('origin_datas/scRNA/datalist.RData')

sce <- merge(datalist[[1]],y=datalist[2:length(datalist)])

sce <- NormalizeData(sce, normalization.method = "LogNormalize", scale.factor = 10000)

sce <- FindVariableFeatures(sce,

selection.method = "vst",

nfeatures = 2000,

mean.cutoff=c(0.0125,3),

dispersion.cutoff =c(1.5,Inf))

### 可视化前20个高变基因

top20 <- head(VariableFeatures(sce), 20)

plot1 <- VariableFeaturePlot(sce)

plot2 <- LabelPoints(plot = plot1, points = top20, repel = TRUE, size=3.0)

feat_20 <- CombinePlots(plots = list(plot1, plot2),legend="bottom")

feat_20

savePDF(filename = 'PDFs/feat_20.pdf',plot = feat_20,he=10,wi=15)

#ScaleData

scale.genes <- rownames(sce)

sce <- ScaleData(sce, features = scale.genes)

#样本的分组

meta1<-data.frame(matrix(nrow=length(sce@meta.data$orig.ident), ncol=3))

colnames(meta1)=c('Sample','Group1','Group2')

meta1$Sample=sce@meta.data$orig.ident

unique(meta1$Sample)

### Group1 IPMN为导管内乳头状黏液性肿瘤；PASC:胰腺腺鳞癌 ；Normal：健康胰腺

meta1[grep("GSM5032771",meta1$Sample),]$Group1="IPMN"

meta1[grep("GSM5032772",meta1$Sample),]$Group1="PASC"

meta1[grep("GSM5032773",meta1$Sample),]$Group1="Normal"

### Group2 Turmal:肿瘤样本，Normal为正常

meta1[grep("GSM5032771",meta1$Sample),]$Group2="Turmal"

meta1[grep("GSM5032772",meta1$Sample),]$Group2="Turmal"

meta1[grep("GSM5032773",meta1$Sample),]$Group2="Normal"

sce <- AddMetaData(sce, meta1$Sample,col.name = "Sample")

sce <- AddMetaData(sce, meta1$Group1,col.name = "Group1")

sce <- AddMetaData(sce, meta1$Group2,col.name = "Group2")

#PCA降维，选择合适的拐点

sce <- RunPCA(sce, features = VariableFeatures(sce))

dimplot1 <- DimPlot(sce, reduction = "pca")

elbowplot1 <- ElbowPlot(sce, ndims=50, reduction="pca")

sc_pca <- dimplot1+elbowplot1

sc_pca

savePDF(filename = 'PDFs/sc_pca.pdf',plot = sc_pca,he=10,wi=15)

DimPlot(sce, reduction = "pca",split.by = 'Sample')

Dims <- 40

Resolution <- 0.5

sce <- FindNeighbors(object = sce, dims = 1:Dims)

sce <- FindClusters(object = sce, resolution = Resolution)

#颜色

allcolour=c("#DC143C","#0000FF","#20B2AA","#FFA500","#9370DB","#98FB98","#F08080","#1E90FF","#7CFC00","#FFFF00",

"#808000","#FF00FF","#CCCCFF","#000000","#7B68EE","#9400D3","#A0522D","#800080","#D2B48C","#D2691E",

"#87CEEB","#40E0D0","#5F9EA0","#FF1493","#0000CD","#008B8B","#FFE4B5","#8A2BE2","#228B22","#E9967A",

"#4682B4","#32CD32","#F0E68C","#FFFFE0","#EE82EE","#FF6347","#6A5ACD","#9932CC","#8B008B","#8B4513",

"#DEB887")

length(table(sce@active.ident))

mycolor = allcolour[1:length(table(sce@active.ident))]

#### 按cluster进行占比统计

cluster.frequency.table <- sce@meta.data %>%

dplyr::count(seurat_clusters) %>%

dplyr::mutate(freq = n / sum(n)*100) %>%

ungroup()%>%as.data.frame()

cluster.frequency.table

pie(cluster.frequency.table$n, labels=round(cluster.frequency.table$freq,2),radius=1.0, main = "Percentage of Cluster", col=mycolor)

legend("right",legend=unique(cluster.frequency.table$seurat_clusters),bty="n",fill=mycolor)

#统计每一个样本，每一个亚群所占的比例

cluster.frequency.sample=data.frame()

for (i in as.character(unique(sce@meta.data$Sample))){

data1<-sce@meta.data[which(sce@meta.data$Sample==i),]

dat1 <- data1 %>%

dplyr::group_by(Sample) %>%

dplyr::count(seurat_clusters) %>%

dplyr::mutate(freq = n / sum(n)*100) %>%

ungroup()%>%as.data.frame()

cluster.frequency.sample=rbind(cluster.frequency.sample,dat1)

}

cluster.freq.sample<-tidyr::spread(data=cluster.frequency.sample[,c("Sample","seurat_clusters","freq")],

key=Sample, value=freq)

cluster.freq.sample[is.na(cluster.freq.sample)]<-0

head(cluster.freq.sample)

#rownames(cluster.freq.sample)=cluster.freq.sample$seurat_clusters

#从内圈到外圈依次是GSM5032771，GSM5032772，GSM5032773

cluster.freq<-ggplot(data=cluster.frequency.sample, mapping=aes(x=Sample,y=freq,fill=seurat_clusters))+

geom_bar(stat='identity',width=0.9)+coord_polar(theta="y",start = 0)+

theme_bw() +

theme(panel.border = element_blank(),

panel.grid.major = element_blank(),

panel.grid.minor = element_blank(),

axis.line = element_blank())+

scale_fill_manual(values=mycolor)

cluster.freq

pdf('PDFs/cluster_freq.pdf',he=7,wi=9)

cluster.freq

dev.off()

write.csv(cluster.frequency.sample,file ="files/cluster.frequency.csv")

#4、降维#####

### UMAP

sce <- RunUMAP(sce, dims=1:Dims, reduction="pca")

### Umap plot

sc_umap = DimPlot(sce,cols=mycolor,

reduction="umap",

label = "T", pt.size = 0.2,

label.size = 4) +

theme(axis.line = element_blank(),

axis.text = element_blank(),

axis.ticks = element_blank(),

axis.title = element_blank())

sc_umap

umap_embeddings <- Embeddings(sce,reduction = "umap")

head(umap_embeddings)

###tsne 降维

sce <- RunTSNE(sce,

dims=1:Dims,

reduction="pca",

perplexity=30,

max_iter=1000)

#5、marker gene的识别####

#寻找差异基因时的差异倍数

Logfc = 0.5

#差异基因时最小的表达比例

Minpct = 0.1

DefaultAssay(sce) <- "RNA"

sce.markers <- FindAllMarkers(object = sce,logfc.threshold = Logfc, min.pct = Minpct,only.pos = T)

sce.markers["pct.diff"]=sce.markers$pct.1-sce.markers$pct.2

sce.markers <- sce.markers[sce.markers$p_val_adj<0.05,]

length(unique(sce.markers$gene))

head(sce.markers)

write.table(sce.markers,'results/files/scRNA_marker_gene.txt',quote = F,row.names = F,sep='\t')

### 选择前5个marker基因

Top5 <- sce.markers %>% group_by(cluster) %>% slice_max(n =5, order_by = avg_logFC)

Top5 <- intersect(unique(Top5$gene),rownames(sce@assays$RNA@meta.features))

sc_marker_dotplot <- DotPlot(object = sce, features = Top5,cols=c("blue", "red"),scale = T)+

RotatedAxis()+ ggtitle("Top 5 Marker Genes")+

theme(plot.title = element_text(hjust = 0.5))

sc_marker_dotplot

#热图展示

library(viridisLite)

sc_marker_heatmap<- DoHeatmap(object = sce,

features = Top5,

group.colors = mycolor,

label = F) + ggtitle("Top 5 Marker Genes") +

theme(plot.title = element_text(hjust = 0.5))

#+scale_fill_gradientn(colors = viridis(10))

sc_marker_heatmap

savePDF(filename = 'PDFs/Fig2.pdf',plot = sc_marker_heatmap,width = 12,height = 12)

#

#5、细胞类型鉴定####

#

#data <- GetAssayData(sce, assay = 'RNA', slot = 'counts')

#cell_metadata <- sce@meta.data

#gene_annotation <- data.frame(gene_short_name = rownames(data))

#rownames(gene_annotation) <- rownames(data)

#save(data,cell_metadata,gene_annotation,file = 'gatnett.RData')

get_cell_mark <- function(tissu){

data=read.delim('/pub1/data/mg_projects/users/wangtl/public/human.cellmark.txt',sep='\t',header = T)

#tissu

if (length(tissu)==1){

cell_mark=data[which(data$tissueType==tissu),]

}else if(length(tissu>1)){

cell_mark=data.frame()

for (i in tissu){

cell_mark_temp=data[which(data$tissueType==i),]

cell_mark=rbind(cell_mark,cell_mark_temp)

}

}

#dim(cell_mark)

cell_type=as.character(unique(cell_mark$cellName))

#class(cell_mark)

cell_mark1 <- cell_mark[, c("cellName","geneSymbol","PMID")]

gene_symbol=read.delim('/pub1/data/mg_projects/users/wangtl/public/GeneTag.genecode.v32.txt',sep='\t',header = T)

gene_symbol=as.character(gene_symbol[which(gene_symbol$TYPE=='protein_coding'),]$SYMBOL)

if(file.exists('cell_mark_select.txt')){

file.remove('cell_mark_select.txt')

}

for (i in cell_type){

cell_mark2=cell_mark1[which(cell_mark1$cellName==i),]

gene=as.character(unique(unlist(stringr::str_split(cell_mark2$geneSymbol,', '))))

gene=intersect(gene_symbol,gene)

if (length(gene)>0){

write.table(paste0('>',i),file = paste0('cell_mark_select.txt'),row.names = F,col.names = F,append = T,quote = F)

genes=paste0('expressed: ',paste(gene,collapse = ', '))

write.table(genes,file = paste0('cell_mark_select.txt'),row.names = F,col.names = F,append = T,quote = F)

}

}

}

#修改组织类型

tissu=c('Pancreas','Pancreatic acinar tissue','Fetal pancreas','Peripheral blood','Blood')

get_cell_mark(tissu = tissu)

# mg_anno_garnett_cell <- function(cds){

# command=paste0('R_LIBS_SITE=" " R_LIBS_USER=" " /home/wangtl/miniconda/envs/R4/bin/Rscript ','/pub1/data/mg_projects/users/wangtl/public/cell_anno_garnett.R ',cds)

# print(paste0('garnett_cell CMD:',command))

# grep_out<-system(command, intern = F)

# cat(grep_out)

# }

#cds='/pub1/data/mg_projects/users/wangtl/202107/20210804_osteoporosis_scRNA/gatnett.RData'

#mg_anno_garnett_cell(cds)

#cell_anno=read.delim('cell_anno.txt',sep='\t',header = T,row.names = 1)

#第二种

#/home/wangtl/miniconda/envs/R4/bin/Rscript /pub1/data/mg_projects/users/wangtl/public/cell_anno_garnett.R /pub1/data/mg_projects/users/wangtl/202107/20210804_osteoporosis_scRNA/gatnett.RData

#

# sce <- AddMetaData(sce, metadata = cell_anno)

#

# head(as.data.frame(sce$seurat_clusters))

# cell_name=rownames(data.frame(sce$seurat_clusters))

# cell_cluster_anno=data.frame(cell=cell_name,

# seurat_clusters=data.frame(sce$seurat_clusters)[cell_name,1],

# cell_type=data.frame(sce$cell_type)[cell_name,1],

# cluster_ext_type=data.frame(sce$cluster_ext_type)[cell_name,1])

# head(cell_cluster_anno)

# table(cell_cluster_anno$seurat_clusters,cell_cluster_anno$cell_type)

#

# cluter_anno <- function(data=cell_cluster_anno[,c('seurat_clusters','cell_type')]){

# colnames(data)=c('seurat_clusters','cell_type')

# data=data.frame(table(data$seurat_clusters,data$cell_type))

# colnames(data)=c('seurat_clusters','cell_type','cell_num')

# data=data[which(data$cell_type!='Unknown'),]

# cell_type=as.character(unique(data$cell_type))

# data1=aggregate(data$cell_num, by=list(cell_type=data$cell_type),sum)

# colnames(data1)=c('cell_type','sum')

# data=merge(data,data1,by='cell_type')

# data$freq=data$cell_num/data$sum

# data1=data.frame()

# for (i in as.character(unique(data$seurat_clusters))){

# dat=data[which(data$seurat_clusters==i),]

# dat=dat[which(dat$freq==max(dat$freq)),]

# }

# }

#

# cluter_anno_res=cluter_anno(data=cell_cluster_anno[,c('seurat_clusters','cell_type')])

# cluter_anno_res

scRNA_cell_anno <- function(sce.markers,tissu,pvalueCutoff = 0.05){

data=read.delim('/pub1/data/mg_projects/users/wangtl/public/human.cellmark.txt',sep='\t',header = T)

#tissu

if (length(tissu)==1){

cell_mark=as.data.frame(data[which(data$tissueType==tissu),])

}else if(length(tissu>1)){

cell_mark=data.frame()

for (i in tissu){

cell_mark_temp=data[which(data$tissueType==i),]

cell_mark=rbind(cell_mark,cell_mark_temp)

}

}

gene_symbol=read.delim('/pub1/data/mg_projects/users/wangtl/public/GeneTag.genecode.v32.txt',sep='\t',header = T)

gene_symbol=as.character(gene_symbol[which(gene_symbol$TYPE=='protein_coding'),]$SYMBOL)

#dim(cell_mark)

cell_type=as.character(unique(cell_mark$cellName))

#class(cell_mark)

cell_mark1 <- cell_mark[, c("cellName","geneSymbol")]

dat=data.frame()

for (i in cell_type){

cell_mark2=cell_mark1[which(cell_mark1$cellName==i),]

gene=as.character(unique(unlist(stringr::str_split(cell_mark2$geneSymbol,', '))))

gene=intersect(gene_symbol,gene)

if (length(gene)>0){

dat1=data.frame(cell_name=i,marker=gene)

dat=rbind(dat,dat1)

}

}

dat=na.omit(dat)

dat=dat[which(dat$marker!='NA'),]

sce.markers=intersect(sce.markers,gene_symbol)

cell_anno=clusterProfiler::enricher(sce.markers, TERM2GENE=dat, minGSSize=1, maxGSSize = 5000,pvalueCutoff = pvalueCutoff)

return(cell_anno)

}

cell_anno=data.frame()

for (i in unique(as.character(sce.markers$cluster))){

print(i)

genes=as.character(unique(sce.markers[which(sce.markers$cluster==i),]$gene))

cell_anno1=scRNA_cell_anno(sce.markers =genes,tissu = tissu,pvalueCutoff = 0.05)

#head(cell_anno1@result)

cell_anno1=cell_anno1@result

#cell_type=as.character(cell_anno1[which(cell_anno1$p.adjust==min(cell_anno1$p.adjust)),]$ID)

cell_type=as.character(cell_anno1[order(-cell_anno1$p.adjust, cell_anno1$GeneRatio),]$ID)[nrow(cell_anno1)]

cell_anno=rbind(cell_anno, data.frame(cell_type=cell_type,seraut_cluster=i))

}

cell_anno

write.table(cell_anno,'files/cell_anno.txt',quote = F,sep='\t',row.names = F)

table(cell_anno$cell_type)

##MST降维查看是否要进行合并亚群

Top200 <- sce.markers %>% group_by(cluster) %>% slice_max(n =500, order_by = avg_logFC) %>% slice_max(n =200, order_by =pct.diff)

dat<-as.matrix(GetAssayData(sce,slot = "counts"))

dat=dat[unique(Top200$gene),]

sce_cell_type<-sce@meta.data

dat=merge(data.frame(cell=rownames(sce@meta.data),

seurat_clusters=sce@meta.data$seurat_clusters),

data.frame(cell=colnames(dat),t(dat)),

by='cell')

dat[1:4,1:4]

#按照seurat_clusters取平均表达值

dat=dat[,-1]

dat=aggregate(.~seurat_clusters,dat,mean)

rownames(dat)=dat$seurat_clusters

dat=dat[,-1]

mst_dis_plot<-function(dat){

library(igraph)

mst_adjacency <- as.matrix(stats::dist(dat, method = "euclidean"))

fullGraph <- igraph::graph.adjacency(mst_adjacency,

mode = "undirected",

weighted = TRUE)

mst_data=igraph::minimum.spanning.tree(fullGraph)

gg=igraph::layout_with_gem(mst_data)

E(mst_data)$color='black'

par(pin = c(5,5))

plot(mst_data, layout=gg, vertex.size=10,

vertex.label.cex=2,

vertex.label.color= "black",

vertex.frame.color='white')

}

mst_dis_plot(dat)

pdf('PDFs/MST.pdf',he=9,wi=9)

mst_dis_plot(dat)

dev.off()

table(cell_anno$cell_type)

#B cell 4,10

cell_anno[which(cell_anno$cell_type=='B cell'),]

# c4_10 <- FindAllMarkers(object = subset(sce, idents = c("4", "10")),

# logfc.threshold = Logfc, min.pct = Minpct,only.pos = T)

# c4_10_top5=c4_10 %>% group_by(cluster) %>% slice_max(n =100, order_by = avg_logFC) %>% data.frame()

c4_10=sce.markers[which(sce.markers$cluster=='4'),]

c4_10=rbind(c4_10,sce.markers[which(sce.markers$cluster=='10'),])

c4_10_top5=c4_10 %>% group_by(cluster) %>% slice_max(n =50, order_by = avg_logFC) %>% data.frame()

#TNFRSF13C

#CCR7 ACAP1 TCL1A STMN1 MKI67 RGS13

VlnPlot(object = subset(sce, idents = c("4", "10")),

features = c4_10_top5$gene[100:109],

pt.size = 0)

VlnPlot(object = subset(sce, idents = c("4", "10")),

features = c('TNFRSF13C','CCR7', 'ACAP1', 'TCL1A', 'STMN1', 'MKI67', 'RGS13' ),

pt.size = 0)

#Cancer cell 9,15,18

cell_anno[which(cell_anno$cell_type=='Cancer cell'),]

c9_15_18=sce.markers[which(sce.markers$cluster=='9'),]

c9_15_18=rbind(c9_15_18,sce.markers[which(sce.markers$cluster=='15'),])

c9_15_18=rbind(c9_15_18,sce.markers[which(sce.markers$cluster=='18'),])

c9_15_18_top5=c9_15_18 %>% group_by(cluster) %>% slice_max(n =50, order_by = avg_logFC) %>% data.frame()

#DEFB1 TFF1 CRISP3 CLDN3 CEACAM6 WFDC2 MUC5B SLC3A1 TSPAN8 EDN1 GPX2

#CELA3A CPB1 PNLIPRP2 GP2 SYCN CELA2A CELA3B ALB CUZD1 SERPINI2 AC009078.2 KLK1 PDIA2 ERP27 MT1G IGFBP2

#LINC01681 SPATA22 CMC2 IER3-AS1 SCGB3A1 AC093484.3 RGS5 AL138963.3 TIMP3 HSPB6 C7 VIM-AS1 AC243829.4 AC011603.2 ICK AC010761.1 LPL

VlnPlot(object = subset(sce, idents = c("9", "15","18")),

features = c9_15_18_top5$gene[140:150],

pt.size = 0)

#CD1C-CD141- dendritic cell 0,1

cell_anno[which(cell_anno$cell_type=='CD1C-CD141- dendritic cell'),]

c0_1=sce.markers[which(sce.markers$cluster=='0'),]

c0_1=rbind(c0_1,sce.markers[which(sce.markers$cluster=='1'),])

c0_1_top5=c0_1 %>% group_by(cluster) %>% slice_max(n =50, order_by = avg_logFC) %>% data.frame()

#ADAMDEC1 CLEC4E KCNMA1 SELENOP PLTP CH25H SLC1A3 NUPR1 RNASE1

#MMP12 MRC1 RETN IL1RN IFI6 ALOX5AP VSIG4 FBP1 MARCO CXCL1 ISG15

VlnPlot(object = subset(sce, idents = c("0", "1")),

features = c0_1_top5$gene[100],

pt.size = 0)

#CD1C+_B dendritic cell 6,7,16

cell_anno[which(cell_anno$cell_type=='CD1C+_B dendritic cell'),]

c6_7_16=sce.markers[sce.markers$cluster %in% c('6','7','16'),]

c6_7_16_top5=c6_7_16 %>% group_by(cluster) %>% slice_max(n =50, order_by = avg_logFC) %>% data.frame()

#KRT5 CALB1 KRT16 S100P SERPINB3 DSP LAMA3 SAA1 MIR205HG LAMC2 S100A16 COL17A1 AQP3 CP FAM83A CAV1 S100A14 KRT7 KRT6B MUC1 SDC1 GJB2 AGR2

#IL1R2 LGALS2 LGALS2

#S100B CXCL10 PCLAF TYMS RETN CLEC5A HMMR CDK1 TNF FAM111A TK1 SMC2

VlnPlot(object = subset(sce, idents = c("6", "7","16")),

features = c6_7_16_top5$gene[140:150],

pt.size = 0)

#Fibroblast 2,11

cell_anno[which(cell_anno$cell_type=='Fibroblast'),]

c2_11=sce.markers[sce.markers$cluster %in% c('2','11'),]

c2_11_top5=c2_11 %>% group_by(cluster) %>% slice_max(n =50, order_by = avg_logFC) %>% data.frame()

#CXCL13 GAS1 CHI3L2 HILPDA

#C7 CXCL14 IGFBP5 HSPB6 CCL2

VlnPlot(object = subset(sce, idents = c("2", "11")),

features = c2_11_top5$gene[90:100],

pt.size = 0)

#Natural killer cell 5,14

cell_anno[which(cell_anno$cell_type=='Natural killer cell'),]

c5_14=sce.markers[sce.markers$cluster %in% c('5','14'),]

c5_14_top5=c5_14 %>% group_by(cluster) %>% slice_max(n =50, order_by = avg_logFC) %>% data.frame()

#GNLY IL7R XCL1 KLRB1 GZMB MAF SPOCK2 TSPYL2

#CRTAM TIGIT CTSW LINC01871 PYHIN1 KLRD1 GNLY GZMA MKI67 TOP2A HIST1H1E STMN1 CENPF HIST1H1D NUSAP1 ASPM TMPO HIST1H1C PCLAF TYMS TUBB CKS1B ATAD2 HIST1H1B NUF2 TRDC CENPE ITM2C KLRD1 DNAJC9 TNFRSF18 PRF1

VlnPlot(object = subset(sce, idents = c("5", "14")),

features = c5_14_top5$gene[90:100],

pt.size = 0)

cell_anno1=cell_anno

cell_anno1

#B cell 4,10

cell_anno1[5,1]=paste0(cell_anno1[5,1],'_1')

cell_anno1[11,1]=paste0(cell_anno1[11,1],'_2')

#Cancer cell 9,15,18

cell_anno1[10,1]=paste0(cell_anno1[10,1],'_1')

cell_anno1[16,1]=paste0(cell_anno1[16,1],'_2')

cell_anno1[19,1]=paste0(cell_anno1[19,1],'_3')

#CD1C-CD141- dendritic cell 0,1

cell_anno1[1,1]=paste0(cell_anno1[1,1],'_1')

cell_anno1[2,1]=paste0(cell_anno1[2,1],'_2')

#CD1C+_B dendritic cell 6,7,16

cell_anno1[7,1]=paste0(cell_anno1[7,1],'_1')

cell_anno1[8,1]=paste0(cell_anno1[8,1],'_2')

cell_anno1[17,1]=paste0(cell_anno1[17,1],'_3')

#Fibroblast 2,11

cell_anno1[3,1]=paste0(cell_anno1[3,1],'_1')

cell_anno1[12,1]=paste0(cell_anno1[12,1],'_2')

#Natural killer cell 5,14

cell_anno1[6,1]=paste0(cell_anno1[6,1],'_1')

cell_anno1[15,1]=paste0(cell_anno1[15,1],'_2')

table(cell_anno1$cell_type)

#保存

save(sce,file = 'sce1.RData')

## 手动更改细胞类型

sce <- RenameIdents(object = sce,

"0" = as.character(cell_anno1[1,1]),

"1" = as.character(cell_anno1[2,1]),

"2" = as.character(cell_anno1[3,1]),

"3" = as.character(cell_anno1[4,1]),

"4" = as.character(cell_anno1[5,1]),

"5" = as.character(cell_anno1[6,1]),

"6" = as.character(cell_anno1[7,1]),

"7" = as.character(cell_anno1[8,1]),

"8" = as.character(cell_anno1[9,1]),

"9" = as.character(cell_anno1[10,1]),

"10" = as.character(cell_anno1[11,1]),

"11" = as.character(cell_anno1[12,1]),

"12" = as.character(cell_anno1[13,1]),

"13" = as.character(cell_anno1[14,1]),

"14" = as.character(cell_anno1[15,1]),

"15" = as.character(cell_anno1[16,1]),

"16" = as.character(cell_anno1[17,1]),

"17" = as.character(cell_anno1[18,1]),

"18" = as.character(cell_anno1[19,1]),

"19" = as.character(cell_anno1[20,1])

)

save(sce,file = 'sce2.RData')

#6、拟时序分析####

library(monocle)

#选择做拟时序的亚群

# Mono_tj<-subset(seurat.object, idents = c(1,2,4,6,7))

exp.rawdata <- as(as.matrix(GetAssayData(sce,slot = "counts")), 'sparseMatrix')

#构建featuredata，一般featuredata需要两个col，一个是gene_id,一个是gene_short_name,row对应counts的rownames

feature_ann<-data.frame(gene_short_name=rownames(sce))

rownames(feature_ann) <- rownames(exp.rawdata)

#

scRNA_fd <-new("AnnotatedDataFrame", data = feature_ann)

#

#Seurat object中的@meta.data一般会存放表型相关的信息如cluster、sample的来源、group等，所以选择将metadata转换为phenodata

sample_ann <- sce@meta.data

#rownames(sample_ann)<-colnames(Mono_matrix)

scRNA_pd<-new("AnnotatedDataFrame", data =sample_ann)

#build new cell data set

scRNA.cds<-newCellDataSet(exp.rawdata,

phenoData =scRNA_pd,

featureData =scRNA_fd,

expressionFamily=negbinomial.size())

#查看phenodata、featuredata

head(pData(scRNA.cds))

head(fData(scRNA.cds))

#计算size factors 和 dispersions，用于后期分析；

scRNA.cds <- estimateSizeFactors(scRNA.cds)

scRNA.cds <- estimateDispersions(scRNA.cds)

# Filtering low-quality cells

scRNA.cds <- detectGenes(scRNA.cds, min_expr = 0.1)

expressed_genes <- row.names(subset(fData(scRNA.cds),

num_cells_expressed >= 0.01*ncol(scRNA.cds)))

#筛选基因,这里可以根据自己的需要筛选特定的基因

ordering_genes <- intersect(unique(Top200$gene),rownames(scRNA.cds))

scRNA.cds <- setOrderingFilter(scRNA.cds, ordering_genes)

plot_ordering_genes(scRNA.cds)

#用DDRtree 进行降维分析

scRNA.cds <- reduceDimension(scRNA.cds,

norm_method ='log',

max_components = 2,

method = 'DDRTree')

#计算psudotime值

scRNA.cds <- orderCells(scRNA.cds)

head(pData(scRNA.cds))

scRNA.cds$cell_type=scRNA.cds$seurat_clusters

scRNA.cds$cell_type=gsub('^0$',as.character(cell_anno1$cell_type[1]),scRNA.cds$cell_type)

scRNA.cds$cell_type=gsub('^1$',as.character(cell_anno1$cell_type[2]),scRNA.cds$cell_type)

scRNA.cds$cell_type=gsub('^2$',as.character(cell_anno1$cell_type[3]),scRNA.cds$cell_type)

scRNA.cds$cell_type=gsub('^3$',as.character(cell_anno1$cell_type[4]),scRNA.cds$cell_type)

scRNA.cds$cell_type=gsub('^4$',as.character(cell_anno1$cell_type[5]),scRNA.cds$cell_type)

scRNA.cds$cell_type=gsub('^5$',as.character(cell_anno1$cell_type[6]),scRNA.cds$cell_type)

scRNA.cds$cell_type=gsub('^6$',as.character(cell_anno1$cell_type[7]),scRNA.cds$cell_type)

scRNA.cds$cell_type=gsub('^7$',as.character(cell_anno1$cell_type[8]),scRNA.cds$cell_type)

scRNA.cds$cell_type=gsub('^8$',as.character(cell_anno1$cell_type[9]),scRNA.cds$cell_type)

scRNA.cds$cell_type=gsub('^9$',as.character(cell_anno1$cell_type[10]),scRNA.cds$cell_type)

scRNA.cds$cell_type=gsub('^10$',as.character(cell_anno1$cell_type[11]),scRNA.cds$cell_type)

scRNA.cds$cell_type=gsub('^11$',as.character(cell_anno1$cell_type[12]),scRNA.cds$cell_type)

scRNA.cds$cell_type=gsub('^12$',as.character(cell_anno1$cell_type[13]),scRNA.cds$cell_type)

scRNA.cds$cell_type=gsub('^13$',as.character(cell_anno1$cell_type[14]),scRNA.cds$cell_type)

scRNA.cds$cell_type=gsub('^14$',as.character(cell_anno1$cell_type[15]),scRNA.cds$cell_type)

scRNA.cds$cell_type=gsub('^15$',as.character(cell_anno1$cell_type[16]),scRNA.cds$cell_type)

scRNA.cds$cell_type=gsub('^16$',as.character(cell_anno1$cell_type[17]),scRNA.cds$cell_type)

scRNA.cds$cell_type=gsub('^17$',as.character(cell_anno1$cell_type[18]),scRNA.cds$cell_type)

scRNA.cds$cell_type=gsub('^18$',as.character(cell_anno1$cell_type[19]),scRNA.cds$cell_type)

scRNA.cds$cell_type=gsub('^19$',as.character(cell_anno1$cell_type[20]),scRNA.cds$cell_type)

class(scRNA.cds$seurat_clusters)

scRNA.cds$cell_type=as.factor(scRNA.cds$cell_type)

scRNA.cds$seurat_clusters=as.factor(scRNA.cds$seurat_clusters)

plot_cell_trajectory(scRNA.cds,cell_size = 1, color_by = "cell_type")+

scale_color_manual(breaks = as.character(cell_anno1$cell_type), values=mycolor) +

theme(legend.position = "right")+facet_wrap("~Group2", nrow = 1)

mono_cell=plot_cell_trajectory(scRNA.cds,cell_size = 1, color_by = "seurat_clusters")+

scale_color_manual(breaks = as.character(cell_anno1$seraut_cluster), values=mycolor) +

theme(legend.position = "right",

text=element_text(size=10),

legend.title=element_blank(),

panel.background = element_rect(fill = "white", colour = "black",size = 0.2),

legend.key = element_rect(fill = "white", colour = "white"),

legend.background = (element_rect(colour= "white",fill = "white")))+

facet_wrap("~Group2", nrow = 1)+

guides(shape = guide_legend(override.aes = list(size = 3),nrow = 10),

color = guide_legend(override.aes = list(size = 3),nrow = 10))

mono_cell

mono_state<-plot_cell_trajectory(scRNA.cds,cell_size = 1, color_by = "State")+

#scale_color_manual(breaks = as.character(cell_anno1$cell_type), values=mycolor) +

theme(legend.position = "right",

text=element_text(size=10),

panel.background = element_rect(fill = "white", colour = "black",size = 0.2),

legend.key = element_rect(fill = "white", colour = "white"),

legend.background = (element_rect(colour= "white",fill = "white")))+

facet_wrap("~Group2", nrow = 1)

mono_state

mono_time=plot_cell_trajectory(scRNA.cds,cell_size = 1, color_by = "Pseudotime")+

scale_color_gradient(low = 'red',high = 'blue')+

theme(legend.position = "right",

text=element_text(size=10),

panel.background = element_rect(fill = "white", colour = "black",size = 0.2),

legend.key = element_rect(fill = "white", colour = "white"),

legend.background = (element_rect(colour= "white",fill = "white")))+

facet_wrap("~Group2", nrow = 1)

mono_time

mono_merge<-mg_merge_plot(mono_time,mono_cell,mono_state,labels = c('A','B','C'),nrow=3,ncol=1)

savePDF(filename = 'PDFs/Fig3.pdf',plot = mono_merge,width = 9,height = 12)

#savePDF(filename = 'PDFs/Fig3B.pdf',plot = mono_time,width = 7,height = 7)

rm(sce)

#BEAM统计分析

BEAM_res1 <- BEAM(scRNA.cds[ordering_genes,], branch_point = 1, cores = 1)

BEAM_res1 <- BEAM_res1[order(BEAM_res1$qval),]

BEAM_res1 <- BEAM_res1[,c("gene_short_name", "pval", "qval")]

#选100前个基因可视化

BEAM_genes1 <- top_n(BEAM_res1, n = 100, desc(qval)) %>% pull(gene_short_name) %>% as.character()

length(BEAM_genes1)

BEAM_p1 <- plot_genes_branched_heatmap(scRNA.cds[BEAM_genes1,], branch_point = 1,

num_clusters = 3, show_rownames = T, return_heatmap = T)

BEAM_p1$ph_res

savePDF(filename = 'PDFs/Fig3D.pdf',plot = BEAM_p1$ph_res,width = 7,height = 12)

gene_clust<-BEAM_p1$annotation_row

BEAM_genes_dat<-t(as.matrix(scRNA.cds@assayData$exprs[BEAM_genes1,]))

meta_clust<-data.frame(cell=colnames(scRNA.cds),

State=scRNA.cds$State)

BEAM_genes_dat<-merge(data.frame(cell=rownames(BEAM_genes_dat),BEAM_genes_dat),

meta_clust,by='cell')

BEAM_genes_dat[1:4,1:4]

BEAM_genes_dat=BEAM_genes_dat[,-1]

BEAM_genes_dat$State=as.factor(BEAM_genes_dat$State)

BEAM_genes_dat=aggregate(.~State,BEAM_genes_dat,mean)

BEAM_genes_dat[,1:4]

rownames(BEAM_genes_dat)=BEAM_genes_dat$State

BEAM_genes_dat=BEAM_genes_dat[,-1]

BEAM_genes_dat=t(BEAM_genes_dat)

head(BEAM_genes_dat)

rownames(BEAM_genes_dat)=gsub('\\.','-',rownames(BEAM_genes_dat))

bk<-c(seq(-2,-0.1,by=0.01),seq(0,2,by=0.01))

gene_clust=data.frame(gene=rownames(gene_clust),Cluster=gene_clust$Cluster)

gene_clust=gene_clust[order(gene_clust$Cluster),]

rownames(gene_clust)=gene_clust$gene

head(gene_clust)

write.table(gene_clust,'results/files/state_127_gene.txt',quote = F,row.names = F,sep='\t')

meta_clust1<-data.frame(cell=colnames(scRNA.cds),

State=scRNA.cds$State,

seurat_clusters=scRNA.cds$seurat_clusters)

tongji_state_seraut<-as.data.frame(table(meta_clust1$State,meta_clust1$seurat_clusters))

tongji_state_seraut=spread(tongji_state_seraut,key = Var2,value = Freq)

write.table(tongji_state_seraut,'files/tongji_state_seraut.txt',sep='\t',quote = F,row.names = F)

gene_clust1=data.frame(Cluster=gene_clust$Cluster)

rownames(gene_clust1)=gene_clust$gene

pdf('PDFs/state.gene.pdf',he=15,wi=7)

pheatmap::pheatmap(log2(BEAM_genes_dat[rownames(gene_clust1),]),scale = 'row',

show_colnames = T,annotation_row = gene_clust1,

show_rownames = T,cluster_rows = F,cluster_cols = F,

#color = colorRampPalette(c("navy", "white", "firebrick3"))(50),

color=c(colorRampPalette(colors=c("blue","white"))(length(bk)/2),

colorRampPalette(colors=c("white","red"))(length(bk)/2)),

legend_breaks=seq(-2,2,1),breaks=bk)

dev.off()

save(scRNA.cds,file = 'scRNA.cds.RData')

rm(scRNA.cds)

####ReactomeGSA 富集分析########

library(ReactomeGSA)

load('sce1.RData')

DefaultAssay(sce) <- "RNA"

gsva_result <- analyse_sc_clusters(sce, verbose = TRUE)

pathway_expression <- pathways(gsva_result)

colnames(pathway_expression) <- gsub("\\.Seurat", "", colnames(pathway_expression))

write.csv(pathway_expression,file = 'results/files/reactomegsa_sce.csv',quote = F,row.names = F)

# find the maximum differently expressed pathway

max_difference <- do.call(rbind, apply(pathway_expression, 1, function(row) {

values <- as.numeric(row[2:length(row)])

return(data.frame(name = row[1], min = min(values), max = max(values)))

}))

max_difference$diff <- max_difference$max - max_difference$min

max_difference <- max_difference[order(max_difference$diff, decreasing = T), ]

write.csv(max_difference,"files/max_difference.csv")

plot_num = 20

plot_gsva <- pathway_expression[rownames(max_difference[1:plot_num,]),]

pdf("PDFs/max_difference_top20.pdf")

pheatmap::pheatmap(t(plot_gsva[,-1]),

scale ="none",

angle_col = 90,cellwidth = 15,cellheight = 15,

labels_col = plot_gsva[,1],

# cellwidth = 10, cellheight = 8,

color = colorRampPalette(c("navy", "white", "firebrick3"))(50))

dev.off()

library(tidyverse)

pp=list()

for (i in 1:plot_num){

barplot_pathway <- plot_gsva_pathway(gsva_result, pathway_id = rownames(max_difference)[i])

barplot_pathway$data %>% mutate(absmy = ifelse(expr>=0, "Z","Fy")) -> barplot_pathway1

pp[[i]]<-barplot_pathway1 %>% ggplot(aes(cluster_id, expr ,fill=absmy))+

geom_bar(stat='identity') + theme_bw()+ xlab('')+ylab('ES')+

theme(axis.text.x = element_text(angle =90,hjust = .9,size = 10,vjust = 0.9))+

ggtitle(max_difference$name[i]) + theme(legend.position="none")+coord_flip()

}

pp[[3]]

#fig3c

fig3c=mg_merge_plot(pp[[1]],pp[[2]],pp[[3]],pp[[4]],pp[[5]],

pp[[6]],pp[[7]],pp[[8]],pp[[9]],pp[[10]],

pp[[11]],pp[[12]],pp[[13]],pp[[14]],pp[[15]],

pp[[16]],pp[[17]],pp[[18]],pp[[19]],pp[[20]],

ncol=5,nrow=4)

fig3c

savePDF(filename = 'PDFs/fig3c.pdf',plot = fig3c,width = 25,height = 20)

#7、cellphonedb#####

exp=as.matrix(GetAssayData(sce,slot = "counts"))

exp[1:4,1:4]

exp=data.frame(Gene=rownames(exp),exp)

exp[1:4,1:4]

colnames(exp)=gsub('\\.','-',colnames(exp))

write.table(exp,file = 'sce.count.txt',quote = F,row.names = F,sep='\t')

cell_type=merge(data.frame(cell=rownames(sce@meta.data),sce@meta.data),

data.frame(seurat_clusters=cell_anno1$seraut_cluster,

cell_type=cell_anno1$cell_type),

by='seurat_clusters')

#cell_type1=cell_type[,c("cell","cell_type")]

#cell_type1$cell_type=gsub('[+, ,-]','_',cell_type1$cell_type)

cell_type1=cell_type[,c("cell","seurat_clusters")]

cell_type1$seurat_clusters=paste0('C',cell_type1$seurat_clusters)

colnames(cell_type1)[2]='cell_type'

cell_type1$cell=gsub('\\.','-',cell_type1$cell)

write.table(cell_type1,file = 'sce.meta.txt',quote = F,row.names = F,sep='\t')

meta_data=paste0(getwd(),'/sce.meta.txt')

count_data=paste0(getwd(),'/sce.count.txt')

dirname=paste0(getwd(),'/cellphonedb_output')

genename=c('ensembl','gene_name')[2]

mg_cellphonedb<-function(meta_data,count_data,dirname,genename){

command=paste0('/home/wangtl/miniconda/envs/R4/bin/cellphonedb method statistical_analysis --output-path ',dirname,' --counts-data ',genename,' --iterations=1000 --threads=30 ',meta_data,' ',count_data)

command1=paste0('/home/wangtl/miniconda/envs/R4/bin/cellphonedb plot dot_plot --pvalues-path ',dirname,'/pvalues.txt ','--means-path ',dirname,'/means.txt',' --output-path ',dirname,'/ --output-name sce.dotplot.pdf')

command2=paste0('/home/wangtl/miniconda/envs/R4/bin/cellphonedb plot heatmap_plot --pvalues-path ',dirname,'/pvalues.txt',' --output-path ',dirname,'/ --count-name heatmap_count.pdf --log-name heatmap_log_count.pdf --count-network-name count_network.txt --interaction-count-name interactions_count.txt --pvalue 0.05 ',meta_data)

grep_out<-system(command, intern = F)

grep_out1<-system(command1, intern = F)

grep_out2<-system(command2, intern = F)

}

mg_cellphonedb(meta_data,count_data,dirname,genename)

mynet <- read.delim('cellphonedb_output/count_network.txt', check.names = FALSE)

library(igraph)

# #替换亚群的名称

# cell_anno2=cell_anno1

# cell_anno2$seraut_cluster=paste0('C',cell_anno2$seraut_cluster)

# head(mynet)

# for (i in as.character(cell_anno2$seraut_cluster)){

# print(i)

# mynet$SOURCE=gsub(paste0('^',i,'$'),cell_anno2[which(cell_anno2$seraut_cluster==i),"cell_type"],mynet$SOURCE)

# mynet$TARGET=gsub(i,cell_anno2[which(cell_anno2$seraut_cluster==i),"cell_type"],mynet$TARGET)

# }

unique(mynet$SOURCE)

table(mynet$count)

mynet %>% filter(count>0) -> mynet

head(mynet)

write.table(mynet,'cellphonedb_output/count_network111.txt',sep='\t',quote = F,row.names = F)

net<- graph_from_data_frame(mynet, directed = F)

#点的大小

E(net)$width <- E(net)$count/5 # 边点权重（粗细）

#布局

gg=igraph::layout_with_gem(net)

allcolour=c("#DC143C","#0000FF","#20B2AA","#FFA500","#9370DB",

"#98FB98","#F08080","#1E90FF","#7CFC00","#FFFF00",

"#808000","#FF00FF","#FA8072","#7B68EE","#9400D3",

"#800080","#A0522D","#D2B48C","#D2691E","#87CEEB",

"#40E0D0","#5F9EA0","#FF1493",

"#FFE4B5","#8A2BE2","#228B22","#E9967A","#4682B4",

"#32CD32","#F0E68C","#FFFFE0","#EE82EE","#FF6347",

"#6A5ACD","#9932CC","#8B008B","#8B4513","#DEB887")

pdf('cellphonedb_output/cell_cell_network.pdf',he=9,wi=9)

plot(net, edge.arrow.size=.1,

edge.curved=0,

vertex.color=allcolour,

vertex.frame.color="#555555",

vertex.label.color="black",

layout = gg,

vertex.label.cex=.7)

# plot(net,layout=gg,vertex.color=allcolour,vertex.frame.color="#555555",

# vertex.label.color="black",vertex.label.cex=.7)

dev.off()

length(unique(mynet$SOURCE))

pdf('cellphonedb_output/all_cell_cell_network.pdf',he=9,wi=15)

par(mfrow=c(5,4), mar=c(.3,.3,.3,.3))

for (i in 1:length(unique(mynet$SOURCE)) ){

net1<-net

E(net1)[map(unique(mynet$SOURCE),function(x) {

get.edge.ids(net,vp = c(unique(mynet$SOURCE)[i],x))

})%>% unlist()]$color <- allcolour[i]

plot(net1, edge.arrow.size=.1,

edge.curved=0.4,

vertex.color=allcolour,

vertex.frame.color="#555555",

vertex.label.color="black",

layout = gg,

vertex.label.cex=1)

}

dev.off()

#绘制点图

mypvals <- read.delim("cellphonedb_output/pvalues.txt", check.names = FALSE)

mymeans <- read.delim("cellphonedb_output/means.txt", check.names = FALSE)

# 这些基因list很有意思啊，建议保存

chemokines <- grep("^CXC|CCL|CCR|CX3|XCL|XCR", mymeans$interacting_pair,value = T)

th1 <- grep("IL2|IL12|IL18|IL27|IFNG|IL10|TNF$|TNF |LTA|LTB|STAT1|CCR5|CXCR3|IL12RB1|IFNGR1|TBX21|STAT4",

mymeans$interacting_pair,value = T)

th2 <- grep("IL4|IL5|IL25|IL10|IL13|AREG|STAT6|GATA3|IL4R",

mymeans$interacting_pair,value = T)

th17 <- grep("IL21|IL22|IL24|IL26|IL17A|IL17A|IL17F|IL17RA|IL10|RORC|RORA|STAT3|CCR4|CCR6|IL23RA|TGFB",

mymeans$interacting_pair,value = T)

treg <- grep("IL35|IL10|FOXP3|IL2RA|TGFB", mymeans$interacting_pair,value = T)

costimulatory <- grep("CD86|CD80|CD48|LILRB2|LILRB4|TNF|CD2|ICAM|SLAM|LT[AB]|NECTIN2|CD40|CD70|CD27|CD28|CD58|TSLP|PVR|CD44|CD55|CD[1-9]",

mymeans$interacting_pair,value = T)

coinhibitory <- grep("SIRP|CD47|ICOS|TIGIT|CTLA4|PDCD1|CD274|LAG3|HAVCR|VSIR",

mymeans$interacting_pair,value = T)

niche <- grep("CSF", mymeans$interacting_pair,value = T)

cell_anno2

#每一个亚群绘制点图

doplot_cell<-function(mymeans,mypvals,cell_name){

mymeans %>% dplyr::filter(interacting_pair %in% costimulatory)%>%

dplyr::select("interacting_pair",starts_with(cell_name),ends_with(cell_name)) %>%

reshape2::melt() -> meansdf

colnames(meansdf)<- c("interacting_pair","CC","means")

mypvals %>% dplyr::filter(interacting_pair %in% costimulatory)%>%

dplyr::select("interacting_pair",starts_with(cell_name),ends_with(cell_name))%>%

reshape2::melt()-> pvalsdf

colnames(pvalsdf)<- c("interacting_pair","CC","pvals")

pvalsdf$joinlab<- paste0(pvalsdf$interacting_pair,"_",pvalsdf$CC)

meansdf$joinlab<- paste0(meansdf$interacting_pair,"_",meansdf$CC)

pldf <- merge(pvalsdf,meansdf,by = "joinlab")

summary((filter(pldf,means >1))$means)

cellphone_doplot_sel<-pldf%>% filter(means >1) %>%

ggplot(aes(CC.x,interacting_pair.x) )+

geom_point(aes(color=means,size=-log10(pvals)) ) +

scale_size_continuous(range = c(1,3))+xlab('')+ylab('')+

scale_color_gradient2(high="red",mid = "yellow",low ="darkblue",midpoint = 25 )+ theme_bw()+

theme(axis.text.x = element_text(angle = -45,hjust = -0.1,vjust = 0.8))

return(cellphone_doplot_sel)

}

#

doplot_C0=doplot_cell(mymeans,mypvals,cell_name='C0')

doplot_C0

doplot_C1=doplot_cell(mymeans,mypvals,cell_name='C1')

doplot_C1

doplot_C2=doplot_cell(mymeans,mypvals,cell_name='C2')

doplot_C2

doplot_C3=doplot_cell(mymeans,mypvals,cell_name='C3')

doplot_C3

doplot_C4=doplot_cell(mymeans,mypvals,cell_name='C4')

doplot_C4

doplot_C5=doplot_cell(mymeans,mypvals,cell_name='C5')

doplot_C5

doplot_C6=doplot_cell(mymeans,mypvals,cell_name='C6')

doplot_C6

doplot_C7=doplot_cell(mymeans,mypvals,cell_name='C7')

doplot_C7

doplot_C8=doplot_cell(mymeans,mypvals,cell_name='C8')

doplot_C8

doplot_C9=doplot_cell(mymeans,mypvals,cell_name='C9')

doplot_C9

doplot_C10=doplot_cell(mymeans,mypvals,cell_name='C10')

doplot_C10

doplot_C11=doplot_cell(mymeans,mypvals,cell_name='C11')

doplot_C11

doplot_C12=doplot_cell(mymeans,mypvals,cell_name='C12')

doplot_C12

doplot_C13=doplot_cell(mymeans,mypvals,cell_name='C13')

doplot_C13

doplot_C14=doplot_cell(mymeans,mypvals,cell_name='C14')

doplot_C14

doplot_C15=doplot_cell(mymeans,mypvals,cell_name='C15')

doplot_C15

doplot_C16=doplot_cell(mymeans,mypvals,cell_name='C16')

doplot_C16

doplot_C17=doplot_cell(mymeans,mypvals,cell_name='C17')

doplot_C17

doplot_C18=doplot_cell(mymeans,mypvals,cell_name='C18')

doplot_C18

doplot_C19=doplot_cell(mymeans,mypvals,cell_name='C19')

doplot_C19

doplot_c4.10.18.16<-mg_merge_plot(doplot_C4,doplot_C10,doplot_C18,doplot_C16,ncol=2,nrow = 2,labels = c('A','B','C','D'))

doplot_c4.10.18.16

savePDF('cellphonedb_output/doplot_c4.10.18.16.pdf',doplot_c4.10.18.16,height = 15,wi=20)

# doplot_merge<-mg_merge_plot(doplot_C0,doplot_C1,doplot_C2,doplot_C3,doplot_C4,

# doplot_C5,doplot_C6,doplot_C7,doplot_C8,ncol=3,nrow = 3)

#

# savePDF('cellphonedb_output/C9.dotplot.pdf',doplot_C9,height = 9,wi=9)

# savePDF('cellphonedb_output/Cell.dotplot.pdf',doplot_merge,height = 15,wi=20)

#8、细胞通讯cellchart####

cell_type=merge(data.frame(cell=rownames(sce@meta.data),sce@meta.data),

data.frame(seurat_clusters=cell_anno1$seraut_cluster,

cell_type=cell_anno1$cell_type),

by='seurat_clusters')

head(cell_type)

cell_type$cell=gsub('\\.','-',cell_type$cell)

rownames(cell_type)=cell_type$cell

dat_exp_cell<-list(exp=as.matrix(GetAssayData(sce,slot = "counts")),cell_type=cell_type)

save(dat_exp_cell,file = 'dat_exp_cell.RData')

cellchat_data=paste0(getwd(),'/dat_exp_cell.RData')

cellchat_cell<-function(cellchat_data){

library(CellChat)

library(patchwork)

load(cellchat_data)

path=paste0('/',paste(unlist(strsplit(cellchat_data,'/'))[-c(1,length(unlist(strsplit(cellchat_data,'/'))))],collapse = '/'))

data.input=normalizeData(dat_exp_cell$exp, scale.factor = 10000, do.log = TRUE)

meta = dat_exp_cell$cell_type

cellchat <- createCellChat(object = data.input, meta = meta, group.by = "cell_type")

cellchat <- addMeta(cellchat, meta = meta)

cellchat <- setIdent(cellchat, ident.use = "cell_type")

levels(cellchat@idents)

groupSize <- as.numeric(table(cellchat@idents))

CellChatDB=CellChatDB.human

#showDatabaseCategory(CellChatDB)

## use Secreted Signaling for cell-cell communication analysis

#CellChatDB.use <- subsetDB(CellChatDB, search = "Secreted Signaling")

CellChatDB.use <- CellChatDB

cellchat@DB <- CellChatDB.use

cellchat <- subsetData(cellchat)

future::plan("multiprocess", workers = 4)

cellchat <- identifyOverExpressedGenes(cellchat)

cellchat <- identifyOverExpressedInteractions(cellchat)

cellchat <- projectData(cellchat, PPI.human)

#根据表达值推测细胞互做的概率

cellchat <- computeCommunProb(cellchat)

cellchat <- filterCommunication(cellchat,min.cells=10)

df.net <- subsetCommunication(cellchat)

#推断信号通路水平的细胞通讯网络

cellchat <- computeCommunProbPathway(cellchat)

df.netp <- subsetCommunication(cellchat,slot.name='netP')

#统计细胞和细胞之间通信的数量（有多少个配体-受体对）和强度（概率）

cellchat <- aggregateNet(cellchat)

#计算每种细胞各有多少个

groupSize <- as.numeric(table(cellchat@idents))

pdf(paste0(path,'/net_number_strength.pdf'),he=9,wi=15,onefile = F)

par(mfrow = c(1,2))

netVisual_circle(cellchat@net$count, vertex.weight = groupSize,

weight.scale = T, label.edge= F, title.name = "Number of interactions")

netVisual_circle(cellchat@net$weight, vertex.weight = groupSize, weight.scale = T,

label.edge= F, title.name = "Interaction weights/strength")

dev.off()

mat <- cellchat@net$count

cell_num=length(as.character(unique(meta$cell_type)))

cell_col=ceiling(sqrt(cell_num))

cell_row=ceiling(cell_num/cell_col)

pdf(paste0(path,'/net_number_individual.pdf'),he=15,wi=15)

par(mfrow = c(cell_row,cell_col), xpd=TRUE)

for (i in 1:nrow(mat)) {

mat2 <- matrix(0, nrow = nrow(mat), ncol = ncol(mat), dimnames = dimnames(mat))

mat2[i, ] <- mat[i, ]

netVisual_circle(mat2, vertex.weight = groupSize,

weight.scale = T, arrow.width = 0.2,

arrow.size = 0.1, edge.weight.max = max(mat),

title.name = rownames(mat)[i])

}

dev.off()

cellchat_result<-list(df.net=df.net,df.netp=df.netp,cellchat=cellchat)

save(cellchat_result,file = paste0(path,'/cellchat_result.RData'))

}

mg_cellchat_cell <- function(cellchat_data){

command=paste0('R_LIBS_SITE=" " R_LIBS_USER=" " /home/wangtl/miniconda/envs/R4/bin/Rscript ','/pub1/data/mg_projects/users/wangtl/public/cellchat_cell.R ',cellchat_data)

print(paste0('cellchat_cell CMD:',command))

grep_out<-system(command, intern = F)

cat(grep_out)

}

mg_cellchat_cell(cellchat_data)

load('cellchat_result.RData')

cellchat_result$df.net

cellchat_result$df.netp

cellchat_result$cellchat

#9、CytoTRACE 细胞轨迹#####

cytotrace_cell<-function(sce_dat){

library("CytoTRACE")

load(sce_dat)

path=paste0('/',paste(unlist(strsplit(sce_dat,'/'))[-c(1,length(unlist(strsplit(sce_dat,'/'))))],collapse = '/'))

exp.rawdata <- as.matrix(sce@assays$RNA@counts)

cytotrace_results=list()

results <- CytoTRACE(exp.rawdata, ncores = 10, subsamplesize = 1000)

umap_embeddings <- Seurat::Embeddings(sce,reduction = "umap")

emb <- as.data.frame(umap_embeddings)

cytogenes_plot<-plotCytoGenes(results,numOfGenes = 10)

cytotrace_plot<-plotCytoTRACE(results,emb = emb)

cytotrace_results=list(results=results,emb=emb)

save(cytotrace_results,file = paste0(path,'/cytotrace_results.RData'))

}

plotCytoGenes<-function (cyto_obj = NULL, numOfGenes = 10, colors = c("darkred","navyblue")){

cytoGenes <- cyto_obj$cytoGenes

top_k <- tail(sort(cytoGenes), numOfGenes)

bottom_k <- head(sort(cytoGenes), numOfGenes)

final_list <- data.frame(CytoGenes = c(top_k, bottom_k))

final_list <- cbind(final_list, Gene = rownames(final_list))

final_list <- final_list[order(final_list$CytoGenes), ]

p <- ggplot2::ggplot(data = final_list, ggplot2::aes(x = Gene,

y = CytoGenes, color = CytoGenes, fill = CytoGenes)) +

ggplot2::geom_bar(position = "dodge", stat = "identity") +

ggplot2::coord_flip() + ggplot2::scale_color_gradient(low = colors[2],

high = colors[1], guide = F) + ggplot2::scale_fill_gradient(low = adjustcolor(colors[2],

0.3), high = adjustcolor(colors[1], 0.3), guide = F) +

ggplot2::scale_x_discrete(limits = final_list$Gene) +

ggplot2::scale_y_continuous(breaks = as.numeric(formatC(round(signif(seq(round(min(cytoGenes,

na.rm = T) - 0.05, 1), round(max(cytoGenes, na.rm = T) +

0.05, 1), 0.2), 1), 1), digits = 1))) + ggplot2::ylab("Correlation with CytoTRACE") +

ggplot2::theme(legend.title = ggplot2::element_blank(),

legend.position = "none", legend.background = ggplot2::element_rect(),

axis.text.x = ggplot2::element_text(color = "black",

size = 15), axis.text.y = ggplot2::element_text(color = "black",

size = 15), axis.title.x = ggplot2::element_text(color = "black",

size = 20, margin = ggplot2::margin(t = 10, r = 0,

b = 0, l = 0)), axis.title.y = ggplot2::element_text(color = "black",

size = 20, margin = ggplot2::margin(t = 0, r = 20,

b = 0, l = 0)), axis.ticks.x = ggplot2::element_line(color = "black"),

axis.ticks.y = ggplot2::element_line(color = "black"),

axis.ticks.length = ggplot2::unit(0.2, "cm"), strip.background = ggplot2::element_blank(),

strip.text = ggplot2::element_text(colour = "black",

size = 17), axis.line = ggplot2::element_line(colour = "black"),

panel.grid.major = ggplot2::element_blank(), panel.grid.minor = ggplot2::element_blank(),

panel.border = ggplot2::element_blank(), panel.background = ggplot2::element_blank(),

plot.margin = ggplot2::margin(t = 0.5, r = 0.5, b = 0.5,

l = 0.5, unit = "cm"), panel.spacing.x = ggplot2::unit(1.5,

"lines"))

return(p)

}

plotCytoTRACE<-function (cyto_obj = NULL, phenotype = NULL, gene = NULL,

colors = NULL,emb = NULL, otherName = NULL, otherValue = NULL){

mat <- cyto_obj$exprMatrix

cyto <- cyto_obj$CytoTRACE

if (!is.null(phenotype)) {

if (ncol(mat) != length(phenotype)) {

stop("The number of phenotype labels provided does not match the number of cells in the CytoTRACE object.",

type = "error")

}

if (length(intersect(colnames(mat), names(phenotype))) !=

length(phenotype)) {

stop("The names of the phenotype labels do no match the names in the CytoTRACE object.",

type = "error")

}

if (max(nchar(phenotype)) > 25) {

warning("Phenotype labels exceed 25 characters. This may result in overcrowded text in some of the visualizations.")

}

pheno <- phenotype[colnames(mat)]

}else {

pheno <- NULL

}

if (!is.null(gene)) {

gene <- tolower(gene)

lower_case_names <- tolower(rownames(mat))

if (gene %in% lower_case_names) {

gene_exp <- mat[lower_case_names %in% gene, ]

gene2 <- rownames(mat)[which(lower_case_names == gene)]

}

else {

gene_exp <- integer(length(mat[1, ]))

gene2 <- gene

}

}else {

gene_exp <- NULL

}

if (!is.null(otherValue)) {

gene_exp <- otherValue

if (length(gene_exp) != length(cyto)) {

stop("The length of the 'otherValue' object does not match the length of the CytoTRACE object.")

}

}

if (!is.null(otherName)) {

gene2 <- ""

}

mat <- mat[, !is.na(cyto)]

pheno <- pheno[!is.na(cyto)]

gene_exp <- gene_exp[!is.na(cyto)]

cyto <- cyto[!is.na(cyto)]

dotsne <- function(mat, perplexity) {

.get_variable_gene <- function(m) {

df <- data.frame(mean = colMeans(m), cv = apply(m, 2, sd)/colMeans(m), var = apply(m, 2, var))

df$dispersion <- with(df, var/mean)

df$mean_bin <- with(df, cut(mean, breaks = c(-Inf,quantile(mean, seq(0.1, 1, 0.05)), Inf)))

var_by_bin <- plyr::ddply(df, "mean_bin", function(x) {

data.frame(bin_median = median(x$dispersion),

bin_mad = mad(x$dispersion))

})

df$bin_disp_median <- var_by_bin$bin_median[match(df$mean_bin,var_by_bin$mean_bin)]

df$bin_disp_mad <- var_by_bin$bin_mad[match(df$mean_bin,var_by_bin$mean_bin)]

df$dispersion_norm <- with(df, abs(dispersion - bin_disp_median)/bin_disp_mad)

df

}

.do_propack <- function(x, n) {

use_genes <- which(colSums(x) > 1)

m <- x[, use_genes]

bc_tot <- rowSums(m)

median_tot <- median(bc_tot)

m <- sweep(m, 1, median_tot/bc_tot, "*")

m <- sweep(m, 2, colMeans(m), "-")

m <- sweep(m, 2, apply(m, 2, sd), "/")

ppk <- propack.svd(as.matrix(m), neig = n)

pca <- t(ppk$d * t(ppk$u))

list(ppk = ppk, pca = pca, m = m, use_genes = use_genes)

}

.normalize_by_umi <- function(x) {

cs <- colSums(x)

x_use_genes <- which(cs >= 1)

x_filt <- x[, x_use_genes]

rs <- rowSums(x_filt)

rs_med <- median(rs)

x_norm <- x_filt/(rs/rs_med + 1e-15)

list(m = x_norm, use_genes = x_use_genes)

}

matt <- t(mat)

m_n <- matt

l <- .normalize_by_umi(matt)

m_n <- l$m

df <- .get_variable_gene(m_n)

disp_cut_off <- sort(df$dispersion_norm, decreasing = T)[1000]

df$used <- df$dispersion_norm >= disp_cut_off

set.seed(0)

m_n_1000 <- m_n[, head(order(-df$dispersion_norm), 1000)]

tsne <- Rtsne::Rtsne(m_n_1000, pca = T, check_duplicates = FALSE,

perplexity = perplexity)

tsn <- tsne$Y

rownames(tsn) <- colnames(mat)

return(tsn)

}

if (!is.null(emb)) {

if (length(intersect(colnames(mat), rownames(emb))) !=

ncol(mat)) {

stop("The samples in the embedding table do not match the samples in the CytoTRACE object.",

type = "error")

}

emb <- emb[colnames(mat), ]

tsne.proj <- emb

}else if (length(grep("coord", names(cyto_obj))) > 0) {

emb <- cyto_obj$coord

tsne.proj <- emb

}else {

message("Running t-SNE. To use your own coordinates, use the 'emb' flag.")

n_samples <- ncol(mat)

perplexity <- if (n_samples > 100)

30

else 10

tsne.proj <- data.frame(dotsne(mat, perplexity))

}

output_data <- data.frame(CytoTRACE = cyto, Component1 = tsne.proj[,1], Component2 = tsne.proj[, 2])

if (!is.null(pheno)){output_data$Phenotype <- pheno}

if (!is.null(gene_exp)){output_data$Gene <- gene_exp}

drawPlots <- function(data, colors, gene2) {

data <- data[order(data$CytoTRACE), ]

emb <- cbind.data.frame(Component1 = data$Component1,

Component2 = data$Component2)

temp <- RColorBrewer::brewer.pal(11, "Spectral")

temp[6] <- "gold"

rbPal <- colorRampPalette(temp)

if (!is.null(colors)) {

rbPal <- colorRampPalette(colors)

}

if (length(data$CytoTRACE) <= 200) {

ptsize <- 4

}

else if (length(data$CytoTRACE) > 200 & length(data$CytoTRACE) < 1000) {

ptsize <- 3.5

}

else if (length(data$CytoTRACE) > 1000 & length(data$CytoTRACE) < 2000) {

ptsize <- 3

}

else if (length(data$CytoTRACE) > 2000 & length(data$CytoTRACE) < 5000) {

ptsize <- 2

}

else if (length(data$CytoTRACE) > 5000 & length(data$CytoTRACE) < 10000) {

ptsize <- 1

}

else if (length(data$CytoTRACE) >= 10000) {

ptsize <- 0.5

}

if (!is.null(data$Phenotype)) {

data$Phenotype <- substring(data$Phenotype, 1, 25)

suppressWarnings(aggregated <- aggregate(data, by = list(data$Phenotype),

FUN = median))

levels = as.character(aggregated[order(-aggregated$CytoTRACE),

]$Group.1)

data$Phenotype <- factor(data$Phenotype, levels = levels)

pheno_levels <- levels(data$Phenotype)

cols <- rbPal(length(pheno_levels))

names(cols) <- pheno_levels

pdf('CytoTRACE_cell_type_boxplot.pdf')

par(mar = c(log(max(nchar(pheno_levels)) + 2, 2) *

3.25, 6, 2, 1), xpd = NA)

boxplot(data$CytoTRACE ~ data$Phenotype, outline = F,

las = 2, xaxt = "n", yaxt = "n", staplelwd = 2,

medlwd = 2, whisklty = 1, border = cols,

col = adjustcolor(cols,alpha.f = 0.25),

ylab = "Predicted ordering by CytoTRACE",

xlab = "", cex.lab = 1.75, frame.plot = F,

xlim = c(0, length(pheno_levels) + 0.5),

ylim = c(min(data$CytoTRACE), 1))

mtext("Cell phenotypes", cex = 1.75, side = 1, line = round(max(nchar(pheno_levels))/2,

1) + 1)

axis(1, pos = 0, at = 1:length(pheno_levels), labels = FALSE)

text(x = seq_along(pheno_levels), y = -0.05, srt = 45,

adj = 1, labels = pheno_levels, xpd = TRUE, cex = 1.5)

segments(0, 0, length(pheno_levels) + 0.5, 0)

axis(2, pos = 0, at = seq(0, 1, 0.2), labels = format(seq(0,

1, 0.2), nsmall = 1), las = 2, cex.axis = 1.5)

segments(0, 0, 0, 1)

stripchart(data$CytoTRACE ~ data$Phenotype, vertical = TRUE,

method = "jitter", add = TRUE, pch = 16, col = cols,

bg = "white", lwd = 1, cex = 2/round(log(length(data$CytoTRACE),10)))

dev.off()

suppressWarnings(aggregated <- aggregate(data, by = list(data$Phenotype),

FUN = mean))

levels = as.character(aggregated[order(-aggregated$CytoTRACE),

]$Group.1)

data$Phenotype <- factor(data$Phenotype, levels = levels)

pheno_levels <- levels(data$Phenotype)

cols <- rbPal(length(pheno_levels))

names(cols) <- pheno_levels

p1 <- ggplot2::ggplot(data, ggplot2::aes(x = emb[, 1], y = emb[, 2])) +

ggplot2::geom_point(ggplot2::aes(colour = Phenotype),size = ptsize) +

ggplot2::scale_colour_manual(values = cols,

guide = ggplot2::guide_legend(frame.colour = "black",

override.aes = list(size = 4))) +

ggplot2::labs(x = colnames(emb)[1],y= colnames(emb)[2], title = "Phenotype") +

ggpubr::theme_pubr() +

ggplot2::theme(legend.text = ggplot2::element_text(size = 14),

legend.title = ggplot2::element_blank(),

plot.title = ggplot2::element_text(size = 21,hjust = 0.5),

axis.title.x = ggplot2::element_text(size = 18),

axis.title.y = ggplot2::element_text(size = 18),

axis.text = ggplot2::element_text(size = 16),

legend.position = "right",

plot.margin = ggplot2::unit(c(0.5,1, 0.5, 1), "cm"))

}

if (!is.null(data$Gene) & sum(data$Gene) != 0) {

p2 <- ggplot2::ggplot(data, ggplot2::aes(x = emb[,1], y = emb[, 2])) +

ggplot2::geom_point(ggplot2::aes(colour = Gene),size = ptsize) +

ggplot2::scale_colour_gradientn(name = ifelse(is.null(otherName),

"Gene\nexpression",

otherName),

colours = rev(rbPal(50)),

guide = ggplot2::guide_colourbar(ticks.colour = "black",

ticks.linewidth = 1,

frame.colour = "black")) +

ggplot2::labs(x = colnames(emb)[1], y = colnames(emb)[2],

title = bquote(italic(.(gene2)))) +

ggpubr::theme_pubr() +

ggplot2::theme(legend.text = ggplot2::element_text(size = 12),

legend.title = ggplot2::element_text(size = 14),

plot.title = ggplot2::element_text(size = 21,hjust = 0.5),

axis.title.x = ggplot2::element_text(size = 18),

axis.title.y = ggplot2::element_text(size = 18),

axis.text = ggplot2::element_text(size = 16),

legend.position = "right", plot.margin = ggplot2::unit(c(0.5,

1, 0.5, 1), "cm"))

}else if (!is.null(data$Gene) & sum(data$Gene) == 0) {

p2 <- ggplot2::ggplot(data, ggplot2::aes(x = emb[, 1], y = emb[, 2])) +

ggplot2::geom_point(ggplot2::aes(x = emb[,1], y = emb[, 2], colour = Gene), size = 0.1) +

ggplot2::scale_colour_gradientn(name = "Gene\nexpression",

colours = "white",

guide = ggplot2::guide_colourbar(ticks.colour = "black",

ticks.linewidth = 1, frame.colour = "black")) +

ggplot2::annotate("text", x = mean(emb[, 1]),

y = mean(emb[, 2]), xmin = min(emb[, 1]),

xmax = max(emb[,1]), ymin = min(emb[, 2]),

ymax = max(emb[, 2]),

label = "Selected gene\nis not expressed in dataset",

size = 6) +

ggplot2::labs(x = colnames(emb)[1], y = colnames(emb)[2], title = bquote(italic(.(gene2)))) +

ggpubr::theme_pubr() +

ggplot2::theme(legend.text = ggplot2::element_text(size = 12),

legend.title = ggplot2::element_text(size = 14),

plot.title = ggplot2::element_text(size = 21,hjust = 0.5),

axis.title.x = ggplot2::element_text(size = 18),

axis.title.y = ggplot2::element_text(size = 18),

axis.text = ggplot2::element_text(size = 16),

legend.position = "right", plot.margin = ggplot2::unit(c(0.5,1, 0.5, 1), "cm"))

}

p3 <- ggplot2::ggplot(data, ggplot2::aes(x = emb[, 1], y = emb[, 2])) +

ggplot2::geom_point(ggplot2::aes(colour = CytoTRACE),size = ptsize) +

ggplot2::scale_colour_gradientn(name = "Predicted\norder",

colours = rev(rbPal(50)),

guide = ggplot2::guide_colourbar(ticks.colour = "black",

ticks.linewidth = 1,

frame.colour = "black"),

breaks = seq(0, 1, 0.2),

labels = c("0.0 (More diff.)",0.2, 0.4, 0.6, 0.8, "1.0 (Less diff.)")) +

ggplot2::labs(x = colnames(emb)[1],

y = colnames(emb)[2], title = "CytoTRACE") +

ggpubr::theme_pubr() +

ggplot2::theme(legend.text = ggplot2::element_text(size = 12),

legend.title = ggplot2::element_text(size = 14),

plot.title = ggplot2::element_text(size = 21, hjust = 0.5),

axis.title.x = ggplot2::element_text(size = 18),

axis.title.y = ggplot2::element_text(size = 18),

axis.text = ggplot2::element_text(size = 16),

legend.position = "left", plot.margin = ggplot2::unit(c(0.5,1, 0.5, 1), "cm"))

if (!is.null(data$Phenotype) & !is.null(data$Gene)) {

mp <- egg::ggarrange(p3, p1, p2, nrow = 1, newpage = T)

}

else if (!is.null(data$Phenotype) & is.null(data$Gene)) {

mp <- egg::ggarrange(p3, p1, nrow = 1)

}

else if (is.null(data$Phenotype) & !is.null(data$Gene)) {

mp <- egg::ggarrange(p3, p2, nrow = 1)

}

else if (is.null(data$Phenotype) & is.null(data$Gene)) {

mp <- egg::ggarrange(p3, nrow = 1)

}

return(mp)

}

p=drawPlots(output_data, colors, gene2)

return(list(plot=p,file=output_data))

}

mg_cytotrace_cell <- function(sce_dat){

command=paste0('R_LIBS_SITE=" " R_LIBS_USER=" " /home/wangtl/miniconda/envs/R4/bin/Rscript ','/pub1/data/mg_projects/users/wangtl/public/cytotrace_cell.R ',sce_dat)

print(paste0('cytotrace_cell CMD:',command))

grep_out<-system(command, intern = F)

cat(grep_out)

}

sce_dat=paste0(getwd(),'/sce1.RData')

mg_cytotrace_cell(sce_dat)

load('cytotrace_results.RData')

cytogenes_plot<-plotCytoGenes(cytotrace_results$results,numOfGenes = 10)

cytogenes_plot

celltype <- as.character(sce$seurat_clusters)

col <- colnames(sce)

phenotype <- cbind(col, celltype)

rownames(phenotype) <- phenotype[,1]

phenotype <- phenotype[,-1]

cytotrace_plot<-plotCytoTRACE(cytotrace_results$results,

phenotype = phenotype,

gene = NULL, emb = cytotrace_results$emb)

cytotrace_plot$plot

sce <- AddMetaData(

object = sce,

metadata = cytotrace_results$results$CytoTRACE,

col.name = "cytoTRACE"

)

save(sce,file = 'sce_cytoTRACE.RData')

w1 <- dittoSeq::dittoDimPlot(sce, var = "cytoTRACE", reduction.use = "umap", legend.size = 10,

main = "cytoTRACE score", do.label = F, labels.repel = F, do.ellipse = F,

labels.highlight = T) +

theme_classic() +

theme(legend.text = element_text(size = 25, color = "black", family = "sans"),

axis.text = element_text(size = 25, color = "black", family = "sans"),

axis.title = element_text(size = 25, color = "black", family = "sans"),

title = element_text(size = 25, color = "black", family = "sans"))

w2 <- dittoSeq::dittoDimPlot(sce, var = "seurat_clusters", reduction.use = "umap", legend.size = 10,

main = "Cell states", do.label = F, labels.repel = F, do.ellipse = F,

labels.highlight = T) +

theme_classic() +

theme(legend.text = element_text(size = 25, color = "black", family = "sans"),

axis.text = element_text(size = 25, color = "black", family = "sans"),

axis.title = element_text(size = 25, color = "black", family = "sans"),

title = element_text(size = 25, color = "black", family = "sans"))

cytotrace_dimplot<-mg_merge_plot(w1, w2, ncol = 2,nrow=1,labels = c('A','B'))

cytotrace_dimplot

savePDF(filename = 'PDFs/cytotrace_dimplot.pdf',plot = cytotrace_dimplot,he=9,wi=12)

###################单细胞绘图#######################

#### umap plot

load('sce1.RData')

sc_umap = DimPlot(sce,cols=mycolor,

reduction="umap",

label = "T",

pt.size = 0.2,

label.size = 5) +

theme(axis.line = element_line(size=0.1, colour = "black"),

#axis.text = element_blank(),

#axis.title = element_blank(),

axis.ticks = element_blank()

)

#Tsne_embeddings <- Embeddings(sce,reduction = "tsne")

sc_umap_group1 = DimPlot(sce,cols=mycolor,group.by='Group1',

reduction="umap",

label = "T",

pt.size = 0.2,

label.size = 0) +

theme(axis.line = element_line(size=0.1, colour = "black"),

#axis.text = element_blank(),

#axis.title = element_blank(),

axis.ticks = element_blank()

)

sc_umap_group1

sc_umap_group2 = DimPlot(sce,cols=mycolor,group.by='Group2',

reduction="umap",

label = "T",

pt.size = 0.2,

label.size = 0) +

theme(axis.line = element_line(size=0.1, colour = "black"),

#axis.text = element_blank(),

#axis.title = element_blank(),

axis.ticks = element_blank()

)

sc_umap_group2

umap_merge<-mg_merge_plot(sc_umap_group1,sc_umap_group2,sc_umap,ncol=2,nrow=2)

savePDF('PDFs/umap_merge.pdf',plot = umap_merge,he=9,wi=9)

#补：TCGA数据做完再补特征基因表达###

#先查看这些基因的高低表达是否有意义####

c4_10_feat<-c('TNFRSF13C','CCR7', 'ACAP1',

'TCL1A', 'RGS13')

test_dat1<-t(tcga_dat_T[c4_10_feat,])

test_dat1=apply(test_dat1,2,function(x){

y=ifelse(x>median(x),'High','Low')

return(y)

})

test_dat1=crbind2DataFrame(test_dat1)

tcga_cli=crbind2DataFrame(tcga_cli)

plotKMCox_1=function(dat,n){

library(survival)

library(ggsci)

mypal = pal_jama(alpha = 0.7)(7)

colnames(dat)=c('time','status','groups')

sdf<-survdiff(Surv(time,status) ~ groups,data=dat)

#print((sdf))

p<-pchisq(sdf$chisq,length(sdf$n)-1,lower.tail=FALSE)

sf<-survfit(Surv(time,status) ~ groups,data=dat)

colKm=c(mypal[2],mypal[1],mypal[3],mypal[4])

plot(sf, mark.time = TRUE,col=colKm,xlab=paste("Survival time in day","\np=",round(p,5)),ylab = "Survival probabilities",main=n)

legend('topright',paste0(gsub('groups=','',names(sf$strata)),'(N=',sdf$n,')'), col = colKm,

lty = c(1,1, 1, 1),lwd=c(1,1,1,1),merge = TRUE,cex = 0.8)

return(p)

}

par(mfrow=c(3, 2))

for (a in colnames(test_dat1)) {

plotKMCox_1(data.frame(tcga_cli$OS.time,

tcga_cli$OS,

test_dat1[tcga_cli$Samples,a])

,a)

}

c4_10_feat1<-c('TNFRSF13C','ACAP1',

'TCL1A', 'RGS13')

c9_15_18_feat<-c('DEFB1', 'TFF1','CRISP3','CLDN3', 'CEACAM6','WFDC2', 'MUC5B' ,'SLC3A1', 'TSPAN8',' EDN1','GPX2',

'CELA3A','CPB1', 'PNLIPRP2', 'GP2', 'SYCN', 'CELA2A', 'CELA3B', 'ALB', 'CUZD1', 'SERPINI2', 'AC009078.2', 'KLK1', 'PDIA2', 'ERP27', 'MT1G', 'IGFBP2',

'LINC01681', 'SPATA22', 'CMC2', 'IER3-AS1', 'SCGB3A1', 'AC093484.3', 'RGS5', 'AL138963.3', 'TIMP3', 'VIM-AS1', 'AC243829.4', 'AC011603.2', 'ICK', 'AC010761.1', 'LPL')

test_dat1<-t(tcga_dat_T[intersect(c9_15_18_feat,rownames(tcga_dat_T)),])

test_dat1=apply(test_dat1,2,function(x){

y=ifelse(x>median(x),'High','Low')

return(y)

})

test_dat1=crbind2DataFrame(test_dat1)

par(mfrow=c(4, 4))

for (a in colnames(test_dat1)[26:ncol(test_dat1)]) {

plotKMCox_1(data.frame(tcga_cli$OS.time,

tcga_cli$OS,

test_dat1[tcga_cli$Samples,a])

,a)

}

c9_15_18_feat1<-c('DEFB1','CLDN3', 'MT1G', 'IGFBP2','CMC2', 'IER3-AS1')

c0_1_feat<-c('ADAMDEC1', 'CLEC4E', 'KCNMA1', 'SELENOP', 'PLTP', 'CH25H', 'SLC1A3', 'NUPR1', 'RNASE1',

'MP12', 'MRC1', 'IL1RN', 'IFI6', 'ALOX5AP', 'VSIG4', 'FBP1', 'MARCO', 'CXCL1', 'ISG15' )

test_dat1<-t(tcga_dat_T[intersect(c0_1_feat,rownames(tcga_dat_T)),])

test_dat1=apply(test_dat1,2,function(x){

y=ifelse(x>median(x),'High','Low')

return(y)

})

test_dat1=crbind2DataFrame(test_dat1)

ncol(test_dat1)

par(mfrow=c(3, 3))

for (a in colnames(test_dat1)[10:18]) {

plotKMCox_1(data.frame(tcga_cli$OS.time,

tcga_cli$OS,

test_dat1[tcga_cli$Samples,a])

,a)

}

c0_1_feat1<-c('ADAMDEC1', 'CLEC4E','IL1RN','MARCO')

c6_7_16_feat<-c('KRT5', 'CALB1', 'KRT16', 'S100P', 'SERPINB3', 'DSP', 'LAMA3', 'SAA1', 'MIR205HG', 'LAMC2', 'S100A16', 'COL17A1', 'AQP3', 'CP', 'FAM83A', 'CAV1', 'S100A14', 'KRT7', 'KRT6B', 'MUC1', 'SDC1', 'GJB2', 'AGR2',

'IL1R2', 'LGALS2',

'S100B', 'CXCL10' , 'CLEC5A', 'HMMR', 'CDK1', 'TNF', 'FAM111A', 'TK1', 'SMC2')

test_dat1<-t(tcga_dat_T[intersect(c6_7_16_feat,rownames(tcga_dat_T)),])

test_dat1=apply(test_dat1,2,function(x){

y=ifelse(x>median(x),'High','Low')

return(y)

})

test_dat1=crbind2DataFrame(test_dat1)

ncol(test_dat1)

par(mfrow=c(3, 3))

for (a in colnames(test_dat1)[19:27]) {

plotKMCox_1(data.frame(tcga_cli$OS.time,

tcga_cli$OS,

test_dat1[tcga_cli$Samples,a])

,a)

}

c6_7_16_feat1<-c('LAMA3', 'S100A16','IL1R2', 'LGALS2', 'S100B', 'CXCL10')

c2_11_feat<-c('CXCL13', 'GAS1', 'CHI3L2', 'HILPDA',

'CXCL14', 'IGFBP5', 'CCL2')

test_dat1<-t(tcga_dat_T[intersect(c2_11_feat,rownames(tcga_dat_T)),])

test_dat1=apply(test_dat1,2,function(x){

y=ifelse(x>median(x),'High','Low')

return(y)

})

test_dat1=crbind2DataFrame(test_dat1)

ncol(test_dat1)

par(mfrow=c(3, 3))

for (a in colnames(test_dat1)) {

plotKMCox_1(data.frame(tcga_cli$OS.time,

tcga_cli$OS,

test_dat1[tcga_cli$Samples,a])

,a)

}

c2_11_feat1<-c('CXCL13', 'HILPDA','CXCL14','CCL2')

c5_14_feat<-c('IL7R', 'XCL1', 'KLRB1', 'GZMB', 'MAF', 'SPOCK2', 'TSPYL2',

'CRTAM', 'TIGIT', 'CTSW', 'LINC01871', 'PYHIN1', 'KLRD1', 'GZMA', 'TOP2A', 'HIST1H1E', 'CENPF', 'HIST1H1D', 'NUSAP1', 'ASPM', 'TMPO', 'HIST1H1C', 'TUBB', 'CKS1B', 'ATAD2', 'HIST1H1B', 'NUF2', 'TRDC', 'CENPE', 'ITM2C', 'DNAJC9', 'TNFRSF18', 'PRF1')

test_dat1<-t(tcga_dat_T[intersect(c5_14_feat,rownames(tcga_dat_T)),])

test_dat1=apply(test_dat1,2,function(x){

y=ifelse(x>median(x),'High','Low')

return(y)

})

test_dat1=crbind2DataFrame(test_dat1)

ncol(test_dat1)

par(mfrow=c(3, 3))

for (a in colnames(test_dat1[10:18])) {

plotKMCox_1(data.frame(tcga_cli$OS.time,

tcga_cli$OS,

test_dat1[tcga_cli$Samples,a])

,a)

}

c5_14_feat1<-c('SPOCK2', 'TSPYL2','TOP2A', 'CENPF')

#B cell 4,10

c4_10_feat1

c4_10_vlnplot<-VlnPlot(object = subset(sce, idents = c("4", "10")),

features = c4_10_feat1,ncol=4,

pt.size = 0)

#Cancer cell 9,15,18

c9_15_18_feat1

c9_15_18_vlnplot<-VlnPlot(object = subset(sce, idents = c("9", "15","18")),

features = c9_15_18_feat1,ncol=4,

pt.size = 0)

c9_15_18_vlnplot

#CD1C-CD141- dendritic cell 0,1

c0_1_feat1

c0_1_vlnplot<-VlnPlot(object = subset(sce, idents = c("0", "1")),

features = c0_1_feat1,ncol=4,

pt.size = 0)

c0_1_vlnplot

#CD1C+_B dendritic cell 6,7,16

c6_7_16_feat1

c6_7_16_vlnplot<-VlnPlot(object = subset(sce, idents = c("6", "7","16")),

features = c6_7_16_feat1,ncol=4,

pt.size = 0)

c6_7_16_vlnplot

#Fibroblast 2,11

c2_11_feat1

c2_11_vlnplot<-VlnPlot(object = subset(sce, idents = c("2", "11")),

features = c2_11_feat1,ncol=4,

pt.size = 0)

c2_11_vlnplot

#Natural killer cell 5,14

c5_14_feat1

c5_14_vlnplot<-VlnPlot(object = subset(sce, idents = c("5", "14")),

features = c5_14_feat1,ncol=4,

pt.size = 0)

c5_14_vlnplot

feat_vlnplot1<-mg_merge_plot(c4_10_vlnplot,

c0_1_vlnplot,

c2_11_vlnplot,

c5_14_vlnplot,

#c6_7_16_vlnplot,

#c9_15_18_vlnplot,

ncol=1,nrow=4)

feat_vlnplot2<-mg_merge_plot(c6_7_16_vlnplot,

c9_15_18_vlnplot,

ncol=1,nrow=2)

feat_vlnplot<-mg_merge_plot(feat_vlnplot2,feat_vlnplot1,ncol=1,nrow=2)

savePDF(plot = feat_vlnplot,filename = 'PDFs/feat_vlnplot.pdf',he=15,wi=10)

#10、TCGA数据,提取数据和预处理####

gene_type <- read.delim('origin_datas/GeneTag.genecode.v32.txt',

header = T, stringsAsFactors = F)

gene_type <- gene_type[!duplicated(gene_type$ENSGID), ]

rownames(gene_type) <- gene_type$ENSGID

tcga_dat<-read.delim('origin_datas/TCGA/Merge_TCGA-PAAD_TPM.txt',sep='\t',header = T,row.names = 1,check.names = F)

tcga_dat[1:4,1:4]

tcga_cli<-read.delim('origin_datas/TCGA/Merge_clinical.txt',sep='\t',header = T,check.names = F)

tcga_cli<-tcga_cli[which(tcga_cli$A0_Barcode=='PAAD'),]

tcga_cli <- tcga_cli[, c("A0_Samples", "A1_OS", "A2_Event", "A3_T", "A4_N", "A5_M",

"A6_Stage", "A18_Sex",

"age_at_initial_pathologic_diagnosis")]

colnames(tcga_cli) <- c("Samples", "OS.time", "OS", "T.Stage", "N.Stage", "M.Stage",

"Stage", "Gender", "Age")

head(tcga_cli)

tcga_cli$OS=gsub('Dead',1,tcga_cli$OS)

tcga_cli$OS=gsub('Alive',0,tcga_cli$OS)

#提取肿瘤样本

tcga_sample_T<-colnames(tcga_dat)[substr(colnames(tcga_dat),14,15)=='01']

tcga_dat_T<-tcga_dat[,tcga_sample_T]

colnames(tcga_dat_T)=substr(colnames(tcga_dat_T),1,12)

tcga_sample_com=intersect(colnames(tcga_dat_T),tcga_cli$Samples)

length(tcga_sample_com)

##GETx数集

#样本信息

getx_sample=read.delim('origin_datas/GTEx/GTEX_phenotype.xls',sep = '\t',header = T)

head(getx_sample)

getx_sample=getx_sample[grep('Pancreas',getx_sample$X_primary_site),]

getx_sample=as.character(getx_sample$Sample)

#tpm数据

getx_tpm=read.delim('origin_datas/GTEx/gtex_RSEM_gene_fpkm',sep='\t',header = T,row.names = 1,check.names=F)

rownames(getx_tpm)=str_split_fixed(rownames(getx_tpm), '\\.', 2)[, 1]

com_ensg <- intersect(rownames(getx_tpm), gene_type$ENSGID)

getx_tpm <- getx_tpm[com_ensg, ]

getx_sam_com=intersect(colnames(getx_tpm),getx_sample)

length(getx_sam_com)

getx_tpm=getx_tpm[,getx_sam_com]

getx_tpm$genes <- gene_type[com_ensg, ]$SYMBOL

getx_tpm[1:4,1:4]

getx_tpm=aggregate(.~genes,getx_tpm,mean)

rownames(getx_tpm)=getx_tpm$genes

getx_tpm=getx_tpm[,-1]

save(getx_tpm,file = 'origin_datas/GTEx/getx_tpm.RData')

boxplot(getx_tpm[,1:4])

#合并数据

sam_T=colnames(tcga_dat_T)

sam_N=colnames(getx_tpm)

sameGene=intersect(row.names(getx_tpm),row.names(tcga_dat_T))

merge_tcga=cbind(getx_tpm[sameGene,],log2(tcga_dat_T[sameGene,]+1))

tcga_groups <- data.frame(Samples = c(sam_T, sam_N),

Type = c(rep('Tumor', length(sam_T)),

rep('Normal', length(sam_N))),

row.names = c(sam_T, sam_N),

stringsAsFactors = F)

#数据矫正

library(limma)

boxplot(merge_tcga[,1:4])

merge_tcga_nor=normalizeBetweenArrays(merge_tcga)

boxplot(merge_tcga_nor[,1:4])

dim(merge_tcga_nor)

write.table(data.frame(GeneSymbol=rownames(merge_tcga_nor),merge_tcga_nor),file = 'tcga_exp.txt',row.names = F,quote = F,sep='\t')

#11、亚群丰度预测####

dim(sce.markers)

rownames(sce.markers)

sce@assays$RNA[1:4,1:4]

cell_type_exp<- as.matrix(GetAssayData(sce,slot = "counts"))

cell_type_exp=t(cell_type_exp[unique(sce.markers$gene),])

serat_clus<-as.data.frame(sce$seurat_clusters)

head(serat_clus)

serat_clus=merge(cell_anno1,

data.frame(seraut_cluster=serat_clus[,1],

cell=rownames(serat_clus)),

by='seraut_cluster')

head(serat_clus)

cell_type_exp=merge(data.frame(cell=serat_clus$cell,cell_type=serat_clus$cell_type),

data.frame(cell=rownames(cell_type_exp),cell_type_exp),

by='cell')

cell_type_exp[1:4,1:4]

cell_type_exp=cell_type_exp[,-1]

cell_type_exp[1:4,1:4]

cell_type_exp=aggregate(.~cell_type,cell_type_exp,mean)

rownames(cell_type_exp)=cell_type_exp$cell_type

cell_type_exp=cell_type_exp[,-1]

cell_type_exp1=crbind2DataFrame(t(cell_type_exp))

head(cell_type_exp1)

cell_type_exp=data.frame(gene=rownames(cell_type_exp1),cell_type_exp1)

head(cell_type_exp)

colnames(cell_type_exp)=c('gene',colnames(cell_type_exp1))

write.table(cell_type_exp,'cell_type_exp.txt',sep='\t',quote = F,row.names = F)

cell_predic_results <- CIBERSORT::cibersort('cell_type_exp.txt', 'tcga_exp.txt')

save(cell_predic_results,file = 'cell_predic_results.RData')

rownames(cell_predic_results)=gsub('\\.','-',rownames(cell_predic_results))

#正常和肿瘤中比较

#normal:green ;tumor:red

vioplot_plot=function(rt,normal,tumor){

library(vioplot)

rt=rt[c(normal,tumor),]

normal=length(normal)

tumor=length(tumor)

par(las=1,mar=c(10,6,3,3))

x=c(1:ncol(rt))

y=c(1:ncol(rt))

plot(x,y,

xlim=c(0,3*ncol(rt)-1),ylim=c(min(rt),max(rt)+0.02),

main="",xlab="", ylab="Fraction",

pch=21,

col="white",

xaxt="n")

for(i in 1:ncol(rt)){

if(sd(rt[1:normal,i])==0){

rt[1,i]=0.001

}

if(sd(rt[(normal+1):(normal+tumor),i])==0){

rt[(normal+1),i]=0.001

}

normalData=rt[1:normal,i]

tumorData=rt[(normal+1):(normal+tumor),i]

vioplot(normalData,at=3*(i-1),lty=1,add = T,col = 'green')

vioplot(tumorData,at=3*(i-1)+1,lty=1,add = T,col = 'red')

#wilcoxTest=wilcox.test(normalData,tumorData)

wilcoxTest=t.test(normalData,tumorData)

p=round(wilcoxTest$p.value,3)

mx=max(c(normalData,tumorData))

lines(c(x=3*(i-1)+0.2,x=3*(i-1)+0.8),c(mx,mx))

#text(x=3*(i-1)+0.5, y=mx+0.02, labels=ifelse(p<0.05, paste0("p=",p),''), cex = 0.8)

text(x=3*(i-1)+0.5, y=mx+0.02, labels=paste0("p=",p), cex = 0.8)

text(seq(1,3*ncol(rt),3),-0.05,xpd = NA,labels=colnames(rt),cex = 1,srt = 45,pos=2)

}

}

normal=rownames(cell_predic_results)[grep('^GTEX',rownames(cell_predic_results))]

tumor=rownames(cell_predic_results)[grep('^TCGA',rownames(cell_predic_results))]

head(cell_predic_results)

dim(cell_predic_results)

cell_predic_result=cell_predic_results[,c(1:20)]

vioplot_plot(rt = cell_predic_result[c(normal,tumor),],normal = normal,tumor = tumor)

pdf('PDFs/vioplot_TvsN_cell_pre.pdf',he=7,width = 9)

vioplot_plot(rt = cell_predic_result[c(normal,tumor),],normal = normal,tumor = tumor)

dev.off()

#具有显著性的几个亚群

pre_sig_cell<-colnames(cell_predic_result)[c(2,3,5,6,7,8,9,11,13,14,15,16,17,18,19,20)]

length(pre_sig_cell)

#统计这几个亚群高低组中正常和肿瘤样本的比例

pre_sig_cell_freq<-cell_predic_result[,pre_sig_cell]

head(pre_sig_cell_freq)

pre_sig_cell_freq=apply(pre_sig_cell_freq,2,function(x){

y=ifelse(x>median(x),'High','Low')

return(y)

})

head(pre_sig_cell_freq)

pre_sig_cell_freq=crbind2DataFrame(pre_sig_cell_freq)

pre_sig_cell_freq=pre_sig_cell_freq[c(normal,tumor),]

pre_sig_cell_freq=cbind(type=c(rep('normal',length(normal)),

rep('tumor',length(tumor))),

pre_sig_cell_freq)

#B cell_1

B_cell_1_dat<-table(pre_sig_cell_freq$type,pre_sig_cell_freq$`B cell_1`)

B_cell_1_dat

B_cell_1_plot<-plotMutiBar(B_cell_1_dat,ist = T,legTitle = 'B cell_1')

B_cell_1_plot

#B cell_2

B_cell_2_dat<-table(pre_sig_cell_freq$type,pre_sig_cell_freq$`B cell_2`)

B_cell_2_dat

B_cell_2_plot<-plotMutiBar(B_cell_2_dat,ist = T,legTitle = 'B cell_2')

B_cell_2_plot

#Beta cell

Beta_cell_dat<-table(pre_sig_cell_freq$type,pre_sig_cell_freq$`Beta cell`)

Beta_cell_dat

Beta_cell_plot<-plotMutiBar(Beta_cell_dat,ist = T,legTitle = 'Beta cell')

Beta_cell_plot

#Cancer cell_1

Cancer_cell_1_dat<-table(pre_sig_cell_freq$type,pre_sig_cell_freq$`Cancer cell_1`)

Cancer_cell_1_dat

Cancer_cell_1_plot<-plotMutiBar(Cancer_cell_1_dat,ist = T,legTitle = 'Cancer cell_1')

Cancer_cell_1_plot

#Cancer cell_2

Cancer_cell_2_dat<-table(pre_sig_cell_freq$type,pre_sig_cell_freq$`Cancer cell_2`)

Cancer_cell_2_dat

Cancer_cell_2_plot<-plotMutiBar(Cancer_cell_2_dat,ist = T,legTitle = 'Cancer cell_2')

Cancer_cell_2_plot

#Cancer cell_3

Cancer_cell_3_dat<-table(pre_sig_cell_freq$type,pre_sig_cell_freq$`Cancer cell_3`)

Cancer_cell_3_dat

Cancer_cell_3_plot<-plotMutiBar(Cancer_cell_3_dat,ist = T,legTitle = 'Cancer cell_3')

Cancer_cell_3_plot

#CD1C-CD141- dendritic cell_1

CD1C__CD141__dendritic_cell_1_dat<-table(pre_sig_cell_freq$type,pre_sig_cell_freq$`CD1C-CD141- dendritic cell_1`)

CD1C__CD141__dendritic_cell_1_dat

CD1C__CD141__dendritic_cell_1_plot<-plotMutiBar(CD1C__CD141__dendritic_cell_1_dat,ist=T,

legTitle = 'CD1C-CD141- dendritic cell_1')

CD1C__CD141__dendritic_cell_1_plot

#CD1C+_B dendritic cell_1

CD1C_B_dendritic_cell_1_dat<-table(pre_sig_cell_freq$type,pre_sig_cell_freq$`CD1C+_B dendritic cell_1`)

CD1C_B_dendritic_cell_1_dat

CD1C_B_dendritic_cell_1_plot<-plotMutiBar(CD1C_B_dendritic_cell_1_dat,ist = T)

CD1C_B_dendritic_cell_1_plot

#CD1C+_B dendritic cell_3

CD1C_B_dendritic_cell_3_dat<-table(pre_sig_cell_freq$type,pre_sig_cell_freq$`CD1C+_B dendritic cell_3`)

CD1C_B_dendritic_cell_3_dat

CD1C_B_dendritic_cell_3_plot<-plotMutiBar(CD1C_B_dendritic_cell_3_dat,ist = T,

legTitle = 'CD1C+_B dendritic cell_1')

CD1C_B_dendritic_cell_3_plot

#Endothelial cell

Endothelial_cell_dat<-table(pre_sig_cell_freq$type,pre_sig_cell_freq$`Endothelial cell`)

Endothelial_cell_dat

Endothelial_cell_plot<-plotMutiBar(Endothelial_cell_dat,ist = T,legTitle = 'Endothelial cell')

Endothelial_cell_plot

#Fibroblast_1

Fibroblast_1_dat<-table(pre_sig_cell_freq$type,pre_sig_cell_freq$Fibroblast_1)

Fibroblast_1_dat

Fibroblast_1_plot=plotMutiBar(Fibroblast_1_dat,ist = T,legTitle = 'Fibroblast_1')

Fibroblast_1_plot

#Fibroblast_2

Fibroblast_2_dat<-table(pre_sig_cell_freq$type,pre_sig_cell_freq$Fibroblast_2)

Fibroblast_2_dat

Fibroblast_2_plot<-plotMutiBar(Fibroblast_2_dat,ist = T,legTitle = 'Fibroblast_2')

Fibroblast_2_plot

#Myeloid dendritic cell

Myeloid_dendritic_cell_dat<-table(pre_sig_cell_freq$type,pre_sig_cell_freq$`Myeloid dendritic cell`)

Myeloid_dendritic_cell_dat

Myeloid_dendritic_cell_plot<-plotMutiBar(Myeloid_dendritic_cell_dat,ist = T,legTitle = 'Myeloid dendritic cell')

Myeloid_dendritic_cell_plot

#Natural killer cell_1

Natural_killer_cell_1_dat<-table(pre_sig_cell_freq$type,pre_sig_cell_freq$`Natural killer cell_1`)

Natural_killer_cell_1_dat

Natural_killer_cell_1_plot<-plotMutiBar(Natural_killer_cell_1_dat,ist = T,

legTitle = 'Natural killer cell_1')

Natural_killer_cell_1_plot

#Natural killer cell_2

Natural_killer_cell_2_dat<-table(pre_sig_cell_freq$type,pre_sig_cell_freq$`Natural killer cell_2`)

Natural_killer_cell_2_dat

Natural_killer_cell_2_plot<-plotMutiBar(Natural_killer_cell_2_dat,ist = T,

legTitle = 'Natural killer cell_2')

Natural_killer_cell_2_plot

#Plasmacytoid dendritic cell

Plasmacytoid_dendritic_cell_dat<-table(pre_sig_cell_freq$type,pre_sig_cell_freq$`Plasmacytoid dendritic cell`)

Plasmacytoid_dendritic_cell_dat

Plasmacytoid_dendritic_cell_plot<-plotMutiBar(Plasmacytoid_dendritic_cell_dat,ist = T,

legTitle = 'Plasmacytoid dendritic cell')

Plasmacytoid_dendritic_cell_plot

#merge

cell_pre_plot<-mg_merge_plot(B_cell_1_plot,B_cell_2_plot,Beta_cell_plot,Cancer_cell_1_plot,

Cancer_cell_2_plot,Cancer_cell_3_plot,

CD1C__CD141__dendritic_cell_1_plot,CD1C_B_dendritic_cell_1_plot,

CD1C_B_dendritic_cell_3_plot,Endothelial_cell_plot,Fibroblast_1_plot,

Fibroblast_2_plot,Myeloid_dendritic_cell_plot,

Natural_killer_cell_1_plot,Natural_killer_cell_2_plot,

Plasmacytoid_dendritic_cell_plot,nrow=4,ncol=4)

cell_pre_plot

savePDF(filename = 'PDFs/cell_pre_plot.pdf',plot = cell_pre_plot,he=15,wi=15)

#有意义的亚群

pre_sig_cell1<-pre_sig_cell

length(pre_sig_cell1)

#肿瘤细胞单独做预测：

write.table(data.frame(GeneSymbol=rownames(merge_tcga_nor),merge_tcga_nor[,sam_T]),file = 'tcga_exp_t.txt',row.names = F,quote = F,sep='\t')

#cell__T_predic_results <- CIBERSORT::cibersort('cell_type_exp.txt', 'tcga_exp_t.txt')

#save(cell__T_predic_results,file = 'cell__T_predic_results.RData')

rownames(cell__T_predic_results)=gsub('\\.','-',rownames(cell__T_predic_results))

head(cell__T_predic_results)

cell__T_predic_result=cell__T_predic_results[,pre_sig_cell1]

head(cell__T_predic_result)

cell__T_predic_result1=cell__T_predic_result

cell__T_predic_result1=apply(cell__T_predic_result1,2,function(x){

y=ifelse(x>median(x),'High','Low')

return(y)

})

cell__T_predic_result1=crbind2DataFrame(cell__T_predic_result1)

head(cell__T_predic_result1)

#合并生存时间和生存状态

head(tcga_cli)

tcga_cli_celltype<-merge(tcga_cli,

data.frame(Samples=rownames(cell__T_predic_result1),

cell__T_predic_result1),

by='Samples')

colnames(tcga_cli_celltype)=c(colnames(tcga_cli),colnames(cell__T_predic_result1))

head(tcga_cli_celltype)

tcga_cli_celltype=crbind2DataFrame(tcga_cli_celltype)

tcga_cli_celltype=tcga_cli_celltype[which(tcga_cli_celltype$OS.time>0),]

#KM曲线

#B cell_1

B_cell_1_km<-ggplotKMCox(data.frame(tcga_cli_celltype$OS.time/365,

tcga_cli_celltype$OS,

tcga_cli_celltype$`B cell_1`),

title = 'B cell_1',

labs = c('High','Low'),

add_text = '')

B_cell_1_km

#B cell_2

B_cell_2_km<-ggplotKMCox(data.frame(tcga_cli_celltype$OS.time/365,

tcga_cli_celltype$OS,

tcga_cli_celltype$`B cell_2`),

title = 'B cell_2',

labs = c('High','Low'),

add_text = '')

B_cell_2_km

#Beta cell

Beta_cell_km<-ggplotKMCox(data.frame(tcga_cli_celltype$OS.time/365,

tcga_cli_celltype$OS,

tcga_cli_celltype$`Beta cell`),

title = 'Beta cell',

labs = c('High','Low'),

add_text = '')

Beta_cell_km

#Cancer cell_1

Cancer_cell_1_km<-ggplotKMCox(data.frame(tcga_cli_celltype$OS.time/365,

tcga_cli_celltype$OS,

tcga_cli_celltype$`Cancer cell_1`),

title = 'Cancer cell_1',

labs = c('High','Low'),

add_text = '')

Cancer_cell_1_km

#Cancer cell_2

Cancer_cell_2_km<-ggplotKMCox(data.frame(tcga_cli_celltype$OS.time/365,

tcga_cli_celltype$OS,

tcga_cli_celltype$`Cancer cell_2`),

title = 'Cancer cell_2',

labs = c('High','Low'),

add_text = '')

Cancer_cell_2_km

#Cancer cell_3

Cancer_cell_3_km<-ggplotKMCox(data.frame(tcga_cli_celltype$OS.time/365,

tcga_cli_celltype$OS,

tcga_cli_celltype$`Cancer cell_3`),

title = 'Cancer cell_3',

labs = c('High','Low'),

add_text = '')

Cancer_cell_3_km

#CD1C-CD141- dendritic cell_1

CD1C__CD141__dendritic_cell_1_km<-ggplotKMCox(data.frame(tcga_cli_celltype$OS.time/365,

tcga_cli_celltype$OS,

tcga_cli_celltype$`CD1C-CD141- dendritic cell_1`),

title = 'CD1C-CD141- dendritic cell_1',

labs = c('High','Low'),

add_text = '')

CD1C__CD141__dendritic_cell_1_km

#CD1C+_B dendritic cell_1

CD1C_B_dendritic_cell_1_km<-ggplotKMCox(data.frame(tcga_cli_celltype$OS.time/365,

tcga_cli_celltype$OS,

tcga_cli_celltype$`CD1C+_B dendritic cell_1`),

title = 'CD1C+_B dendritic cell_1',

labs = c('High','Low'),

add_text = '')

CD1C_B_dendritic_cell_1_km

#CD1C+_B dendritic cell_3

CD1C_B_dendritic_cell_3_km<-ggplotKMCox(data.frame(tcga_cli_celltype$OS.time/365,

tcga_cli_celltype$OS,

tcga_cli_celltype$`CD1C+_B dendritic cell_3`),

title = 'CD1C+_B dendritic cell_3',

labs = c('High','Low'),

add_text = '')

CD1C_B_dendritic_cell_3_km

#Endothelial cell

Endothelial_cell_km<-ggplotKMCox(data.frame(tcga_cli_celltype$OS.time/365,

tcga_cli_celltype$OS,

tcga_cli_celltype$`Endothelial cell`),

title = 'Endothelial cell',

labs = c('High','Low'),

add_text = '')

Endothelial_cell_km

#Fibroblast_1

Fibroblast_1_km<-ggplotKMCox(data.frame(tcga_cli_celltype$OS.time/365,

tcga_cli_celltype$OS,

tcga_cli_celltype$Fibroblast_1),

title = 'Fibroblast_1',

labs = c('High','Low'),

add_text = '')

Fibroblast_1_km

#Fibroblast_2

Fibroblast_2_km<-ggplotKMCox(data.frame(tcga_cli_celltype$OS.time/365,

tcga_cli_celltype$OS,

tcga_cli_celltype$Fibroblast_2),

title = 'Fibroblast_2',

labs = c('High','Low'),

add_text = '')

Fibroblast_2_km

#Myeloid dendritic cell

Myeloid_dendritic_cell_km<-ggplotKMCox(data.frame(tcga_cli_celltype$OS.time/365,

tcga_cli_celltype$OS,

tcga_cli_celltype$`Myeloid dendritic cell`),

title = 'Myeloid dendritic cell',

labs = c('High','Low'),

add_text = '')

Myeloid_dendritic_cell_km

#Natural killer cell_1

Natural_killer_cell_1_km<-ggplotKMCox(data.frame(tcga_cli_celltype$OS.time/365,

tcga_cli_celltype$OS,

tcga_cli_celltype$`Natural killer cell_1`),

title = 'Natural killer cell_1',

labs = c('High','Low'),

add_text = '')

Natural_killer_cell_1_km

#Natural killer cell_2

Natural_killer_cell_2_km<-ggplotKMCox(data.frame(tcga_cli_celltype$OS.time/365,

tcga_cli_celltype$OS,

tcga_cli_celltype$`Natural killer cell_2`),

title = 'Natural killer cell_2',

labs = c('High','Low'),

add_text = '')

Natural_killer_cell_2_km

#Plasmacytoid dendritic cell

Plasmacytoid_dendritic_cell_km<-ggplotKMCox(data.frame(tcga_cli_celltype$OS.time/365,

tcga_cli_celltype$OS,

tcga_cli_celltype$`Plasmacytoid dendritic cell`),

title = 'Plasmacytoid dendritic cell',

labs = c('High','Low'),

add_text = '')

Plasmacytoid_dendritic_cell_km

#merge

cell_pre_km<-mg_merge_plot(B_cell_1_km,B_cell_2_km,Beta_cell_km,Cancer_cell_1_km,Cancer_cell_2_km,

Cancer_cell_3_km,CD1C__CD141__dendritic_cell_1_km,

CD1C_B_dendritic_cell_1_km,CD1C_B_dendritic_cell_3_km,

Endothelial_cell_km,Fibroblast_1_km,Fibroblast_2_km,

Myeloid_dendritic_cell_km,Natural_killer_cell_1_km,

Natural_killer_cell_2_km,Plasmacytoid_dendritic_cell_km,

ncol=4,nrow=4)

cell_pre_km

savePDF(filename = 'PDFs/cell_pre_KM.pdf',plot = cell_pre_km,he=17,wi=17)

#取出4,10,18,16四个亚群的marker基因，并进行单因素cox分析

c4_marker_gene<-unique(sce.markers[which(sce.markers$cluster==4),]$gene)

c10_marker_gene<-unique(sce.markers[which(sce.markers$cluster==10),]$gene)

c18_marker_gene<-unique(sce.markers[which(sce.markers$cluster==18),]$gene)

c16_marker_gene<-unique(sce.markers[which(sce.markers$cluster==16),]$gene)

#过滤基因

c4_marker_gene<-setdiff(c4_marker_gene,unique(c(c10_marker_gene,c18_marker_gene,c16_marker_gene)))

length(c4_marker_gene)

c10_marker_gene<-setdiff(c10_marker_gene,unique(c(c4_marker_gene,c18_marker_gene,c16_marker_gene)))

length(c10_marker_gene)

c18_marker_gene<-setdiff(c18_marker_gene,unique(c(c4_marker_gene,c10_marker_gene,c16_marker_gene)))

length(c18_marker_gene)

c16_marker_gene<-setdiff(c16_marker_gene,unique(c(c4_marker_gene,c10_marker_gene,c18_marker_gene)))

length(c16_marker_gene)

#TCGA

coxFun <- function(dat){

library(survival)

colnames(dat)=c('time','status','gene')

fmla=as.formula("Surv(time,status)~gene")

cox=coxph(fmla,data=dat)

p=summary(cox)[[7]][5]

result=c(p,summary(cox)[[8]][1],summary(cox)[[8]][3],summary(cox)[[8]][4])

return(result)

}

marker_cox<-function(gene,tcga_dat_T,tcga_cli){

tcga_dat_T_marker<-tcga_dat_T[intersect(gene,rownames(tcga_dat_T)),]

tcga_dat_T_marker<-merge(tcga_cli[,c("Samples","OS.time","OS")],

data.frame(Samples=colnames(tcga_dat_T_marker),

t(tcga_dat_T_marker)),

by='Samples')

rownames(tcga_dat_T_marker)=tcga_dat_T_marker$Samples

tcga_dat_T_marker=tcga_dat_T_marker[,-1]

marker.cox <- t(apply(tcga_dat_T_marker[,3:c(ncol(tcga_dat_T_marker))],2,function(x){

vl=as.numeric(x)

tm=tcga_dat_T_marker$OS.time

ev=tcga_dat_T_marker$OS

#ev=ifelse(ev=='Alive',0,1)

dat=data.frame(tm,ev,vl)[which(tm > 0 & !is.na(vl)),]

return(coxFun(dat))

}))

colnames(marker.cox)=c('p.value','HR','Low 95%CI','High 95%CI')

marker.cox=crbind2DataFrame(marker.cox)

return(marker.cox)

}

C4_cox=marker_cox(gene = c4_marker_gene,tcga_dat_T,tcga_cli)

C10_cox=marker_cox(gene = c10_marker_gene,tcga_dat_T,tcga_cli)

C18_cox=marker_cox(gene = c18_marker_gene,tcga_dat_T,tcga_cli)

C16_cox=marker_cox(gene = c16_marker_gene,tcga_dat_T,tcga_cli)

table(C4_cox$p.value<0.001)

table(C10_cox$p.value<0.001)

table(C18_cox$p.value<0.001)

table(C16_cox$p.value<0.001)

C4_cox.sig<-C4_cox[which(C4_cox$p.value<0.001),]

C10_cox.sig<-C10_cox[which(C10_cox$p.value<0.001),]

C18_cox.sig<-C18_cox[which(C18_cox$p.value<0.001),]

C16_cox.sig<-C16_cox[which(C16_cox$p.value<0.001),]

cox_sig_gene<-c(rownames(C4_cox.sig),rownames(C10_cox.sig),rownames(C18_cox.sig),rownames(C16_cox.sig))

con_merge<-rbind(cbind(gene=rownames(C4_cox.sig),C4_cox.sig,marker='B cell'),

cbind(gene=rownames(C10_cox.sig),C10_cox.sig,marker='B cell'),

cbind(gene=rownames(C18_cox.sig),C18_cox.sig,marker='cancer cell'),

cbind(gene=rownames(C16_cox.sig),C16_cox.sig,marker='CD1C+_B dendritic cell'))

write.table(con_merge,'results/files/cox_sig_markers.txt',quote = F,row.names = F,sep='\t')

#提取基因做相关性分析

tcga_sig_cox<-tcga_dat_T[cox_sig_gene,intersect(colnames(tcga_dat_T),tcga_cli$Samples)]

tcga_sig_cox[1:4,1:4]

sig_cox_cor=Hmisc::rcorr(as.matrix(t(tcga_sig_cox)))

cox_cor=reshape2::melt(sig_cox_cor$r)

colnames(cox_cor)=c('gene1','gene2','cor')

cox_cor=cox_cor[cox_cor$cor!=1,]

cox_p=reshape2::melt(sig_cox_cor$P)

colnames(cox_p)=c('gene1','gene2','p')

cox_p=na.omit(cox_p)

cox_result<-merge(cox_cor,cox_p,by=c('gene1','gene2'))

head(cox_result)

table(abs(cox_result$cor>0.9),cox_result$p<0.001)

cox_result_fit<-unique(cox_result[which(abs(cox_result$cor>0.9) & cox_result$p<0.001),])

dim(cox_result_fit)

write.table(cox_result_fit,'results/files/cox_result.txt',quote = F,sep='\t',row.names = F)

#绘制网络图的文件

gene_hub<-cox_result_fit[,c(1,2)]

con_merge1=con_merge[,c("gene","marker")]

colnames(con_merge1)=c('gene1','gene2')

gene_hub<-rbind(gene_hub,con_merge1)

gene_hub=unique(gene_hub)

write.table(gene_hub,'files/gene_hub.txt',quote = F,row.names = F,sep='\t')

#lab文件

gene_lab<-rbind(data.frame(gene=con_merge$gene,type='gene'),

data.frame(gene=unique(con_merge$marker),type='cell_type'))

write.table(gene_lab,'files/gene_lab.txt',sep='\t',quote = F,row.names = F)

# BUB1 基因

tcga_sig_cox

BUB1_data<-data.frame(OS.time=tcga_cli[tcga_cli$Samples %in% colnames(tcga_sig_cox),]$OS.time/365,

OS=tcga_cli[tcga_cli$Samples %in% colnames(tcga_sig_cox),]$OS,

t(tcga_sig_cox['BUB1',]))

BUB1_data$BUB1=ifelse(BUB1_data$BUB1>mean(BUB1_data$BUB1),'High','Low')

BUB1_km<-ggplotKMCox(BUB1_data,title = 'BUB1 expression',

labs = c('High','Low'),

add_text = '')

BUB1_km

savePDF(plot = BUB1_km,filename = 'PDFs/BUB1_km.pdf',he=7,wi=7)

load('sce2.RData')

sce_count<-as.matrix(sce@assays$RNA@counts)

sce_count=as.data.frame(sce_count['BUB1',])

cell_group<-sce@meta.data

sce_count1<-cbind(sce_count[rownames(cell_group),],cell_group$Group2)

sce_count1=crbind2DataFrame(sce_count1)

head(sce_count1)

sce_count1=sce_count1[which(sce_count1$V1>0),]

VlnPlot(object = sce,

features = c('BUB1'),

pt.size = 0)+facet_grid(.~Sample)

Idents(sce) <- "Group1"

unique(sce$Group1)

IPMN_umap<-FeaturePlot(subset(sce, idents = c("IPMN")),

features = c('BUB1'),

reduction = "umap",pt.size = 0.5)+ ggtitle('IPMN sample')

PASC_umap<-FeaturePlot(subset(sce, idents = c("PASC")),

features = c('BUB1'),

reduction = "umap",pt.size = 0.5)+ ggtitle('PASC sample')

normal_umap<-FeaturePlot(subset(sce, idents = c("Normal")),

features = c('BUB1'),

reduction = "umap",pt.size = 0.5)+ ggtitle('Normal sample')

normal_umap

sample_umap<-mg_merge_plot(IPMN_umap,PASC_umap,normal_umap,labels = c('C','D','E'),nrow=1,ncol=3)

sample_umap

savePDF(filename = 'PDFs/sample_umap.pdf',plot = sample_umap,width =15,height = 5)

rm(sce)

#BUB1高低表达组在临床特征上的表现####

BUB1_dat<-merge(tcga_cli,

data.frame(Samples=colnames(tcga_sig_cox),

BUB1=t(tcga_sig_cox['BUB1',])),

by='Samples')

head(BUB1_dat)

BUB1_dat$BUB1=ifelse(BUB1_dat$BUB1>median(BUB1_dat$BUB1),'High','Low')

#Event

Event_compare_dat <- table(BUB1_dat$OS, BUB1_dat$BUB1)

Event_compare_dat

Event_compare <- plotMutiBar(Event_compare_dat, legTitle='Event', showValue = T)

Event_compare

#T

BUB1_dat$T.Stage[BUB1_dat$T.Stage == 'T1' | BUB1_dat$T.Stage == 'T2'] <- 'T1+T2'

BUB1_dat$T.Stage[BUB1_dat$T.Stage == 'T3' | BUB1_dat$T.Stage == 'T4'] <- 'T3+T4'

T_compare_dat <- table(BUB1_dat$T.Stage, BUB1_dat$BUB1)

T_compare_dat

T_compare <- plotMutiBar(T_compare_dat[-c(1,4),], legTitle='T.Stage', showValue = T)

T_compare

#N

BUB1_dat$N.Stage=gsub('b','',BUB1_dat$N.Stage)

N_compare_dat <- table(BUB1_dat$N.Stage, BUB1_dat$BUB1)

N_compare_dat

N_compare <- plotMutiBar(N_compare_dat[-c(1,4),], legTitle='N.Stage', showValue = T)

N_compare

#M

M_compare_dat <- table(BUB1_dat$M.Stage, BUB1_dat$BUB1)

M_compare_dat

M_compare <- plotMutiBar(M_compare_dat[-3,], legTitle='M.Stage', showValue = T)

M_compare

#ICGC数据集验证BUB1基因的预后###

#AU

# load('origin_datas/ICGC/paad_exp_au.RData')

# paad_exp_au[1:4,1:4]

# paad_au_cli=read.delim('origin_datas/ICGC/donor.PACA-AU.tsv',sep='\t',header = T,check.names = F)

# paad_au_cli=data.frame(Samples=paad_au_cli$icgc_donor_id,

# OS=paad_au_cli$donor_vital_status,

# OS.time=paad_au_cli$donor_survival_time)

# rownames(paad_au_cli)=paad_au_cli$Samples

# paad_au_cli$OS=gsub('deceased',1,paad_au_cli$OS)

# paad_au_cli$OS=gsub('alive',0,paad_au_cli$OS)

# paad_au_cli=na.omit(paad_au_cli)

# paad_au_cli=paad_au_cli[which(paad_au_cli$OS.time>0),]

# paad_au_cli=crbind2DataFrame(paad_au_cli)

#

# paad_exp_au=merge(data.frame(gene_id=gene_type$ENSGID,

# symbol=gene_type$SYMBOL),

# paad_exp_au,by='gene_id')

#

# paad_exp_au=paad_exp_au[,-1]

# paad_exp_au[1:4,1:4]

# paad_exp_au=aggregate(.~symbol,paad_exp_au,mean)

#

# rownames(paad_exp_au)=paad_exp_au$symbol

# paad_exp_au=paad_exp_au[,-1]

# com_icga_au_sample=intersect(colnames(paad_exp_au),paad_au_cli$Samples)

# paad_exp_au_gene<-cbind(paad_au_cli[paad_au_cli$Samples %in% com_icga_au_sample,],t(paad_exp_au['BUB1',com_icga_au_sample]))

# paad_exp_au_gene$BUB1=ifelse(paad_exp_au_gene$BUB1>mean(paad_exp_au_gene$BUB1),'High','Low')

#

# # library(survminer)

# # res.cut <- surv_cutpoint(paad_exp_au_gene,

# # time = "OS.time",

# # event = "OS",

# # variables = c("BUB1"))

# # res.cut$cutpoint$cutpoint

# # paad_exp_au_gene$BUB1=ifelse(paad_exp_au_gene$BUB1>res.cut$cutpoint$cutpoint,'High','Low')

# BUB1_km_au<-ggplotKMCox(data.frame(paad_exp_au_gene$OS.time/365,

# paad_exp_au_gene$OS,

# paad_exp_au_gene$BUB1),

# title = 'BUB1 expression',

# labs = c('High','Low'),

# add_text = '')

# BUB1_km_au

#

#

# #CA####

# load('origin_datas/ICGC/paad_exp_ca.RData')

# paad_exp_ca[1:4,1:4]

# paad_ca_cli=read.delim('origin_datas/ICGC/donor.PACA-CA.tsv',sep='\t',header = T,check.names = F)

# paad_ca_cli=data.frame(Samples=paad_ca_cli$icgc_donor_id,

# OS=paad_ca_cli$donor_vital_status,

# OS.time=paad_ca_cli$donor_survival_time)

# rownames(paad_ca_cli)=paad_ca_cli$Samples

# paad_ca_cli$OS=gsub('deceased',1,paad_ca_cli$OS)

# paad_ca_cli$OS=gsub('alive',0,paad_ca_cli$OS)

# paad_ca_cli=na.omit(paad_ca_cli)

# paad_ca_cli=paad_ca_cli[which(paad_ca_cli$OS.time>0),]

# paad_ca_cli=crbind2DataFrame(paad_ca_cli)

#

# paad_exp_ca=merge(data.frame(gene_id=gene_type$ENSGID,

# symbol=gene_type$SYMBOL),

# paad_exp_ca,by='gene_id')

#

# paad_exp_ca=paad_exp_ca[,-1]

# paad_exp_ca[1:4,1:4]

# paad_exp_ca=aggregate(.~symbol,paad_exp_ca,mean)

#

# rownames(paad_exp_ca)=paad_exp_ca$symbol

# paad_exp_ca=paad_exp_ca[,-1]

# com_icga_ca_sample=intersect(colnames(paad_exp_ca),paad_ca_cli$Samples)

# paad_exp_ca_gene<-cbind(paad_ca_cli[paad_ca_cli$Samples %in% com_icga_ca_sample,],t(paad_exp_ca['BUB1',com_icga_ca_sample]))

#

# paad_exp_ca_gene$BUB1=ifelse(paad_exp_ca_gene$BUB1>mean(paad_exp_ca_gene$BUB1),'High','Low')

#

# # library(survminer)

# # res.cut <- surv_cutpoint(paad_exp_ca_gene,

# # time = "OS.time",

# # event = "OS",

# # variables = c("BUB1"))

# # res.cut$cutpoint$cutpoint

# # paad_exp_ca_gene$BUB1=ifelse(paad_exp_ca_gene$BUB1>res.cut$cutpoint$cutpoint,'High','Low')

#

# BUB1_km_ca<-ggplotKMCox(data.frame(paad_exp_ca_gene$OS.time/365,

# paad_exp_ca_gene$OS,

# paad_exp_ca_gene$BUB1),

# title = 'BUB1 expression',

# labs = c('High','Low'),

# add_text = '')

# BUB1_km_ca

#合并ICGC数据集

# paad_exp_icgc<-rbind(cbind(paad_ca_cli[paad_ca_cli$Samples %in% com_icga_ca_sample,],t(paad_exp_ca['BUB1',com_icga_ca_sample])),

# cbind(paad_au_cli[paad_au_cli$Samples %in% com_icga_au_sample,],t(paad_exp_au['BUB1',com_icga_au_sample])))

#

# paad_exp_icgc$BUB1=ifelse(paad_exp_icgc$BUB1>mean(paad_exp_icgc$BUB1),'High','Low')

#

# # res.cut <- surv_cutpoint(paad_exp_icgc,

# # time = "OS.time",

# # event = "OS",

# # variables = c("BUB1"))

# # res.cut$cutpoint$cutpoint

# # paad_exp_icgc$BUB1=ifelse(paad_exp_icgc$BUB1>res.cut$cutpoint$cutpoint,'High','Low')

# BUB1_km_icgc<-ggplotKMCox(data.frame(paad_exp_icgc$OS.time/365,

# paad_exp_icgc$OS,

# paad_exp_icgc$BUB1),

# title = 'BUB1 expression',

# labs = c('High','Low'),

# add_text = '')

# BUB1_km_icgc

#GEO数据集验证####

paad_data=mg_data_hub_PAAD(dtype = 'mEXP')

names(paad_data)

# #GSE102238

# GSE102238_exp=paad_data$GSE102238$EXP

# GSE102238_cli=paad_data$GSE102238$CLINI

# GSE102238_OS=paad_data$GSE102238$OS

# #提取肿瘤样本

# GSE102238_cli=merge(data.frame(Samples=rownames(GSE102238_cli),GSE102238_cli),

# data.frame(Samples=rownames(GSE102238_OS),GSE102238_OS),

# by='Samples')

#

#

# GSE102238_cli=GSE102238_cli[which(GSE102238_cli$TISSUE=='Tumor'),]

#

# colnames(GSE102238_cli)

# colnames(GSE102238_cli)=c('Samples','T.stage','N.Stage','M.Stage','gender','Tissue','Disease','OS.time','OS')

# rownames(GSE102238_cli)=GSE102238_cli$Samples

# GSE102238_cli=GSE102238_cli[which(GSE102238_cli$OS.time!='NA'),]

# GSE102238_exp=GSE102238_exp[,GSE102238_cli$Samples]

# boxplot(GSE102238_exp[1:10],las=2)

# GSE102238_dat<-cbind(OS.time=GSE102238_cli$OS.time,

# OS=GSE102238_cli$OS,

# t(GSE102238_exp['BUB1',]))

# GSE102238_dat=crbind2DataFrame(GSE102238_dat)

# GSE102238_dat$BUB1=ifelse(GSE102238_dat$BUB1>mean(GSE102238_dat$BUB1),'High','Low')

# # res.cut <- surv_cutpoint(GSE102238_dat,

# # time = "OS.time",

# # event = "OS",

# # variables = c("BUB1"))

# # GSE102238_dat$BUB1=ifelse(GSE102238_dat$BUB1>res.cut$cutpoint$cutpoint,'High','Low')

# GSE102238_km<-ggplotKMCox(data.frame(GSE102238_dat$OS.time,

# GSE102238_dat$OS,

# GSE102238_dat$BUB1),

# title = 'BUB1 expression',

# labs = c('High','Low'),

# add_text = '')

# GSE102238_km

#GSE21501

# GSE21501_exp=paad_data$GSE21501$EXP

# GSE21501_cli=paad_data$GSE21501$CLINI

# GSE21501_OS=paad_data$GSE21501$OS

# #提取肿瘤样本

# GSE21501_cli=merge(data.frame(Samples=rownames(GSE21501_cli),GSE21501_cli),

# data.frame(Samples=rownames(GSE21501_OS),GSE21501_OS),

# by='Samples')

#

# GSE21501_cli=na.omit(GSE21501_cli)

#

# colnames(GSE21501_cli)

# colnames(GSE21501_cli)=c('Samples','T.stage','Stage','OS.time','OS')

# rownames(GSE21501_cli)=GSE21501_cli$Samples

#

# GSE21501_cli$Stage=gsub('Stage ','',GSE21501_cli$Stage)

# rownames(GSE21501_cli)=GSE21501_cli$Samples

# GSE21501_exp=GSE21501_exp[,as.character(GSE21501_cli$Samples)]

# dim(GSE21501_exp)

# GSE21501_exp=log2(GSE21501_exp+1)

#

# GSE21501_dat<-cbind(OS.time=GSE21501_cli$OS.time,

# OS=GSE21501_cli$OS,

# BUB1=as.numeric(t(GSE21501_exp['BUB1',])))

# GSE21501_dat=crbind2DataFrame(GSE21501_dat)

# GSE21501_dat$BUB1=ifelse(GSE21501_dat$BUB1>mean(GSE21501_dat$BUB1),'High','Low')

# # res.cut <- surv_cutpoint(GSE21501_dat,

# # time = "OS.time",

# # event = "OS",

# # variables = c("BUB1"))

# # GSE21501_dat$BUB1=ifelse(GSE21501_dat$BUB1>res.cut$cutpoint$cutpoint,'High','Low')

# GSE21501_km<-ggplotKMCox(data.frame(GSE21501_dat$OS.time,

# GSE21501_dat$OS,

# GSE21501_dat$BUB1),

# title = 'BUB1 expression',

# labs = c('High','Low'),

# add_text = '')

# GSE21501_km

#GSE28735

GSE28735_exp=paad_data$GSE28735$EXP

GSE28735_cli=paad_data$GSE28735$CLINI

GSE28735_OS=paad_data$GSE28735$OS

#提取肿瘤样本

GSE28735_cli=merge(data.frame(Samples=rownames(GSE28735_cli),GSE28735_cli),

data.frame(Samples=rownames(GSE28735_OS),GSE28735_OS),

by='Samples')

GSE28735_cli=GSE28735_cli[which(GSE28735_cli$TISSUE=='Tumor'),]

GSE28735_cli=GSE28735_cli[which(GSE28735_cli$OS !='NA'),]

colnames(GSE28735_cli)

colnames(GSE28735_cli)=c('Samples','Tissue','OS.time','OS')

rownames(GSE28735_cli)=GSE28735_cli$Samples

GSE28735_exp=GSE28735_exp[,GSE28735_cli$Samples]

GSE28735_dat<-cbind(OS.time=GSE28735_cli$OS.time,

OS=GSE28735_cli$OS,

BUB1=as.numeric(t(GSE28735_exp['BUB1',])))

GSE28735_dat=crbind2DataFrame(GSE28735_dat)

GSE28735_dat$BUB1=ifelse(GSE28735_dat$BUB1>mean(GSE28735_dat$BUB1),'High','Low')

# res.cut <- surv_cutpoint(GSE28735_dat,

# time = "OS.time",

# event = "OS",

# variables = c("BUB1"))

#GSE28735_dat$BUB1=ifelse(GSE28735_dat$BUB1>res.cut$cutpoint$cutpoint,'High','Low')

GSE28735_km<-ggplotKMCox(data.frame(GSE28735_dat$OS.time,

GSE28735_dat$OS,

GSE28735_dat$BUB1),

title = 'BUB1 expression',

labs = c('High','Low'),

add_text = '')

GSE28735_km

#GSE57495

# GSE57495_exp=paad_data$GSE57495$EXP

# GSE57495_cli=paad_data$GSE57495$CLINI

# GSE57495_OS=paad_data$GSE57495$OS

# #提取肿瘤样本

# GSE57495_cli=merge(data.frame(Samples=rownames(GSE57495_cli),GSE57495_cli),

# data.frame(Samples=rownames(GSE57495_OS),GSE57495_OS),

# by='Samples')

#

# colnames(GSE57495_cli)

# colnames(GSE57495_cli)=c('Samples','Stage','OS.time','OS')

# GSE57495_cli$Stage=gsub('Stage ','',GSE57495_cli$Stage)

# rownames(GSE57495_cli)=GSE57495_cli$Samples

# GSE57495_exp=GSE57495_exp[,GSE57495_cli$Samples]

#

# GSE57495_dat<-cbind(OS.time=GSE57495_cli$OS.time,

# OS=GSE57495_cli$OS,

# BUB1=as.numeric(t(GSE57495_exp['BUB1',])))

# GSE57495_dat=crbind2DataFrame(GSE57495_dat)

# GSE57495_dat$BUB1=ifelse(GSE57495_dat$BUB1>median(GSE57495_dat$BUB1),'High','Low')

# # res.cut <- surv_cutpoint(GSE57495_dat,

# # time = "OS.time",

# # event = "OS",

# # variables = c("BUB1"))

# # GSE57495_dat$BUB1=ifelse(GSE57495_dat$BUB1>res.cut$cutpoint$cutpoint,'High','Low')

# GSE57495_km<-ggplotKMCox(data.frame(GSE57495_dat$OS.time,

# GSE57495_dat$OS,

# GSE57495_dat$BUB1),

# title = 'BUB1 expression',

# labs = c('High','Low'),

# add_text = '')

# GSE57495_km

#GSE62452

GSE62452_exp=paad_data$GSE62452$EXP

GSE62452_cli=paad_data$GSE62452$CLINI

GSE62452_OS=paad_data$GSE62452$OS

#提取肿瘤样本

GSE62452_cli=merge(data.frame(Samples=rownames(GSE62452_cli),GSE62452_cli),

data.frame(Samples=rownames(GSE62452_OS),GSE62452_OS),

by='Samples')

colnames(GSE62452_cli)

colnames(GSE62452_cli)=c('Samples','Stage','Grade','Tissue','OS.time','OS')

GSE62452_cli=GSE62452_cli[which(GSE62452_cli$Tissue=='Tumor'),]

GSE62452_cli$Stage=gsub('Stage ','',GSE62452_cli$Stage)

GSE62452_cli=na.omit(GSE62452_cli)

rownames(GSE62452_cli)=GSE62452_cli$Samples

GSE62452_exp=GSE62452_exp[,GSE62452_cli$Samples]

GSE62452_dat<-cbind(OS.time=GSE62452_cli$OS.time,

OS=GSE62452_cli$OS,

BUB1=as.numeric(t(GSE62452_exp['BUB1',])))

GSE62452_dat=crbind2DataFrame(GSE62452_dat)

GSE62452_dat$BUB1=ifelse(GSE62452_dat$BUB1>mean(GSE62452_dat$BUB1),'High','Low')

# res.cut <- surv_cutpoint(GSE62452_dat,

# time = "OS.time",

# event = "OS",

# variables = c("BUB1"))

# GSE62452_dat$BUB1=ifelse(GSE62452_dat$BUB1>res.cut$cutpoint$cutpoint,'High','Low')

GSE62452_km<-ggplotKMCox(data.frame(GSE62452_dat$OS.time,

GSE62452_dat$OS,

GSE62452_dat$BUB1),

title = 'BUB1 expression',

labs = c('High','Low'),

add_text = '')

GSE62452_km

# #GSE85916

# GSE85916_exp=paad_data$GSE85916$EXP

# #GSE85916_cli=paad_data$GSE85916$CLINI

# GSE85916_OS=paad_data$GSE85916$OS

# #提取肿瘤样本

# GSE85916_cli=data.frame(Samples=rownames(GSE85916_OS),

# GSE85916_OS)

# colnames(GSE85916_cli)

# colnames(GSE85916_cli)=c('Samples','OS.time','OS')

# rownames(GSE85916_cli)=GSE85916_cli$Samples

#

# GSE85916_exp=GSE85916_exp[,GSE85916_cli$Samples]

#

# GSE85916_dat<-cbind(OS.time=GSE85916_cli$OS.time,

# OS=GSE85916_cli$OS,

# BUB1=as.numeric(t(GSE85916_exp['BUB1',])))

# GSE85916_dat=crbind2DataFrame(GSE85916_dat)

# GSE85916_dat$BUB1=ifelse(GSE85916_dat$BUB1>median(GSE85916_dat$BUB1),'High','Low')

# # res.cut <- surv_cutpoint(GSE85916_dat,

# # time = "OS.time",

# # event = "OS",

# # variables = c("BUB1"))

# # GSE85916_dat$BUB1=ifelse(GSE85916_dat$BUB1>res.cut$cutpoint$cutpoint,'High','Low')

# GSE85916_km<-ggplotKMCox(data.frame(GSE85916_dat$OS.time,

# GSE85916_dat$OS,

# GSE85916_dat$BUB1),

# title = 'BUB1 expression',

# labs = c('High','Low'),

# add_text = '')

# GSE85916_km

#GEO数据集合并

com_gene1=intersect(rownames(GSE21501_exp),rownames(GSE28735_exp))

com_gene2=intersect(intersect(rownames(GSE57495_exp),rownames(GSE62452_exp)),rownames(GSE85916_exp))

com_gene=intersect(com_gene1,com_gene2)

length(com_gene)

GSE_dat_before <- cbind(GSE21501_exp[com_gene, ],

GSE28735_exp[com_gene, ],

GSE57495_exp[com_gene,],

GSE62452_exp[com_gene, ],

GSE85916_exp[com_gene, ])

GSE_dat_before=na.omit(GSE_dat_before)

dim(GSE_dat_before)

GSE_dat_cli <-rbind(data.frame(OS.time=GSE21501_cli$OS.time/12,

OS=GSE21501_cli$OS,

Samples=GSE21501_cli$Samples,

GSE='GSE21501'),

data.frame(OS.time=GSE28735_cli$OS.time/12,

OS=GSE28735_cli$OS,

Samples=GSE28735_cli$Samples,

GSE='GSE28735'),

data.frame(OS.time=GSE57495_cli$OS.time/12,

OS=GSE57495_cli$OS,

Samples=GSE57495_cli$Samples,

GSE='GSE57495'),

data.frame(OS.time=GSE62452_cli$OS.time/12,

OS=GSE62452_cli$OS,

Samples=GSE62452_cli$Samples,

GSE='GSE62452'),

data.frame(OS.time=GSE85916_cli$OS.time/12,

OS=GSE85916_cli$OS,

Samples=GSE85916_cli$Samples,

GSE='GSE85916'))

rownames(GSE_dat_cli)=GSE_dat_cli$Samples

table(GSE_dat_cli$GSE)

GSE_dat_cli$OS.time <- GSE_dat_cli$OS.time * 365

# GSE_before 表达谱 PCA 分析 ############

library(ggbiplot)

GSE_before_pca <- as.data.frame(t(GSE_dat_before[, rownames(GSE_dat_cli)]),

stringsAsFactors = F)

#GSE_before_pca.pca<-prcomp(GSE_before_pca,scale.=T,center = F)

GSE_before_pca.pca=prcomp(scale(GSE_before_pca))

GSE_before_exp_pca <- ggbiplot(GSE_before_pca.pca, scale=1, groups = GSE_dat_cli$GSE,

ellipse = TRUE,ellipse.prob=0.5, circle = F,var.axes=F) +

ggsci::scale_color_lancet() + xlim(-3, 3) + ylim(-3, 3) + theme_bw() +

theme(legend.direction = 'horizontal', legend.position = 'top') +

xlab('PC1') + ylab('PC2')

GSE_before_exp_pca

# 去除批次效应 ###########

library(sva)

library(limma)

## 使用 limma 的 removeBatchEffect 函数

GSE_dat_exp <- removeBatchEffect(GSE_dat_before[, rownames(GSE_dat_cli)],

batch = GSE_dat_cli$GSE)

dim(GSE_dat_exp)

boxplot(GSE_dat_exp[, 1:50], las=2)

GSE_dat_exp <- normalizeBetweenArrays(GSE_dat_exp)

boxplot(GSE_dat_exp[, 1:50], las=2)

#GSE_after 表达谱 PCA 分析

library(ggbiplot)

GSE_after_pca <- as.data.frame(t(GSE_dat_exp[, rownames(GSE_dat_cli)]),

stringsAsFactors = F)

# GSE_after_pca.pca<-prcomp(GSE_after_pca, scale=T,center = F)

GSE_after_pca.pca<-prcomp(scale(GSE_after_pca))

GSE_dat_exp_pca <- ggbiplot(GSE_after_pca.pca, scale=1, groups = GSE_dat_cli$GSE,

ellipse = TRUE,ellipse.prob=0.5, circle = F,var.axes=F) +

ggsci::scale_color_lancet() + xlim(-3, 3) + ylim(-3, 3) + theme_bw() +

theme(legend.direction = 'horizontal', legend.position = 'top') +

xlab('PC1') + ylab('PC2')

GSE_dat_exp_pca

#数据消除批次效应前后的 PCA 图

GSE_PCA <- cowplot::plot_grid(GSE_before_exp_pca,

GSE_dat_exp_pca,

ncol = 2,nrow = 1,

labels = toupper(letters)[1:2],

align = "hv")

GSE_PCA

ggsave(plot = GSE_PCA,

filename = 'PDFs/GEO_PCA.pdf',

width = 12, height = 6, device = cairo_pdf)

save(GSE_dat_exp, file='origin_datas/GSE.batchremove.RData')

dim(GSE_dat_exp)

GEO_dat<-cbind(OS.time=GSE_dat_cli$OS.time,

OS=GSE_dat_cli$OS,

BUB1=as.numeric(t(GSE_dat_exp['BUB1',as.character(GSE_dat_cli$Samples)])))

GEO_dat=crbind2DataFrame(GEO_dat)

GEO_dat$BUB1=ifelse(GEO_dat$BUB1>mean(GEO_dat$BUB1),'High','Low')

# res.cut <- surv_cutpoint(GEO_dat,

# time = "OS.time",

# event = "OS",

# variables = c("BUB1"))

# GEO_dat$BUB1=ifelse(GEO_dat$BUB1>res.cut$cutpoint$cutpoint,'High','Low')

GEO_km<-ggplotKMCox(data.frame(GEO_dat$OS.time,

GEO_dat$OS,

GEO_dat$BUB1),

title = 'BUB1 expression',

labs = c('High','Low'),

add_text = '')

GEO_km

savePDF(filename = 'PDFs/GEO_km.pdf',plot = GEO_km,width = 7,height = 7)

save.image('PAAD_001.RData')

#研发#####

#devtools::install_github("navinlabcode/copykat")

library(copykat)

copykat.test <- copykat(rawmat=sce@assays$RNA@counts,

id.type="S",

cell.line="no",

ngene.chr=5,

#每个染色体中至少有 5 个基因来计算 DNA 拷贝数

win.size=25,

#每个片段至少取 25 个基因

KS.cut=0.15,

sam.name='paad',

distance="euclidean",

n.cores=4)

pred.test <- data.frame(copykat.test$prediction)

CNA.test <- data.frame(copykat.test$CNAmat)

table(pred.test$copykat.pred)

#绘制热图

my_palette <- colorRampPalette(rev(RColorBrewer::brewer.pal(n = 3, name = "RdBu")))(n = 999)

chr <- as.numeric(CNA.test$chrom) %% 2+1

rbPal1 <- colorRampPalette(c('black','grey'))

CHR <- rbPal1(2)[as.numeric(chr)]

chr1 <- cbind(CHR,CHR)

rbPal5 <- colorRampPalette(RColorBrewer::brewer.pal(n = 8, name = "Dark2")[2:1])

com.preN <- pred.test$copykat.pred

pred <- rbPal5(2)[as.numeric(factor(com.preN))]

cells <- rbind(pred,pred)

col_breaks = c(seq(-1,-0.4,length=50),seq(-0.4,-0.2,length=150),seq(-0.2,0.2,length=600),seq(0.2,0.4,length=150),seq(0.4, 1,length=50))

heatmap.3(t(CNA.test[,4:ncol(CNA.test)]),dendrogram="r", distfun = function(x) parallelDist::parDist(x,threads =4, method = "euclidean"), hclustfun = function(x) hclust(x, method="ward.D2"),

ColSideColors=chr1,RowSideColors=cells,Colv=NA, Rowv=TRUE,

notecol="black",col=my_palette,breaks=col_breaks, key=TRUE,

keysize=1, density.info="none", trace="none",

cexRow=0.1,cexCol=0.1,cex.main=1,cex.lab=0.1,

symm=F,symkey=F,symbreaks=T,cex=1, cex.main=4, margins=c(10,10))

legend("topright", paste("pred.",names(table(com.preN)),sep=""), pch=15,col=RColorBrewer::brewer.pal(n = 8, name = "Dark2")[2:1], cex=0.6, bty="n")

#定义非整倍体肿瘤细胞的亚群

tumor.cells <- pred.test$cell.names[which(pred.test$copykat.pred=="aneuploid")]

tumor.mat <- CNA.test[, which(colnames(CNA.test) %in% tumor.cells)]

hcc <- hclust(parallelDist::parDist(t(tumor.mat),threads =4, method = "euclidean"), method = "ward.D2")

hc.umap <- cutree(hcc,2)

rbPal6 <- colorRampPalette(RColorBrewer::brewer.pal(n = 8, name = "Dark2")[3:4])

subpop <- rbPal6(2)[as.numeric(factor(hc.umap))]

cells <- rbind(subpop,subpop)

heatmap.3(t(tumor.mat),dendrogram="r", distfun = function(x) parallelDist::parDist(x,threads =4, method = "euclidean"), hclustfun = function(x) hclust(x, method="ward.D2"),

ColSideColors=chr1,RowSideColors=cells,Colv=NA, Rowv=TRUE,

notecol="black",col=my_palette,breaks=col_breaks, key=TRUE,

keysize=1, density.info="none", trace="none",

cexRow=0.1,cexCol=0.1,cex.main=1,cex.lab=0.1,

symm=F,symkey=F,symbreaks=T,cex=1, cex.main=4, margins=c(10,10))

legend("topright", c("c1","c2"), pch=15,col=RColorBrewer::brewer.pal(n = 8, name = "Dark2")[3:4], cex=0.9, bty='n')

############售后##################

#补

load('sce2.RData')

FeaturePlot(sce,features = "BUB1",reduction = "umap",pt.size = 1)+

scale_x_continuous("")+scale_y_continuous("")+

theme_bw()

pdf('BUB1.vlnplot.pdf',he=5,wi=9)

VlnPlot(object = sce,

features = 'BUB1',

pt.size = 0)

dev.off()

pdf('doplot_C14.pdf',he=15,wi=15)

doplot_C14

dev.off()

pdf('doplot_C16.pdf',he=15,wi=15)

doplot_C16

dev.off()

sce_scale_dat<-as.matrix(sce@assays$RNA@scale.data)

sce_scale_dat[1:4,1:4]

write.table(sce_scale_dat,file = 'sce_scale_dat.txt',quote = F,row.names = T,sep='\t')

sce_meta<-as.matrix(sce@meta.data)

sce_meta=sce_meta[,c("Sample","Group1","Group2","seurat_clusters","nCount_RNA","nFeature_RNA")]

write.table(sce_meta,'售后/sce_meta.txt',quote = F,sep='\t',row.names = T)

#售后-20220424####################

source('/pub1/data/mg_projects/projects/codes/mg_base.R')

#BUB1基因在癌和癌旁的表达

library(ggpubr)

bub1.gene<-read.delim('results/shouhou_20220424/BUB1.exp.txt',sep='\t',header = T)

bub1.gene=bub1.gene[bub1.gene$X=='PAAD(T=178,N=171)',]

sig_boxplot<-function(dat,leg,ylab,palette=ggsci::pal_lancet()(10)){

dat=na.omit(dat)

colnames(dat)=c('group','gene')

dat=dat[order(dat$group),]

all.combn=combn(as.character(unique(dat$group)),2)

my_comparisons=lapply(seq_len(ncol(all.combn)), function(i) all.combn[,i])

pp=ggboxplot(dat,

x='group', y='gene', color = 'group',

palette = palette,

short.panel.labs = T,outlier.shape = NA)+

stat_compare_means(comparisons=my_comparisons,method="wilcox.test",label = "p.signif")+

ylab(ylab)+xlab('')+labs(color=leg)

return(pp)

}

fig1<-sig_boxplot(dat=bub1.gene[,c("Group","Expression")],

leg='Type',

ylab='log2(Gene Expression+0.001)',

palette=ggsci::pal_lancet()(9)[2:3])

fig1

ggsave('results/shouhou_20220424/Fig1.pdf',fig1,height = 5,width = 5)

#2、与免疫的关系

tcga.dat<-read.delim('tcga_exp_t.txt',sep='\t',header = T,row.names = 1)

colnames(tcga.dat)=gsub('\\.','-',colnames(tcga.dat))

tcga.dat[1:4,1:4]

bub1.gene<-data.frame(Samples=colnames(tcga.dat),

BUB1.exp=t(tcga.dat['BUB1',]))

head(bub1.gene)

bub1.gene$group=ifelse(bub1.gene$BUB1>median(bub1.gene$BUB1),'High','Low')

#tcga.cib<-immu_CIBERSORT(exp_data = tcga.dat)

#save(tcga.cib,file = 'tcga.cib.RData')

load('tcga.cib.RData')

fig2a<-mg_PlotMutiBoxplot(tcga.cib[bub1.gene$Samples,1:22]

, group = bub1.gene$group

, legend.pos = 'top'

, add = 'boxplot'

, ylab = 'Score'

, group_cols = ggsci::pal_lancet()(10)

, test_method = 'wilcox.test')

fig2a

ggsave('results/shouhou_20220424/fig2a.pdf',fig2a,height = 7,width = 12)

tcga_mcp<-immu_MCPcounter(exp = tcga.dat,isTCGA = T)

fig2b<-mg_PlotMutiBoxplot(tcga_mcp[bub1.gene$Samples,]

, group = bub1.gene$group

, legend.pos = 'top'

, add = 'boxplot'

, ylab = 'Score'

, group_cols = ggsci::pal_lancet()(10)

, test_method = 'wilcox.test')

fig2b

ggsave('results/shouhou_20220424/fig2b.pdf',fig2b,height = 7,width = 9)

# tcga.esti<-immu_estimate(exp = tcga.dat,platform='illumina',isTCGA=T)

# tcga.esti=crbind2DataFrame(tcga.esti)

fig3<-mg_PlotMutiBoxplot(tcga.esti[bub1.gene$Samples,1:3]

, group = bub1.gene$group

, legend.pos = 'top'

, add = 'boxplot'

, ylab = 'Score'

, group_cols = ggsci::pal_lancet()(10)

, test_method = 'wilcox.test')

fig3

ggsave('results/shouhou_20220424/fig3.pdf',fig3,height = 5,width = 7)

#4、GSVA分析

library(GSVA)

library(GSEABase)

c2KEGG <- getGmt("/pub1/data/mg_projects/projects/codes/source/c2.cp.kegg.v7.0.symbols.gmt",

collectionType=BroadCollection(category="c2"),

geneIdType=SymbolIdentifier())

ssGSEA <- gsva(as.matrix(tcga.dat),

c2KEGG,

method='ssgsea',

min.sz=10,

max.sz=500,

verbose=TRUE)

#相关性和显著性分析

ssGSEA[1:4,1:4]

tcga.kegg<-cbind.data.frame(BUB1=bub1.gene$BUB1,

t(ssGSEA[,bub1.gene$Samples]))

tcga.kegg[1:4,1:4]

tcga.kegg.cor<-Hmisc::rcorr(as.matrix(tcga.kegg),type = 'pearson')

tcga.kegg.cor.r=reshape2::melt(tcga.kegg.cor$r)

tcga.kegg.cor.p=reshape2::melt(tcga.kegg.cor$P)

colnames(tcga.kegg.cor.r)=c('pathway','gene','cor')

colnames(tcga.kegg.cor.p)=c('pathway','gene','p')

tcga.kegg.cor.r=tcga.kegg.cor.r[which(tcga.kegg.cor.r$pathway != 'BUB1' & tcga.kegg.cor.r$gene== 'BUB1'),]

tcga.kegg.cor.p=tcga.kegg.cor.p[which(tcga.kegg.cor.p$pathway != 'BUB1' & tcga.kegg.cor.p$gene== 'BUB1'),]

tcga.kegg.cor.res<-merge(tcga.kegg.cor.p,tcga.kegg.cor.r,by=c('pathway','gene'))

head(tcga.kegg.cor.res)

tcga.kegg.cor.res.fit<-tcga.kegg.cor.res[abs(tcga.kegg.cor.res$cor)>0.4 & tcga.kegg.cor.res$p<0.05,]

dim(tcga.kegg.cor.res.fit)

write.table(tcga.kegg.cor.res,'results/shouhou_20220424/tcga.kegg.cor.res.txt',quote = F,row.names = F,sep='\t')

#相关性热图

tcga.kegg.cor$P[is.na(tcga.kegg.cor$P)] <- 0

library(corrplot)

col1 = colorRampPalette(c('blue', 'white','red'))

tcga.kegg1=tcga.kegg[,c(as.character(tcga.kegg.cor.res.fit$pathway),'BUB1')]

colnames(tcga.kegg1)=gsub('KEGG_','',colnames(tcga.kegg1))

tcga.kegg.cor1<-Hmisc::rcorr(as.matrix(tcga.kegg1),type = 'pearson')

tcga.kegg.cor1$P[is.na(tcga.kegg.cor1$P)] <- 0

pdf('results/shouhou_20220424/fig4.pdf',height = 12,width = 12)

corrplot(as.matrix(crbind2DataFrame(tcga.kegg.cor1$r)),

p.mat = as.matrix(crbind2DataFrame(tcga.kegg.cor1$P)),

mar = c(0,0,1,1),

col=col1(100),

tl.srt = 90,

tl.cex = 0.5,

tl.col = 'black',

tl.offset = 0.5,

cl.pos = c("b","r","n")[1],

cl.align.text = 'l',

cl.length = 5,

cl.ratio = 0.1,

cl.cex = 0.8,

addgrid.col = 'white',

method = 'color',

insig = 'label_sig',

sig.level=c(0.001,0.01,0.05),

pch.cex=1,

is.corr=T,

xpd=T

)

dev.off()

#

tcga_cli<-read.delim('origin_datas/TCGA/Merge_clinical.txt',sep='\t',header = T,check.names = F)

tcga_cli<-tcga_cli[which(tcga_cli$A0_Barcode=='PAAD'),]

tcga_cli <- tcga_cli[, c("A0_Samples", "A1_OS", "A2_Event", "A3_T", "A4_N", "A5_M",

"A6_Stage", "A18_Sex",

"age_at_initial_pathologic_diagnosis")]

colnames(tcga_cli) <- c("Samples", "OS.time", "OS", "T.Stage", "N.Stage", "M.Stage",

"Stage", "Gender", "Age")

head(tcga_cli)

tcga_cli=crbind2DataFrame(tcga_cli)

table(tcga_cli$OS)

table(tcga_cli$T.Stage)

table(tcga_cli$N.Stage)

table(tcga_cli$M.Stage)

table(tcga_cli$Stage)

table(tcga_cli$Gender)

median(tcga_cli$Age)

table(tcga_cli$Age>65)

paad_data=mg_data_hub_PAAD(dtype = 'mEXP')

names(paad_data)

GSE21501_cli1=paad_data$GSE21501$CLINI

table(GSE21501_cli1$Clinical_T)

table(GSE21501_cli1$Clinical_Stage)

GSE21501_cli2=paad_data$GSE21501$OS

table(GSE21501_cli2$OS_EVENT)

GSE28735_cli1=paad_data$GSE28735$OS

table(GSE28735_cli1$OS_EVENT)

GSE28735_cli2=paad_data$GSE28735$CLINI

table(GSE28735_cli2$TISSUE)

GSE57495_cli1<-paad_data$GSE57495$OS

table(GSE57495_cli1$OS_EVENT)

GSE57495_cli2<-paad_data$GSE57495$CLINI

table(GSE57495_cli2$Clinical_Stage)

GSE62452_cli1<-paad_data$GSE62452$OS

table(GSE62452_cli1$OS_EVENT)

GSE62452_cli2<-paad_data$GSE62452$CLINI

table(GSE62452_cli2$Clinical_Stage)

table(GSE62452_cli2$Clinical_Grade)

table(GSE62452_cli2$TISSUE)

GSE85916_cli1<-paad_data$GSE85916$OS

table(GSE85916_cli1$OS_EVENT)

GSE85916_cli2<-paad_data$GSE85916$CLINI

table(GSE85916_cli2)
