# Supplementary figures and images for: BUB1 Is Identified as a Potential Therapeutic Target for Pancreatic Cancer Treatment
Source: Front Public Health. 2022 Jun 13;10:900853. doi: 10.3389/fpubh.2022.900853 (PMC9235519; doi:10.3389/fpubh.2022.900853)

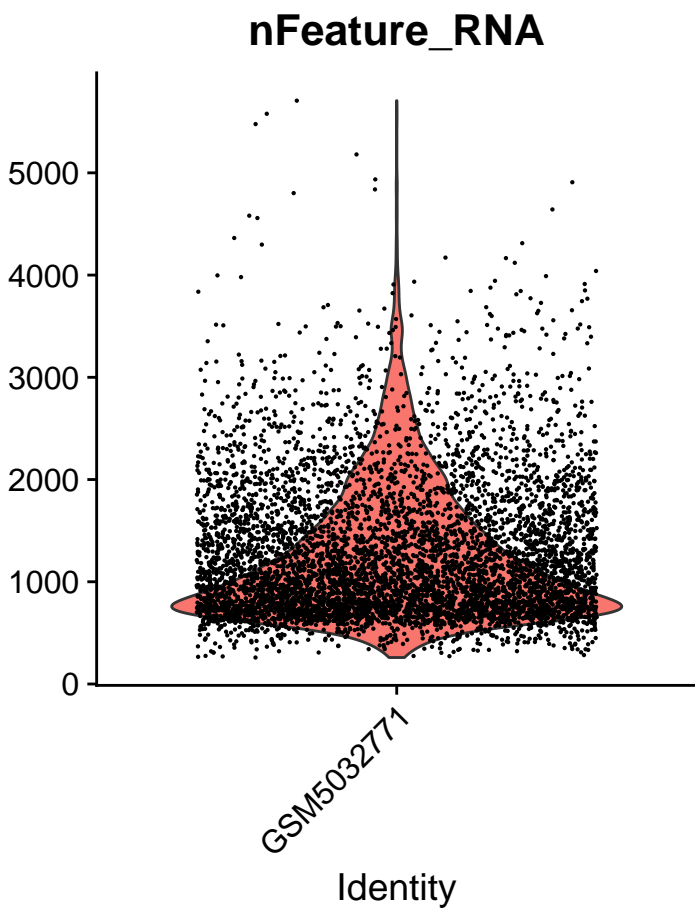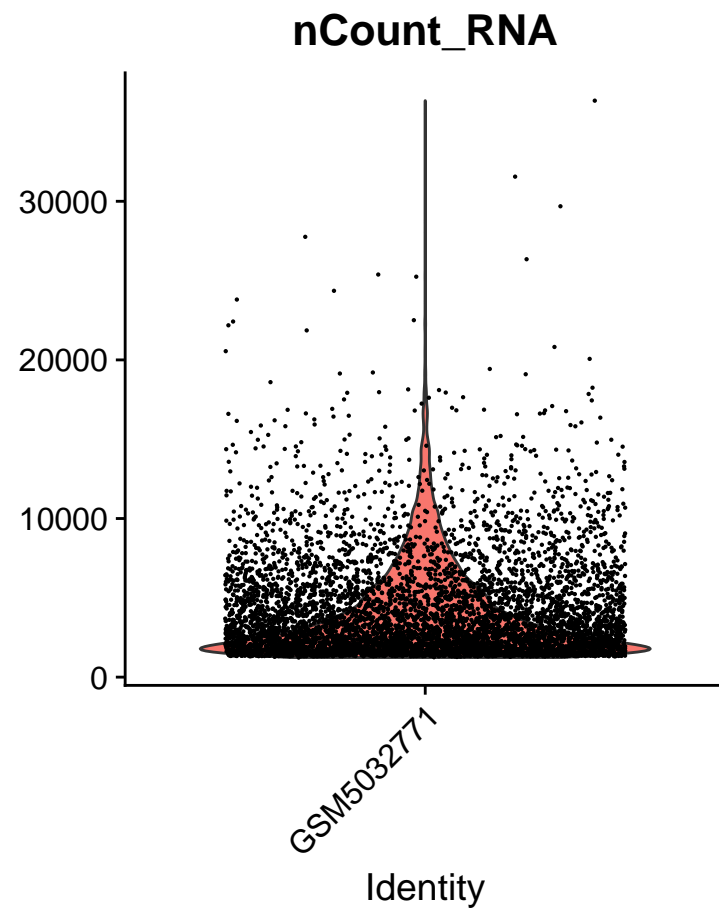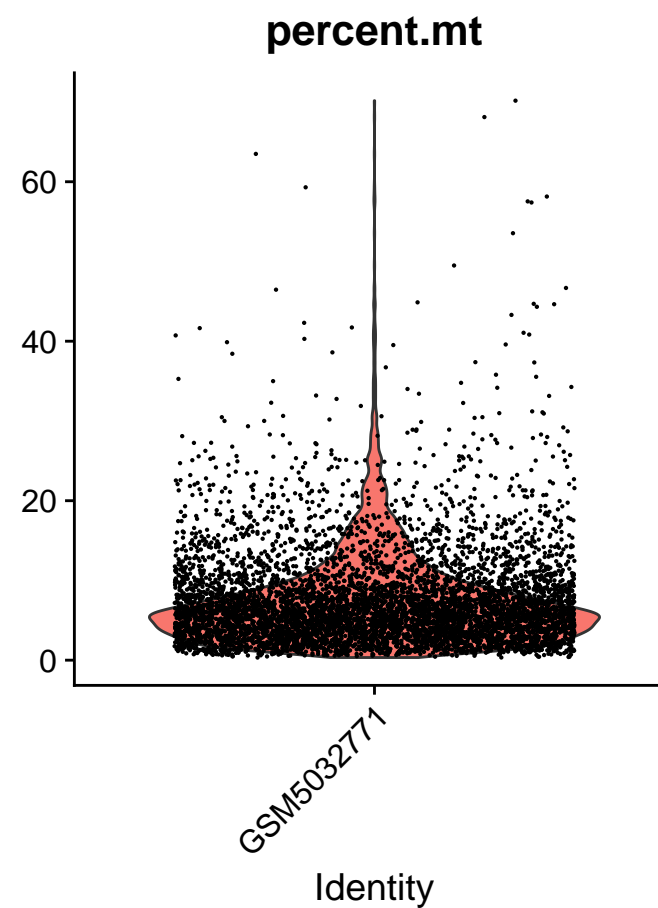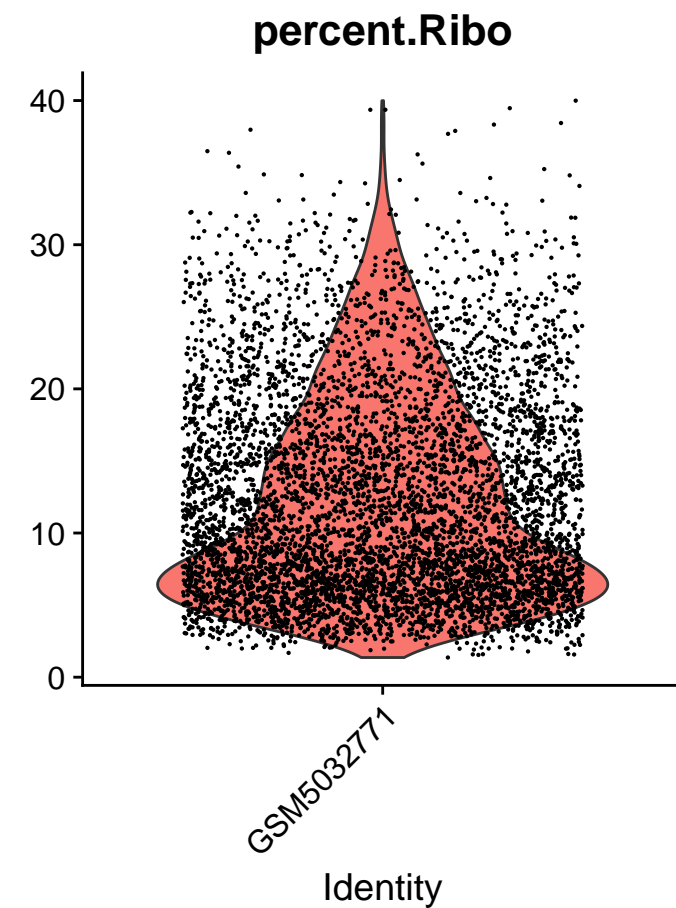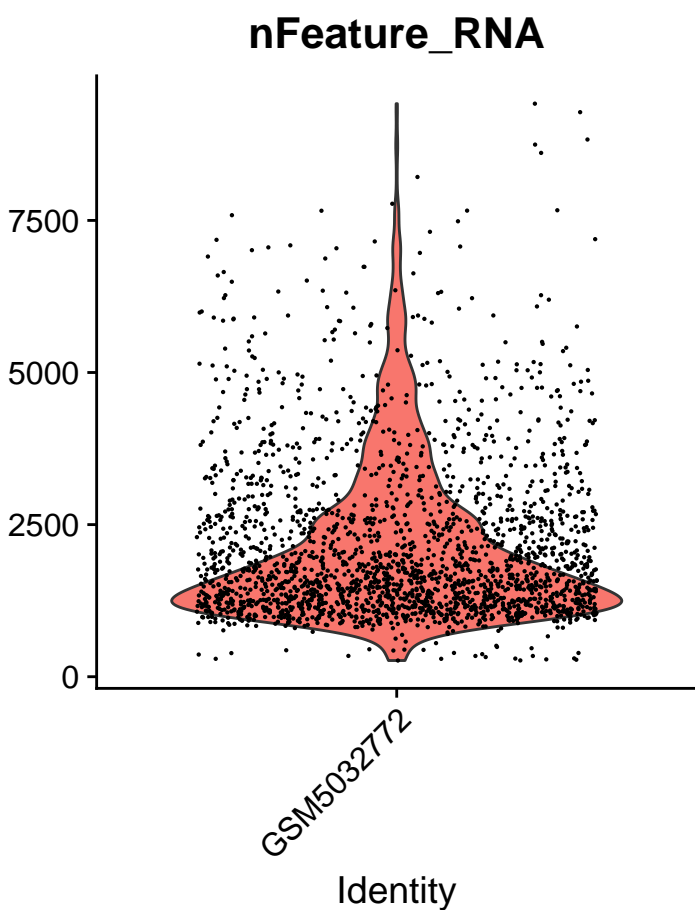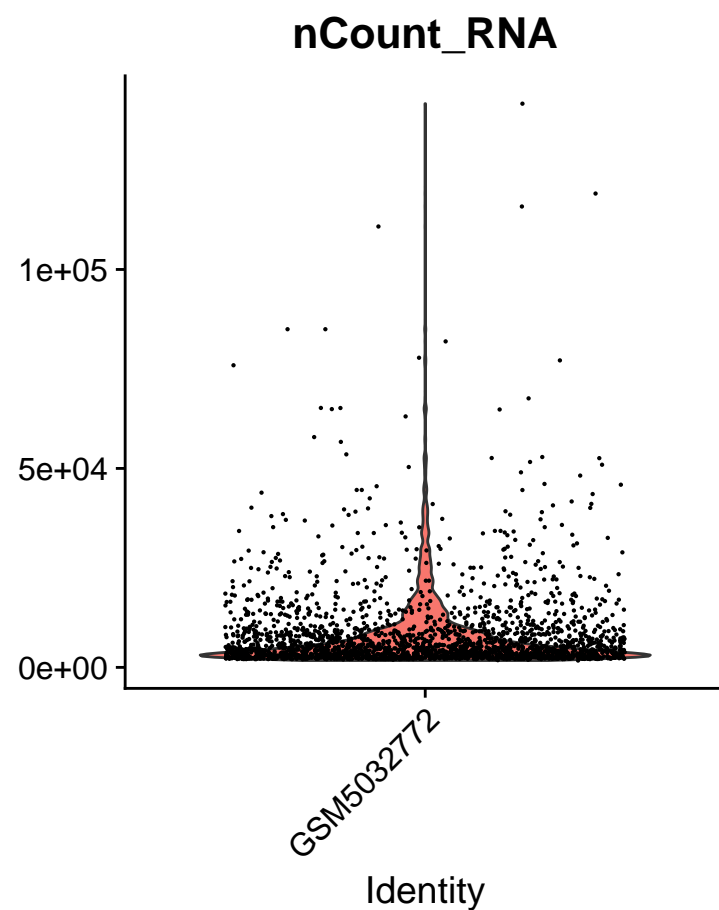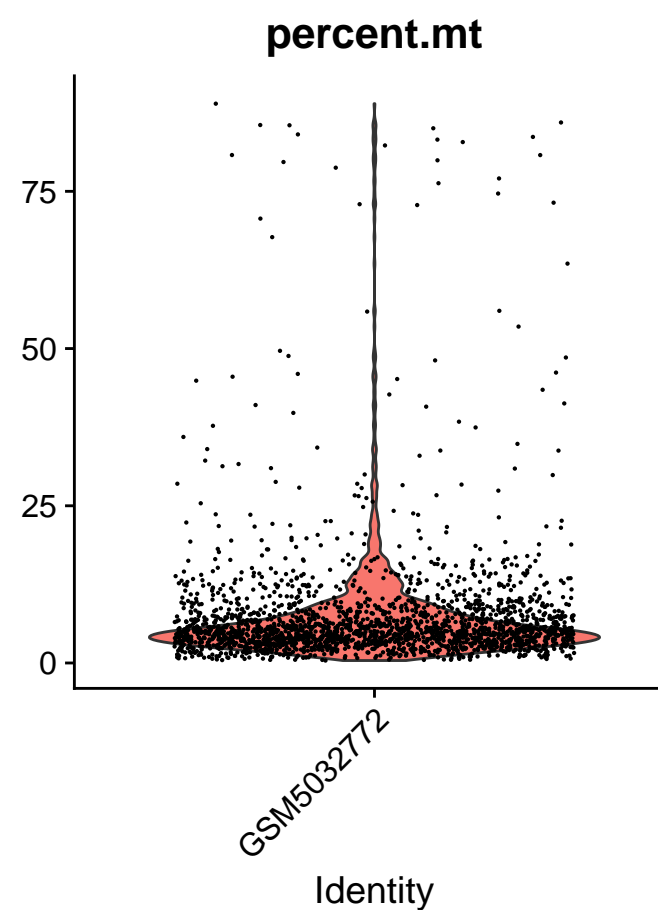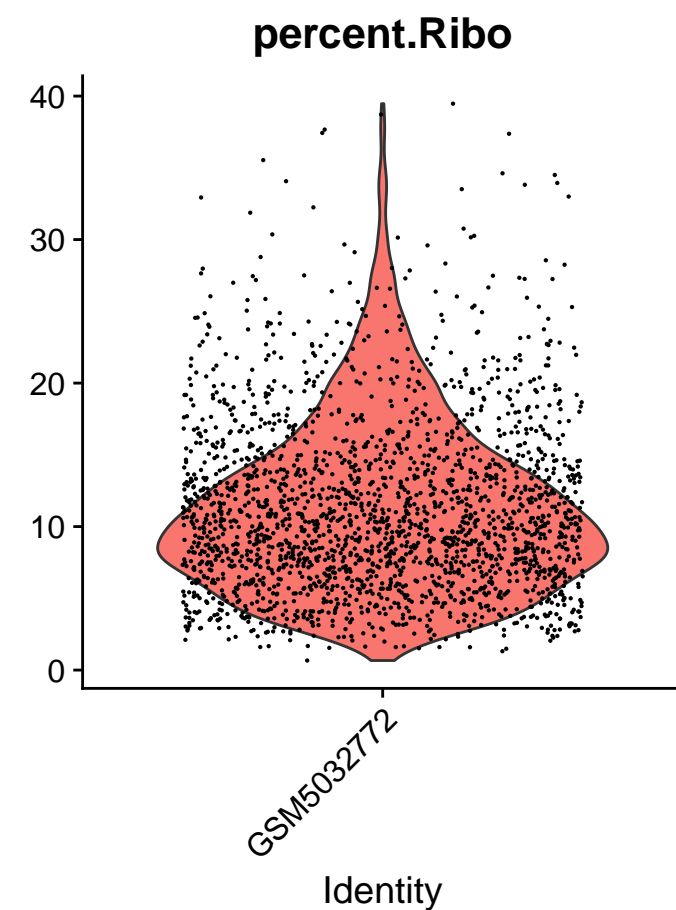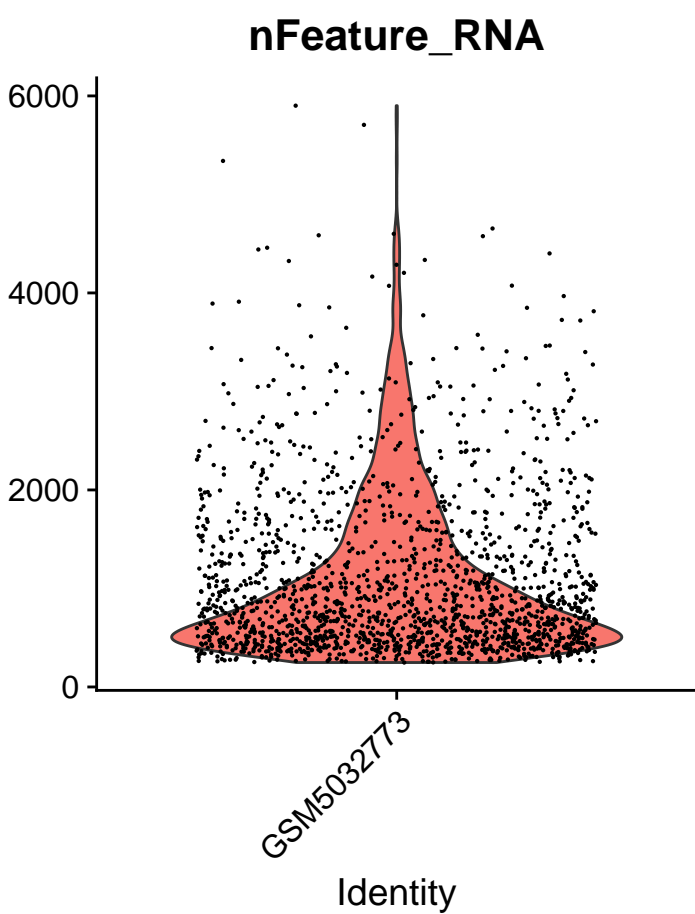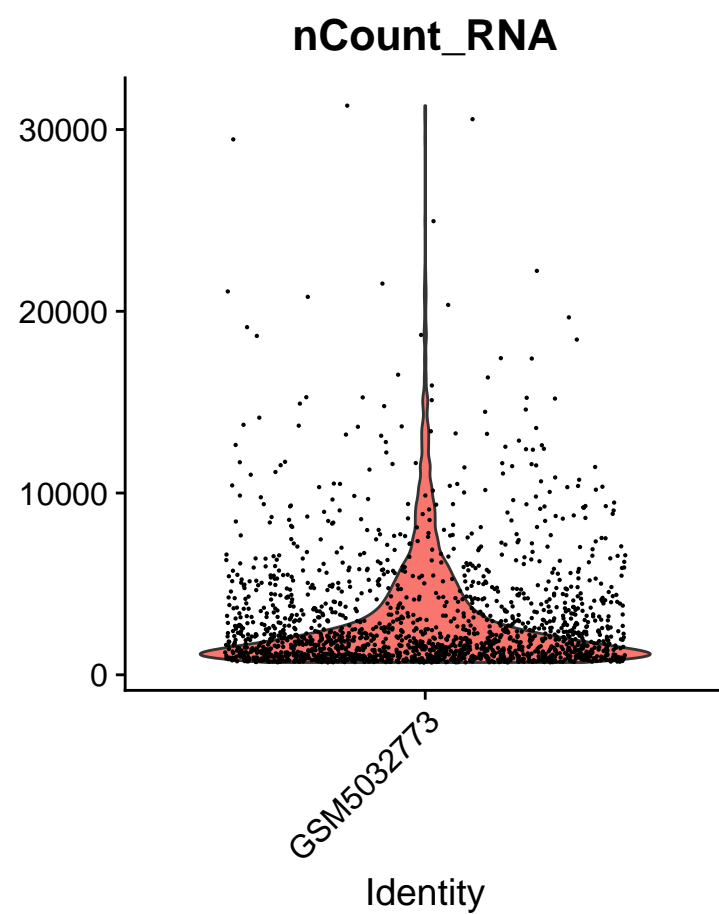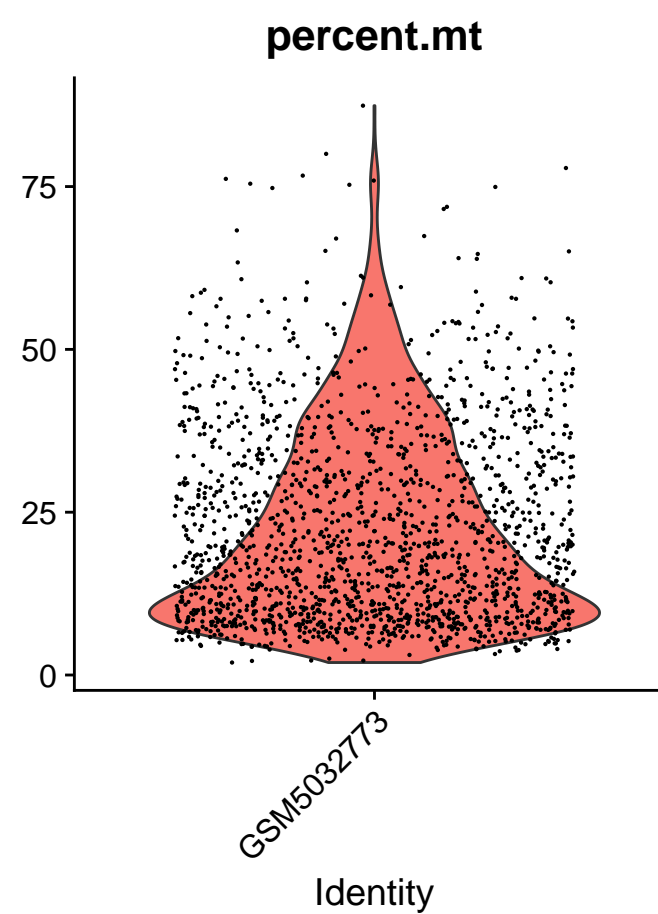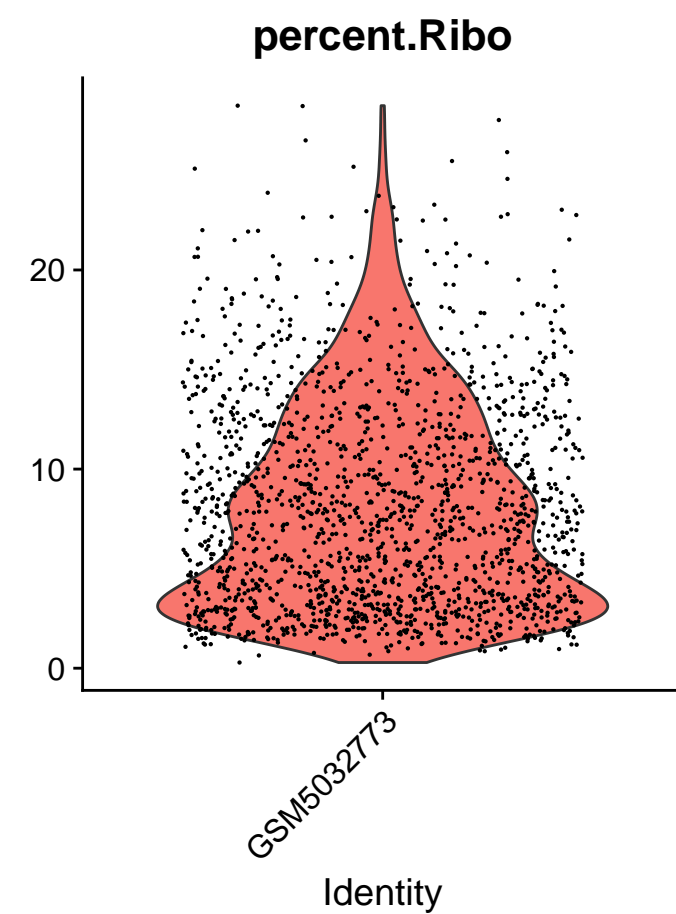

Supplement: Supplementary file 3 [file Data_Sheet_1.PDF]

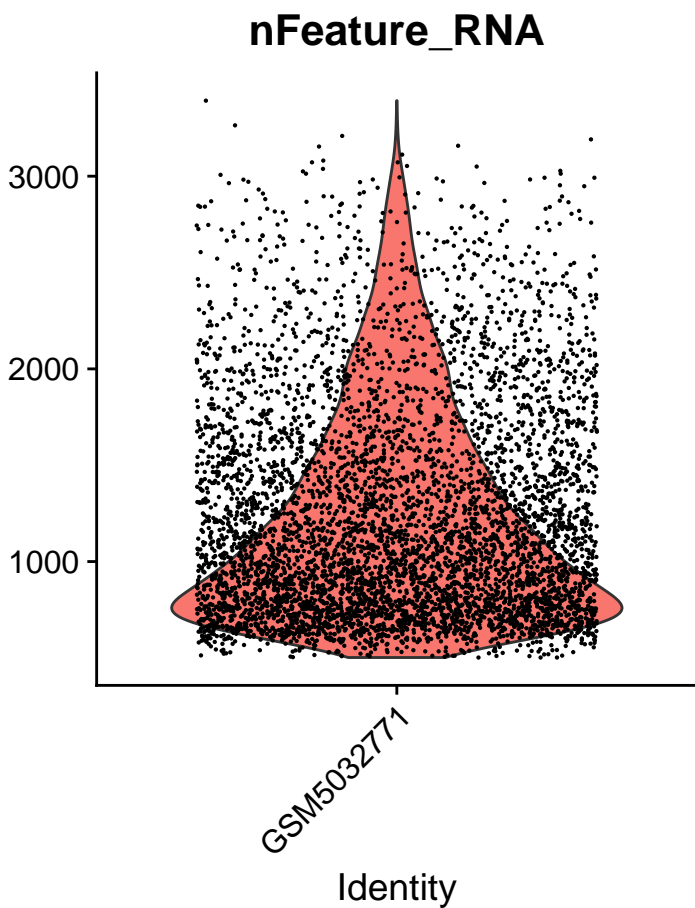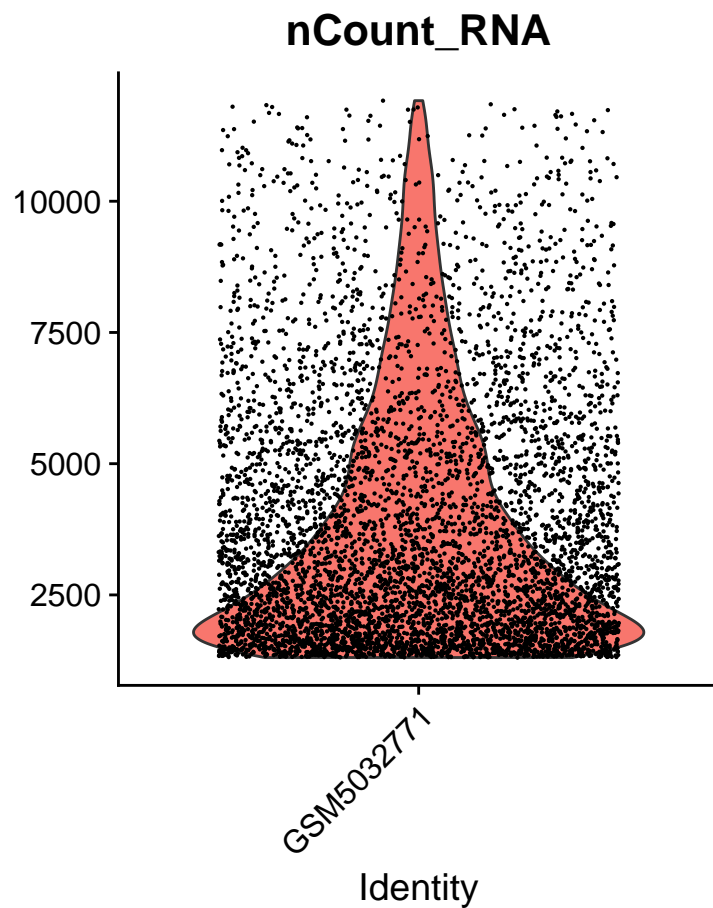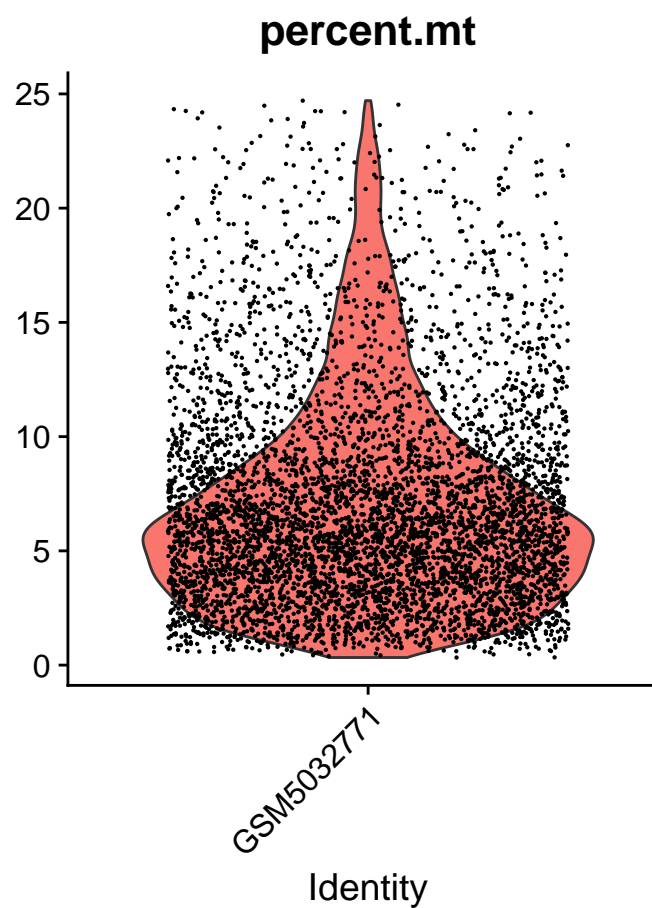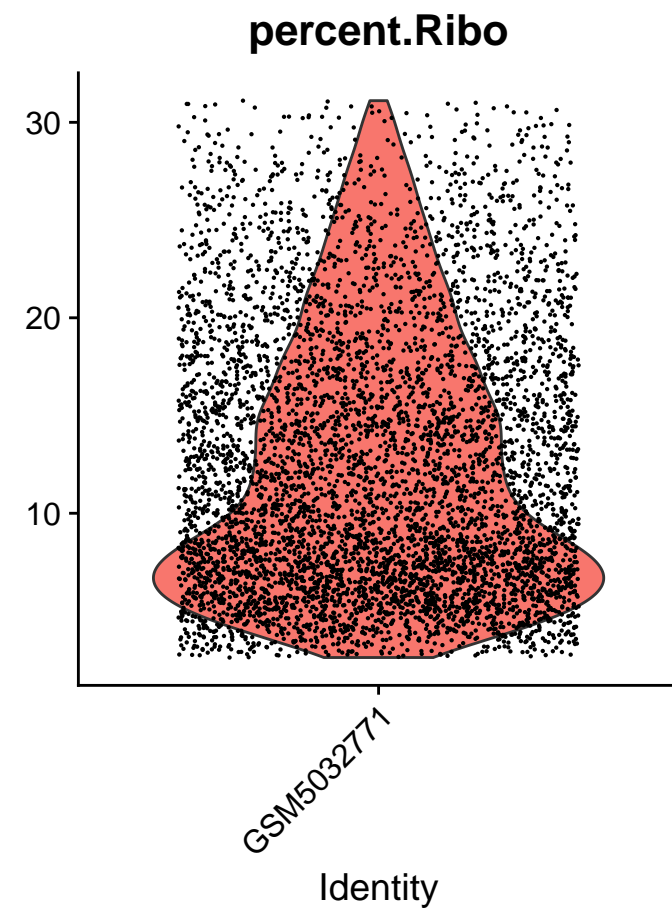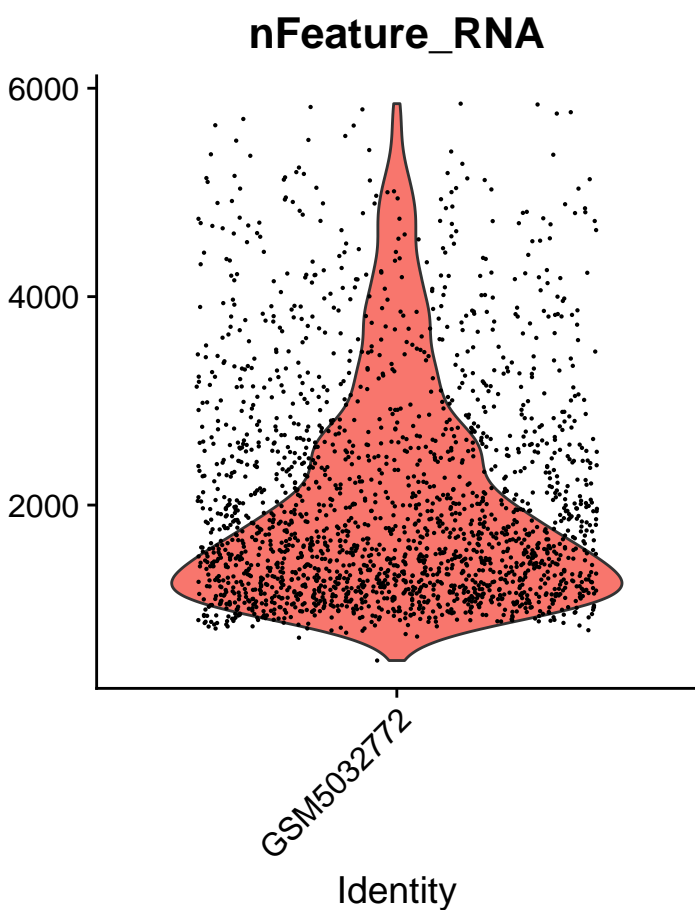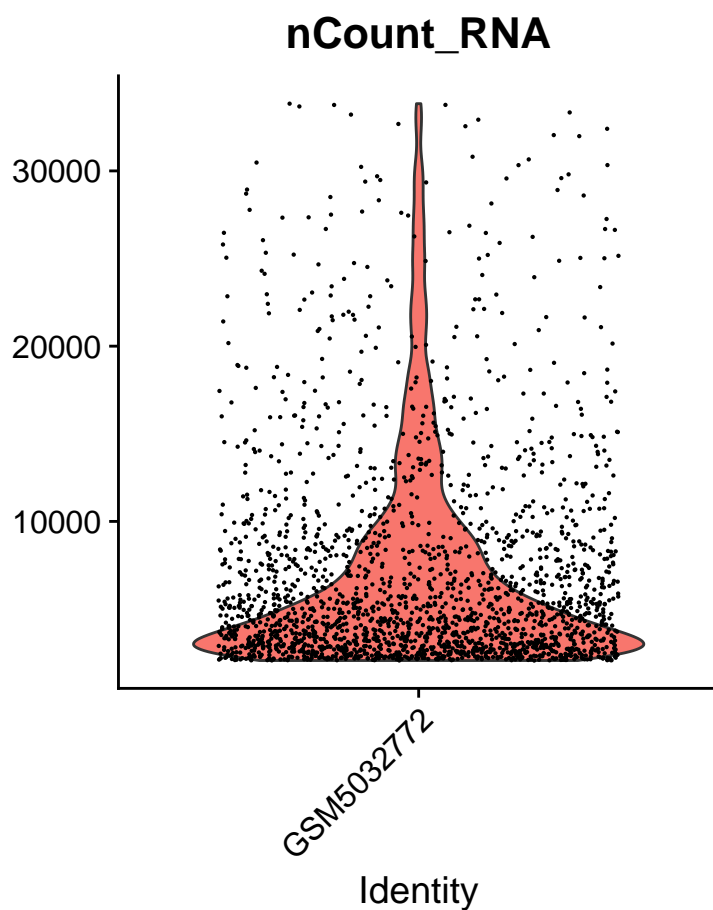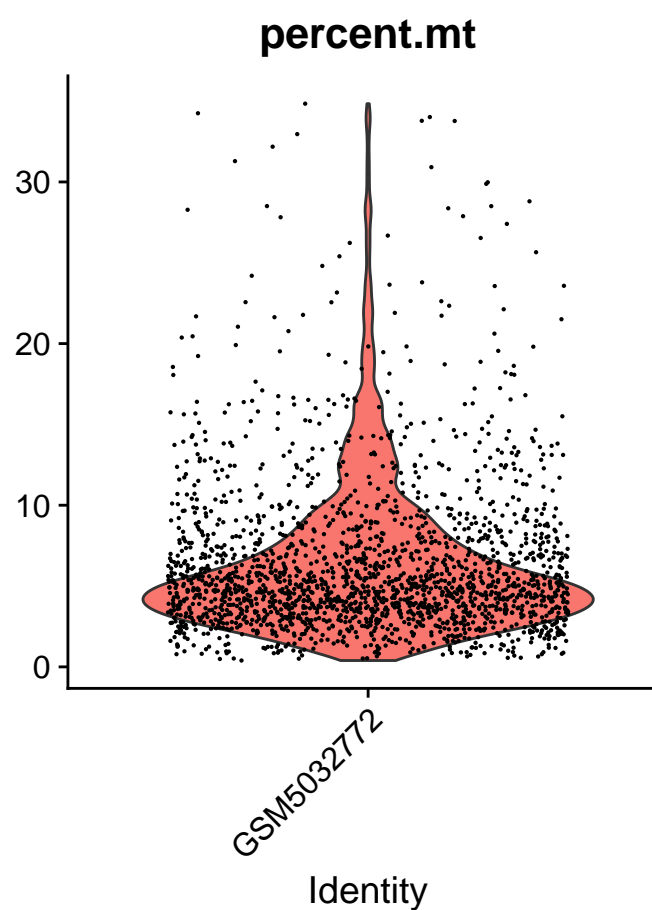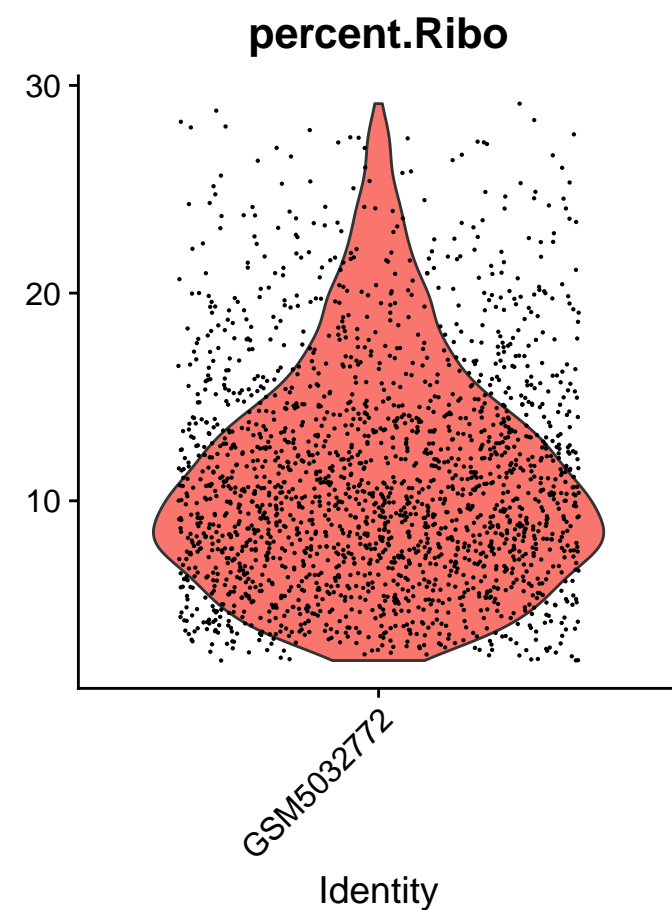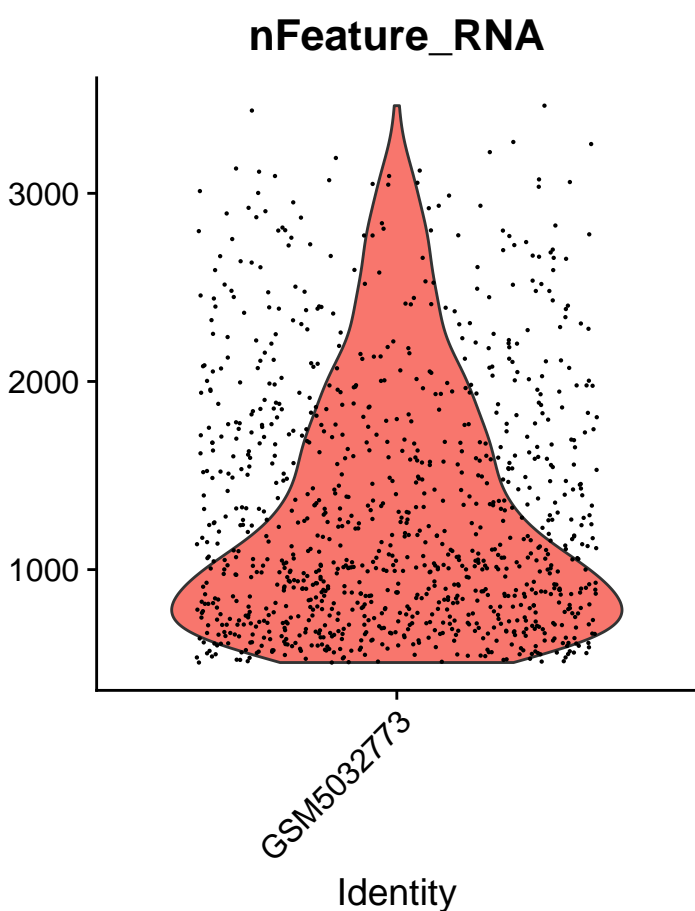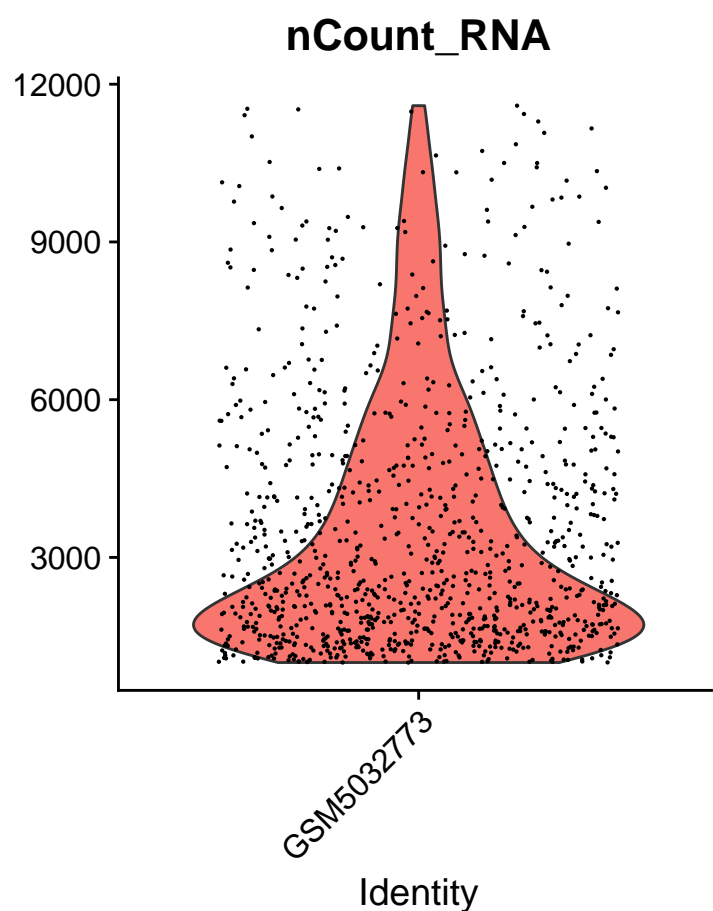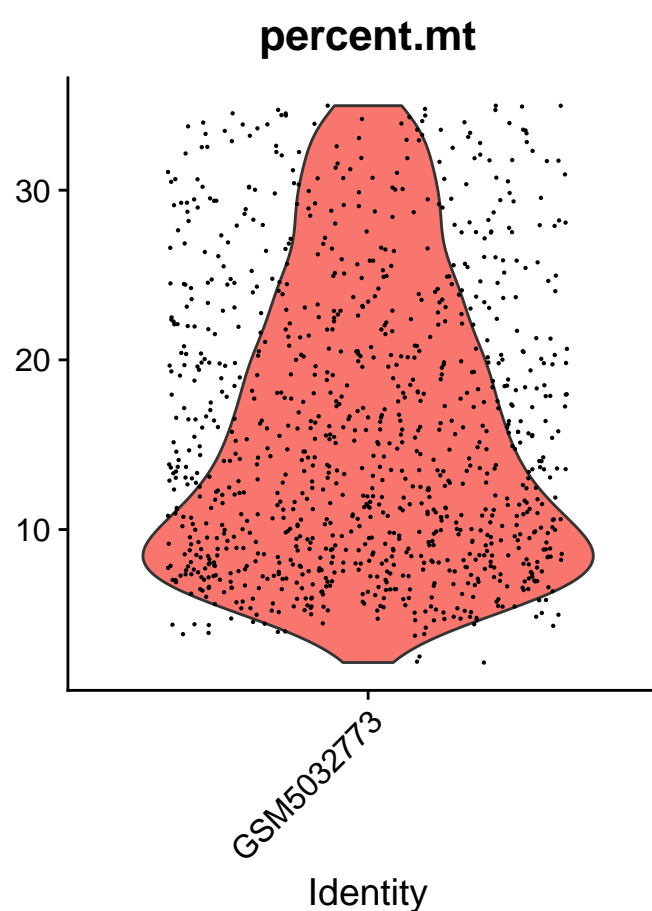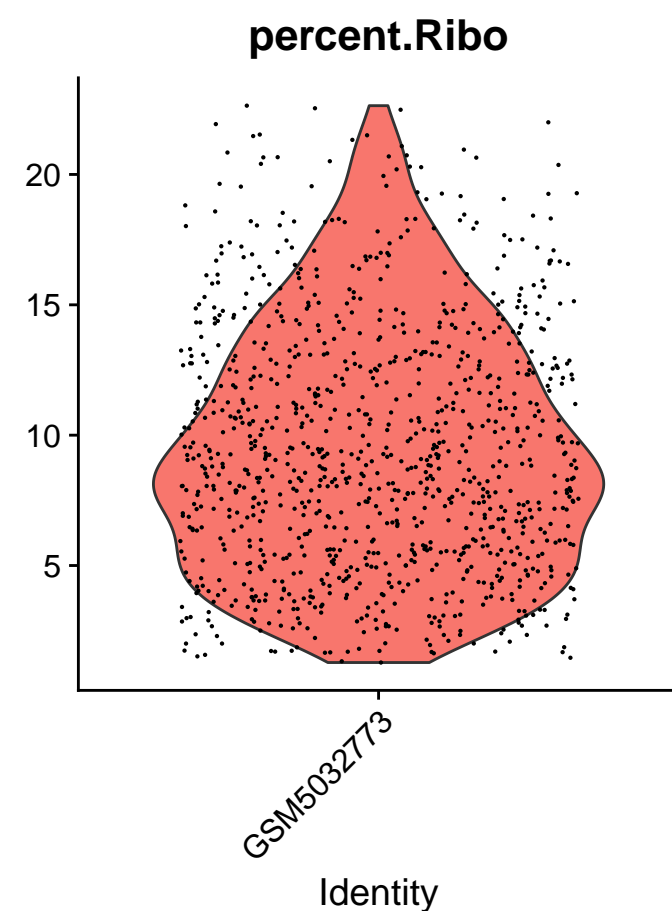

Supplement: Supplementary file 4 [file Data_Sheet_2.PDF]

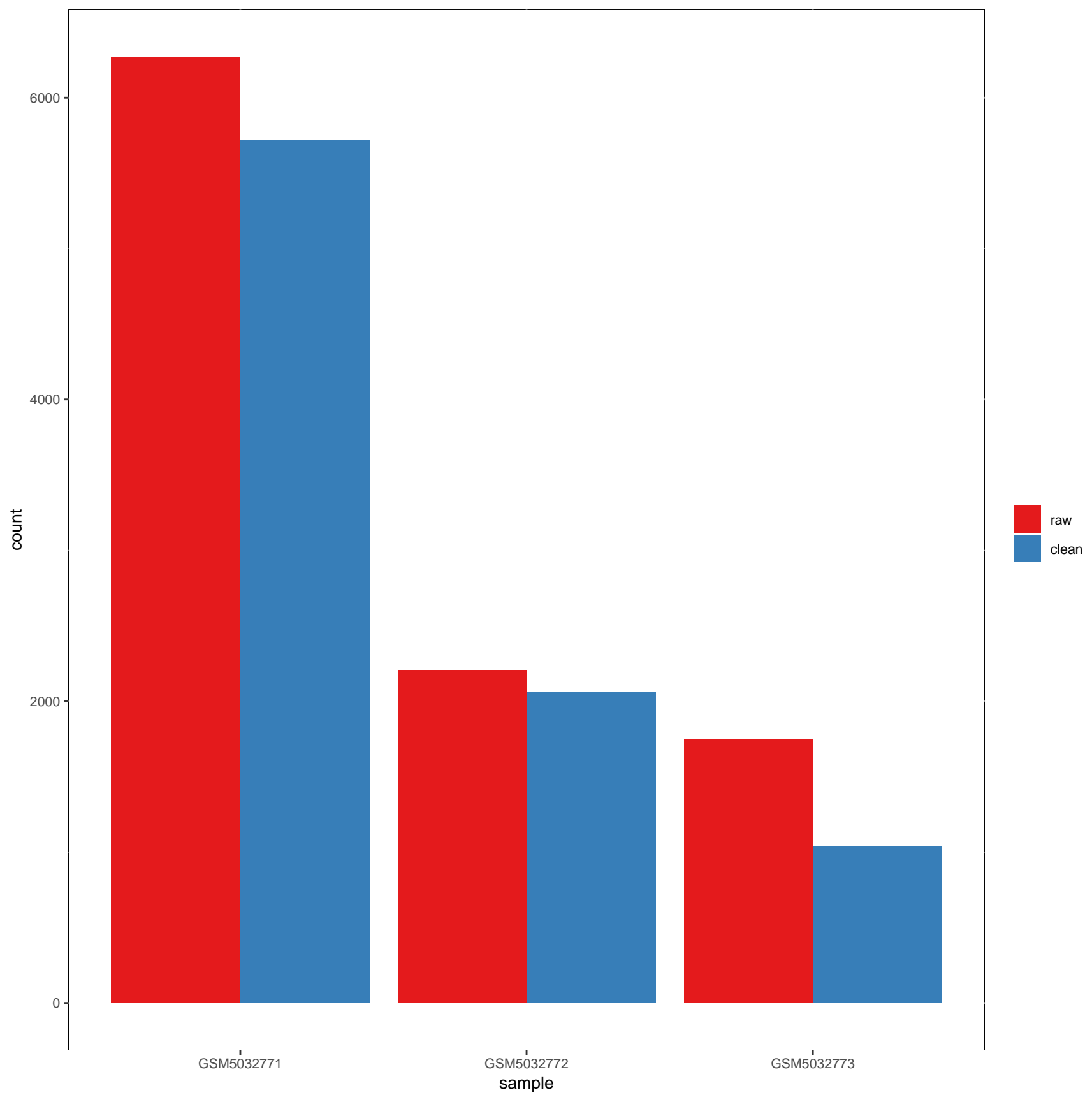

Supplement: Supplementary file 5 [file Data_Sheet_3.PDF]

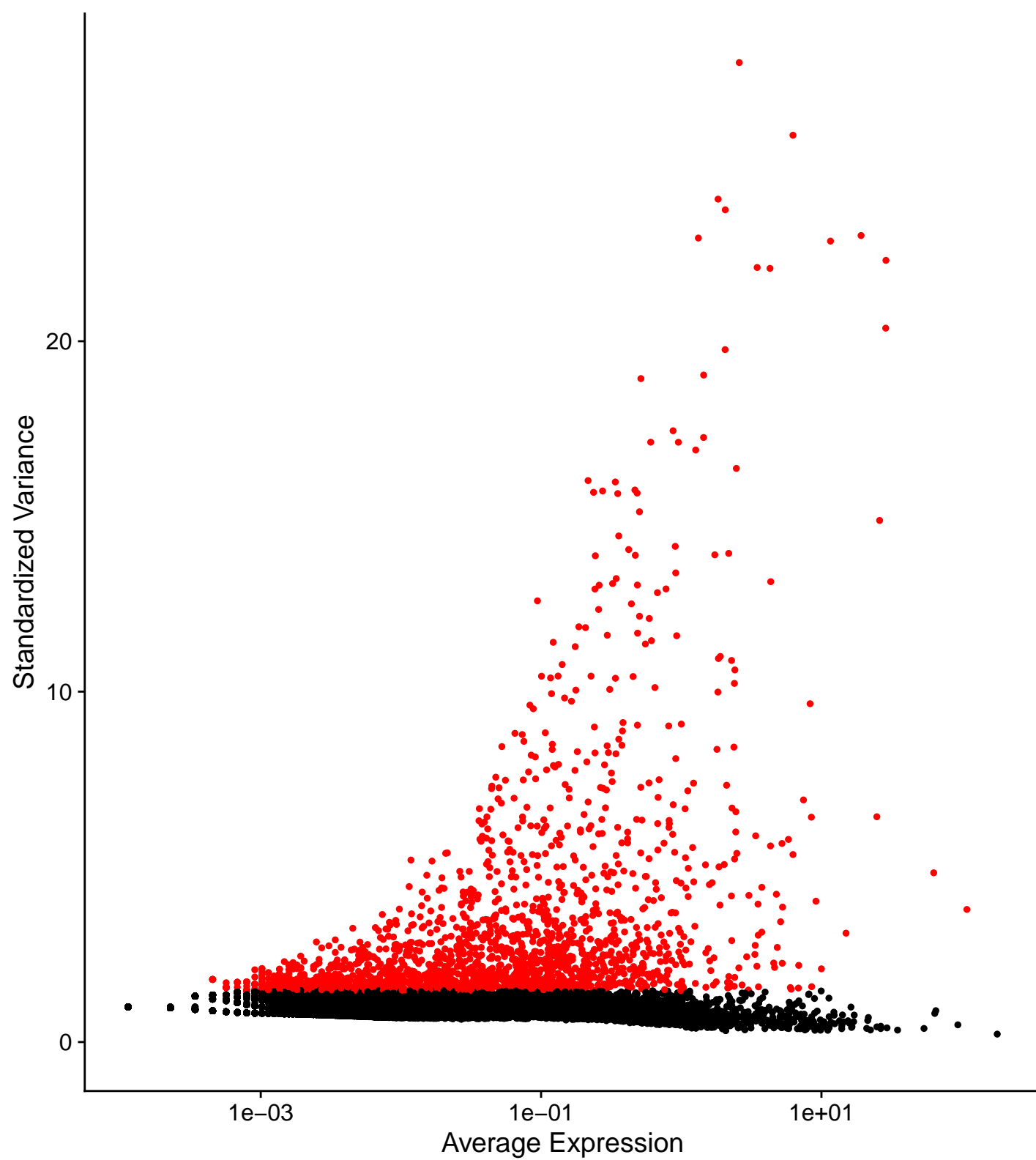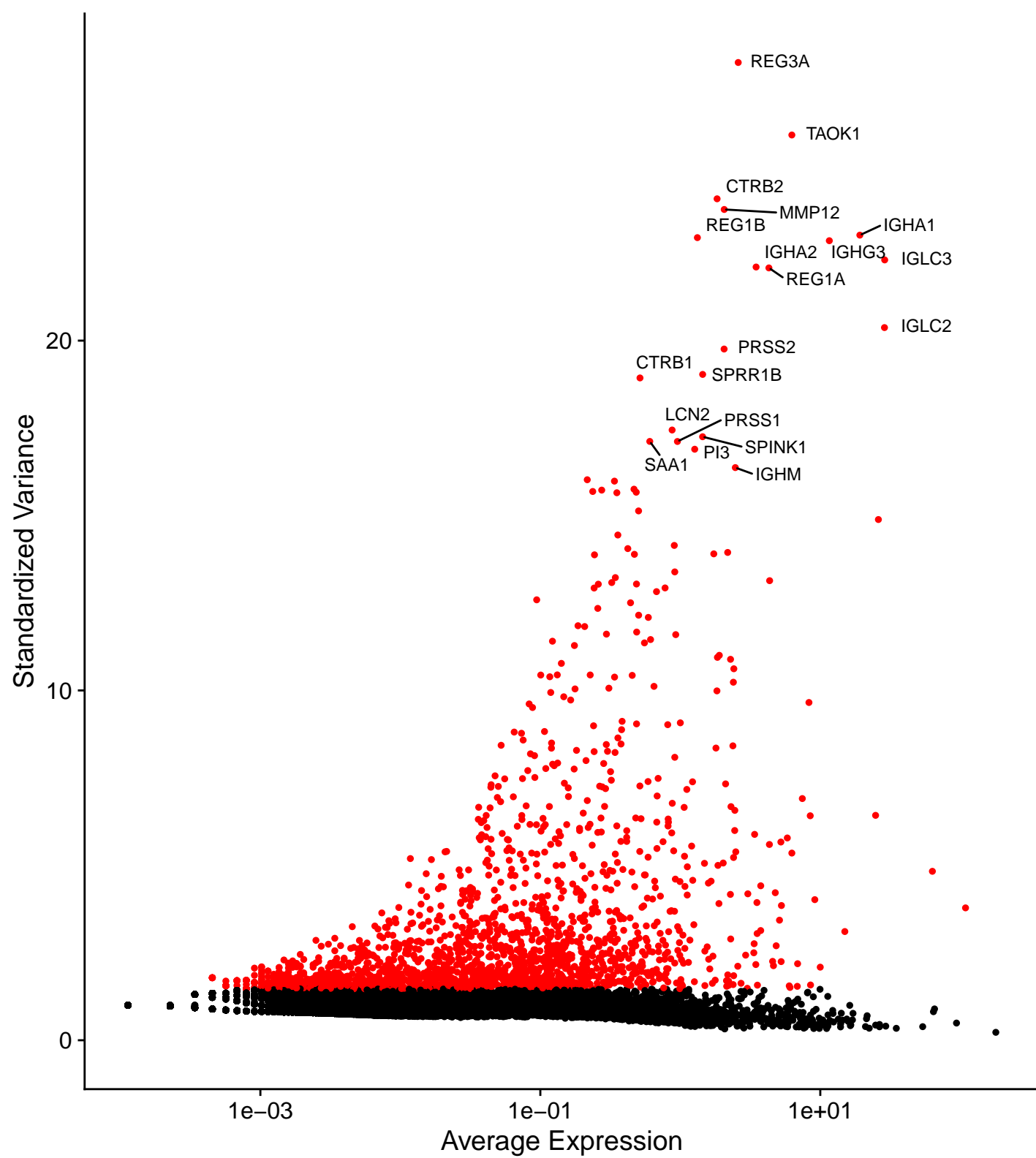

• Non-variable count: 23353 • Variable count: 2000

Supplement: Supplementary file 6 [file Data_Sheet_4.PDF]

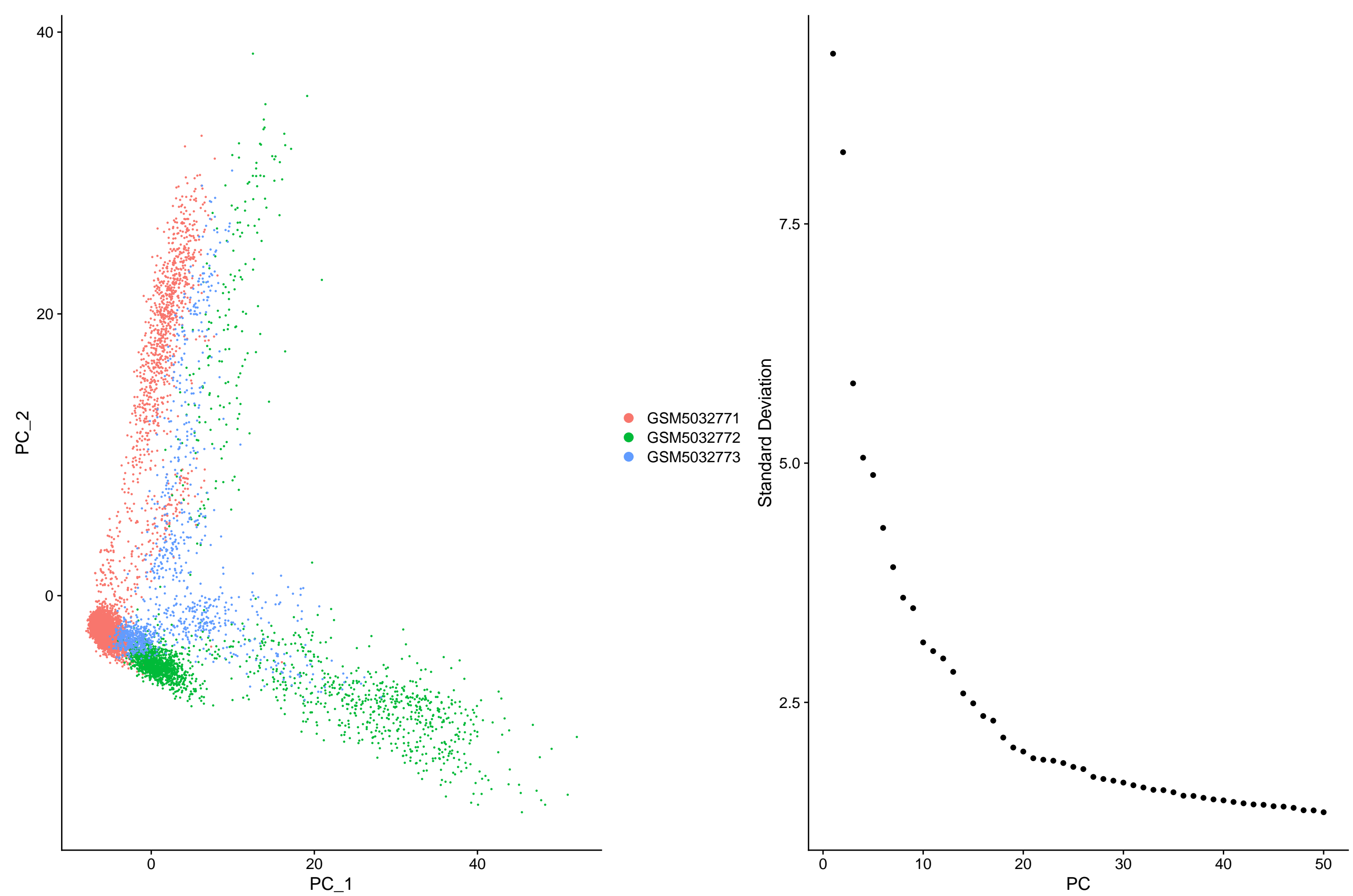

Supplement: Supplementary file 7 [file Data_Sheet_5.PDF]

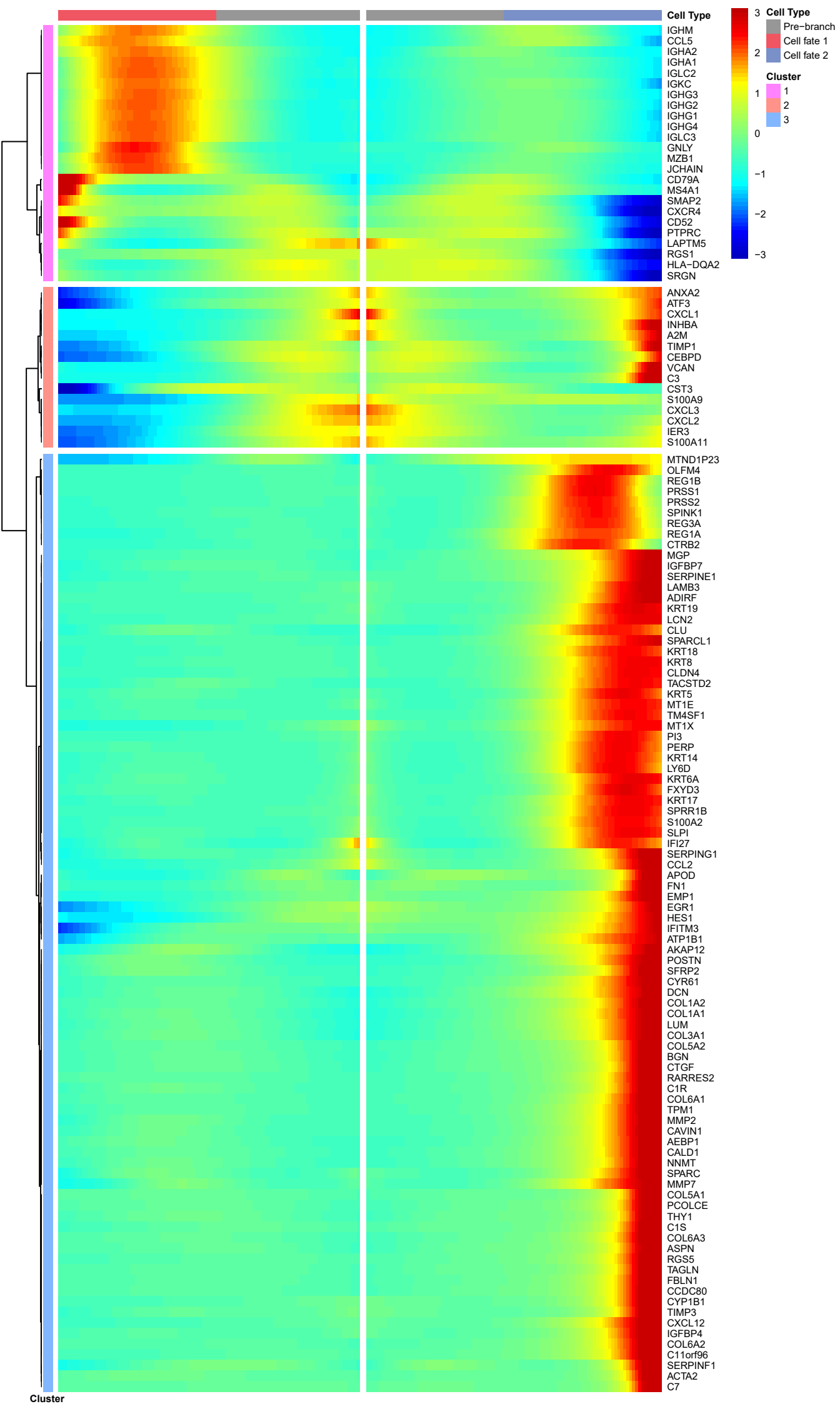

Supplement: Supplementary file 8 [file Data_Sheet_6.PDF]

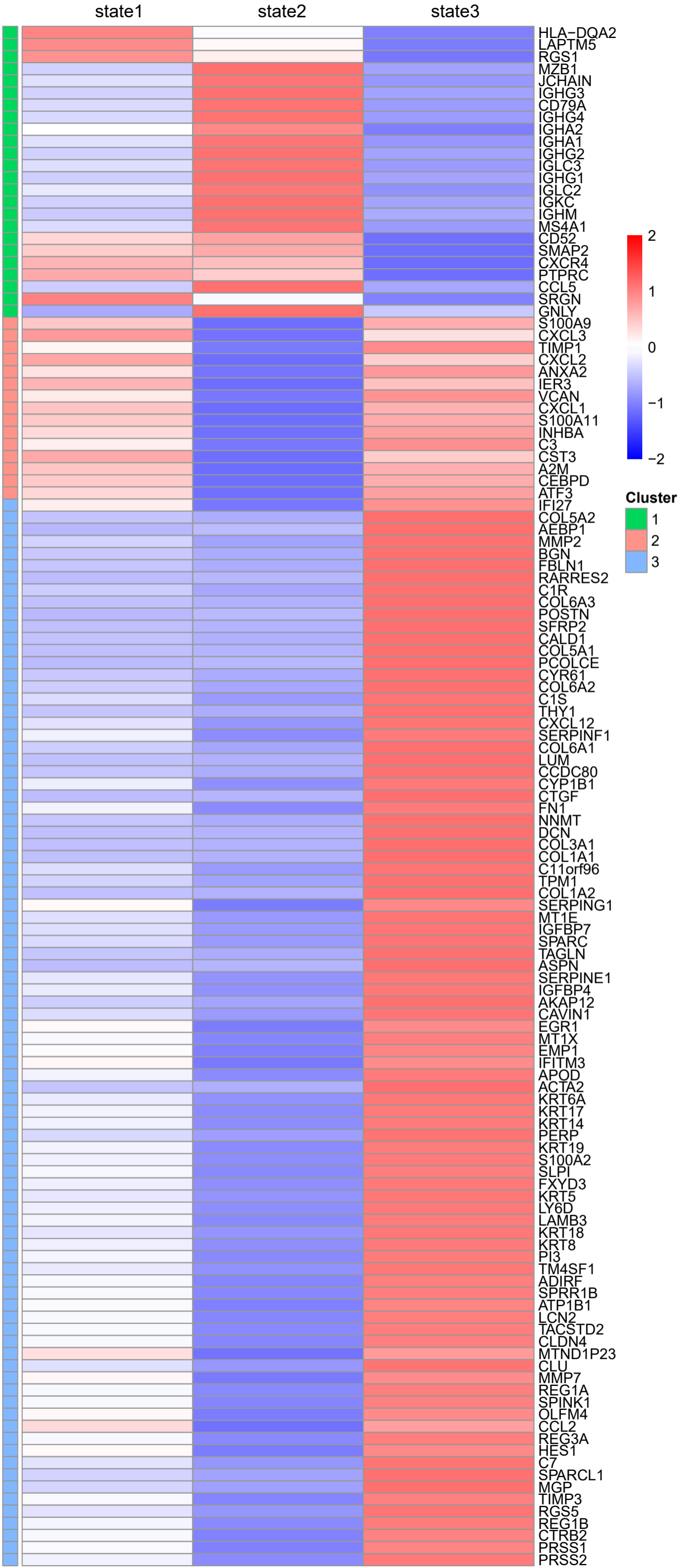

Supplement: Supplementary file 9 [file Data_Sheet_7.PDF]

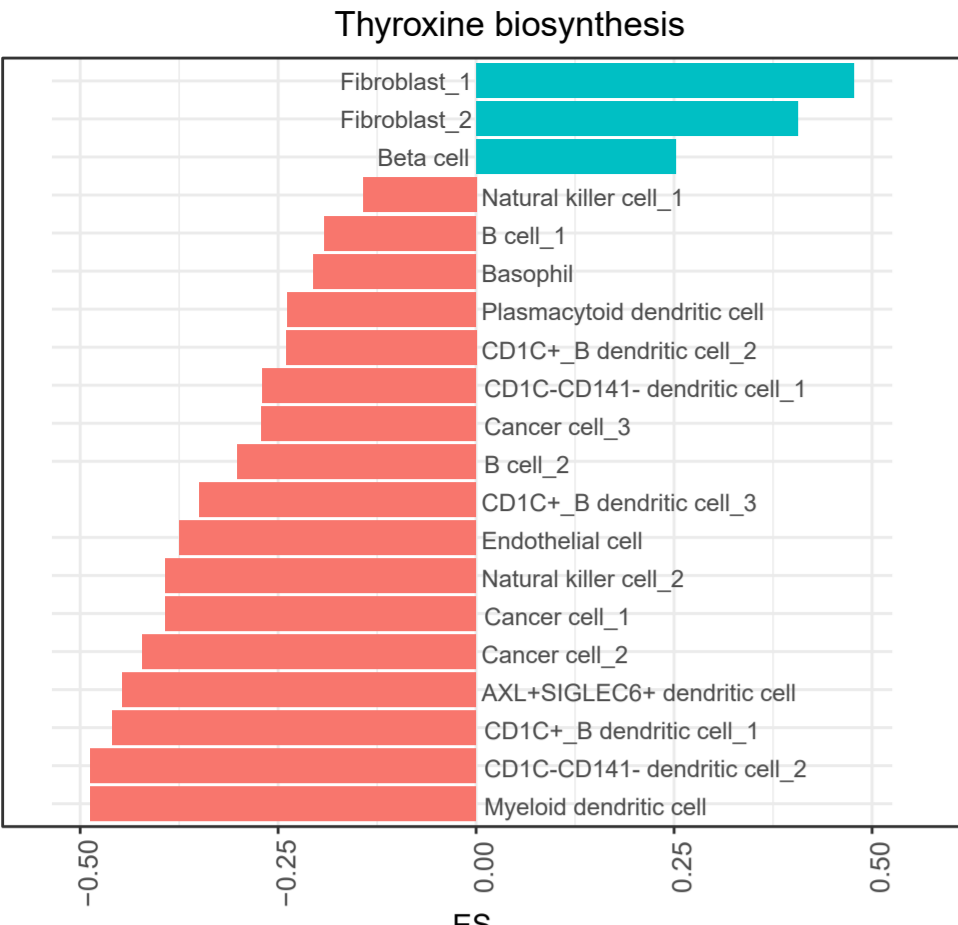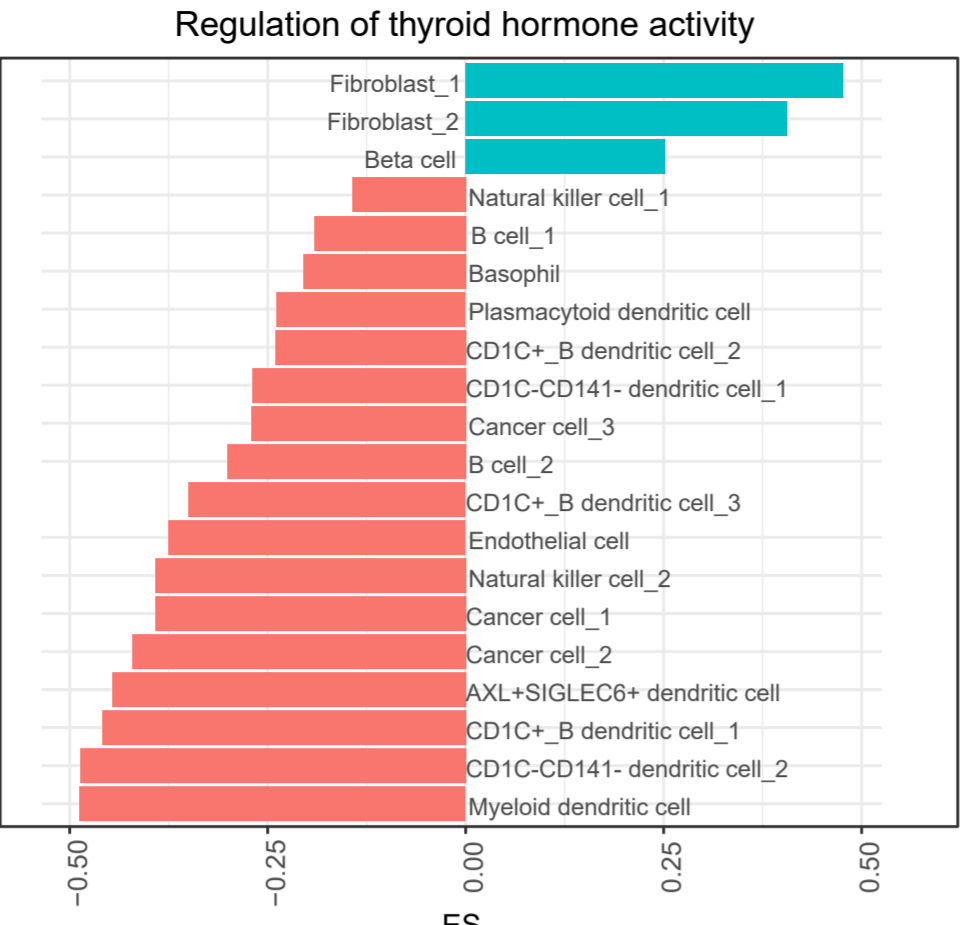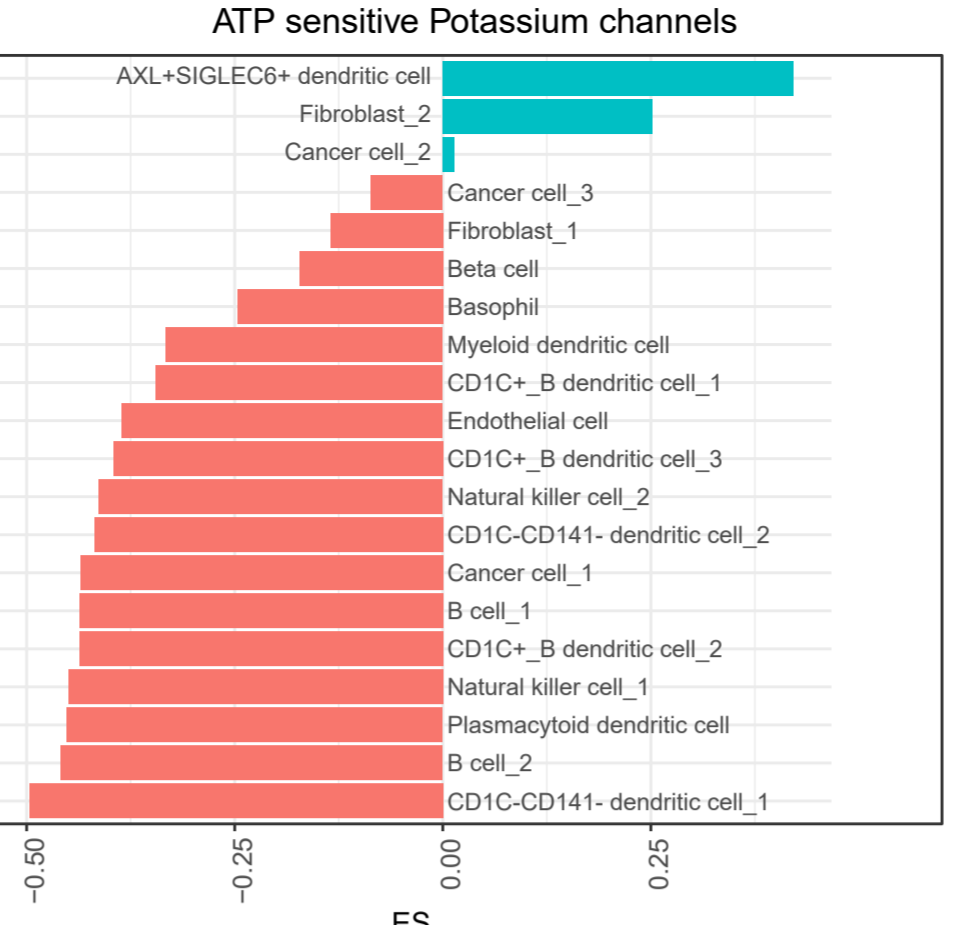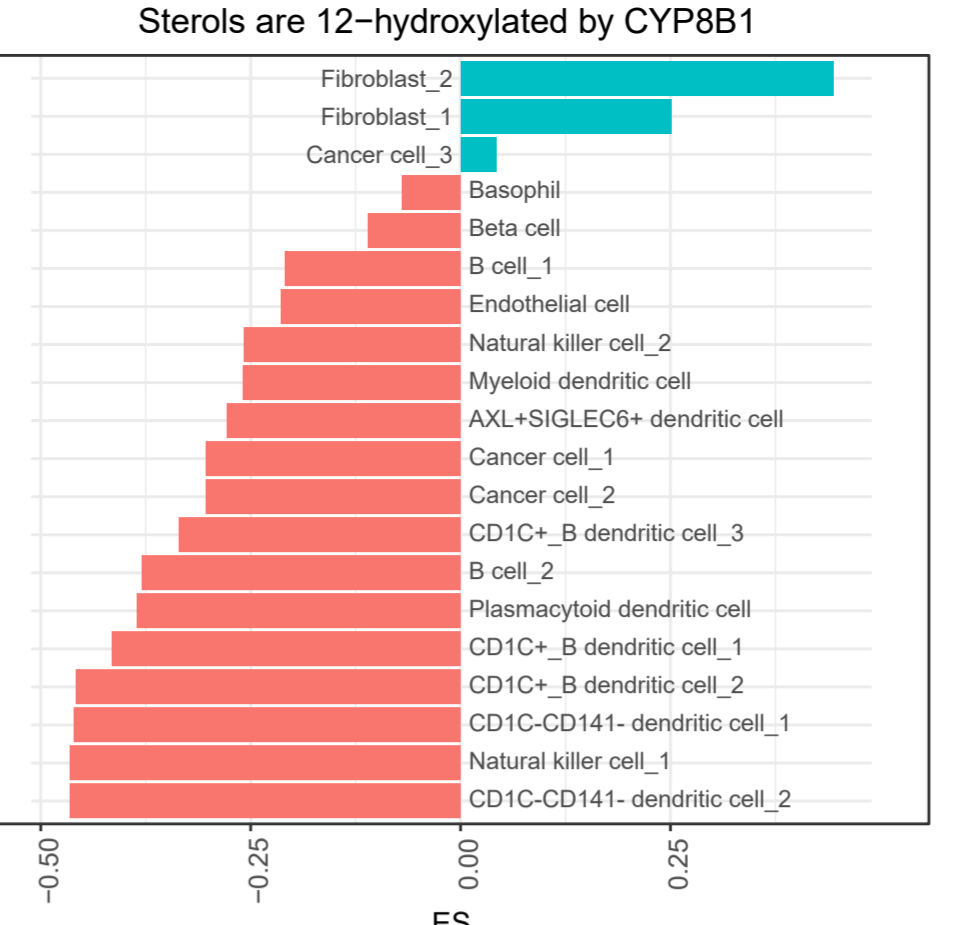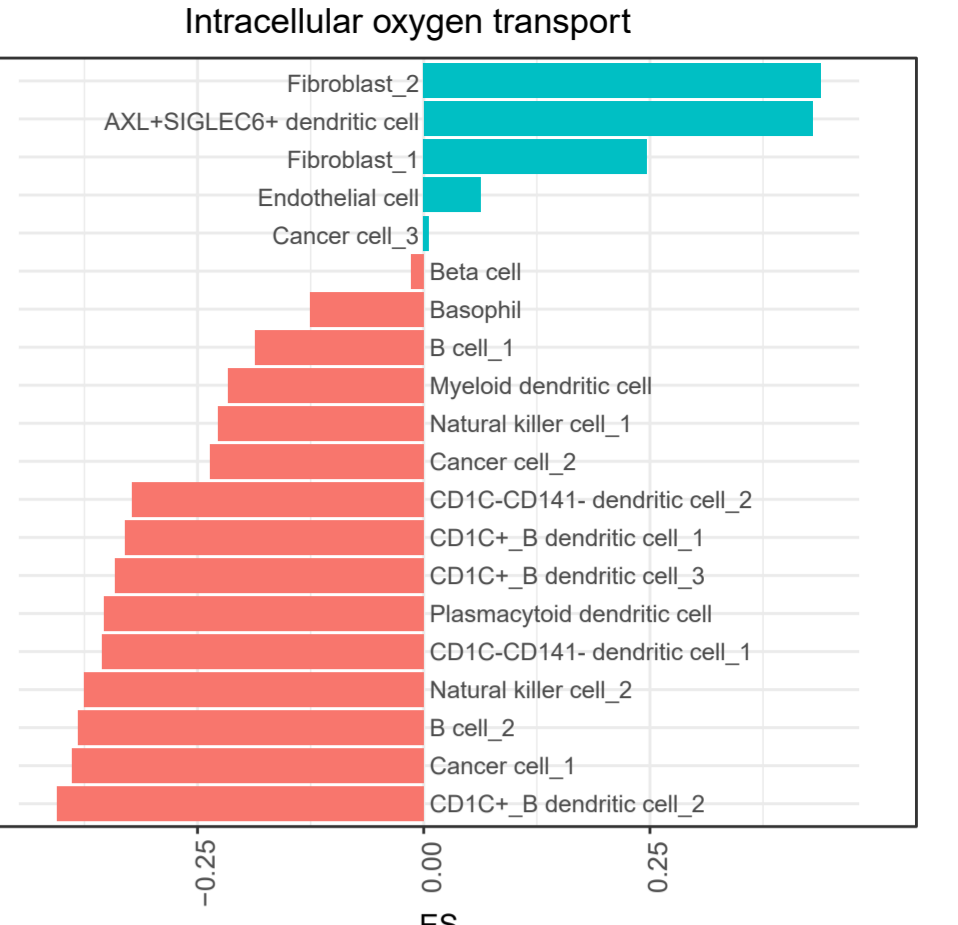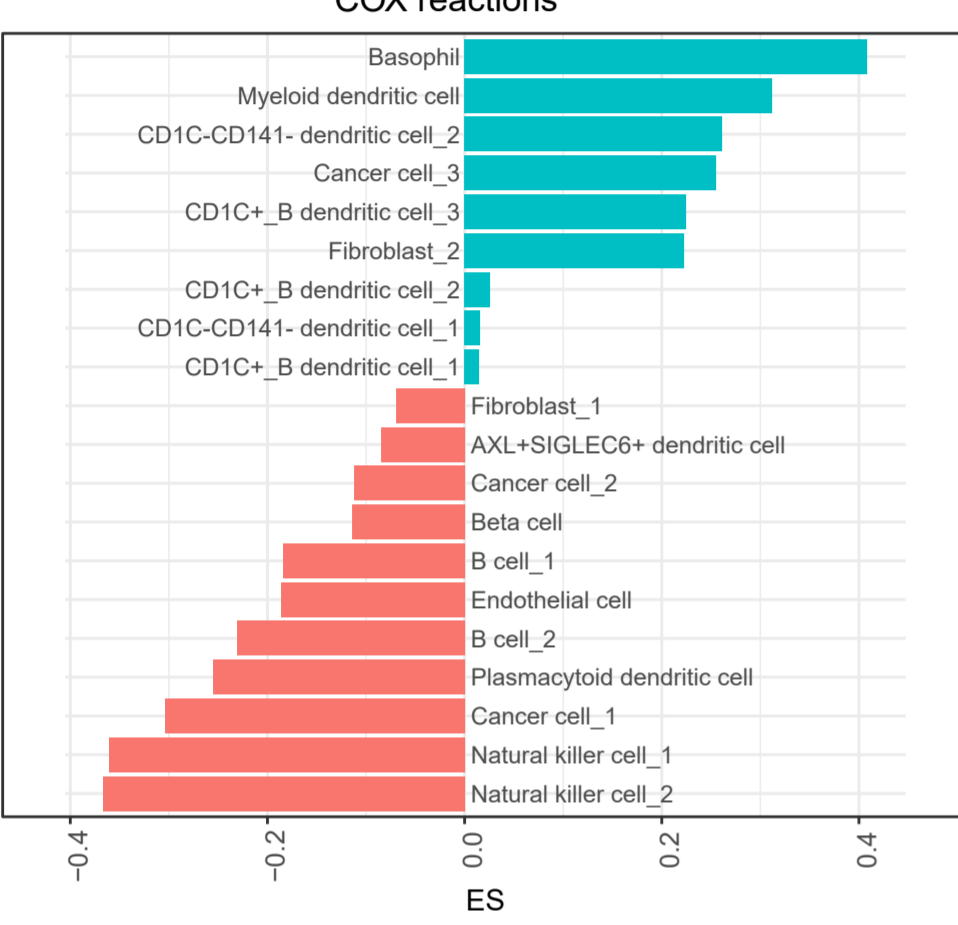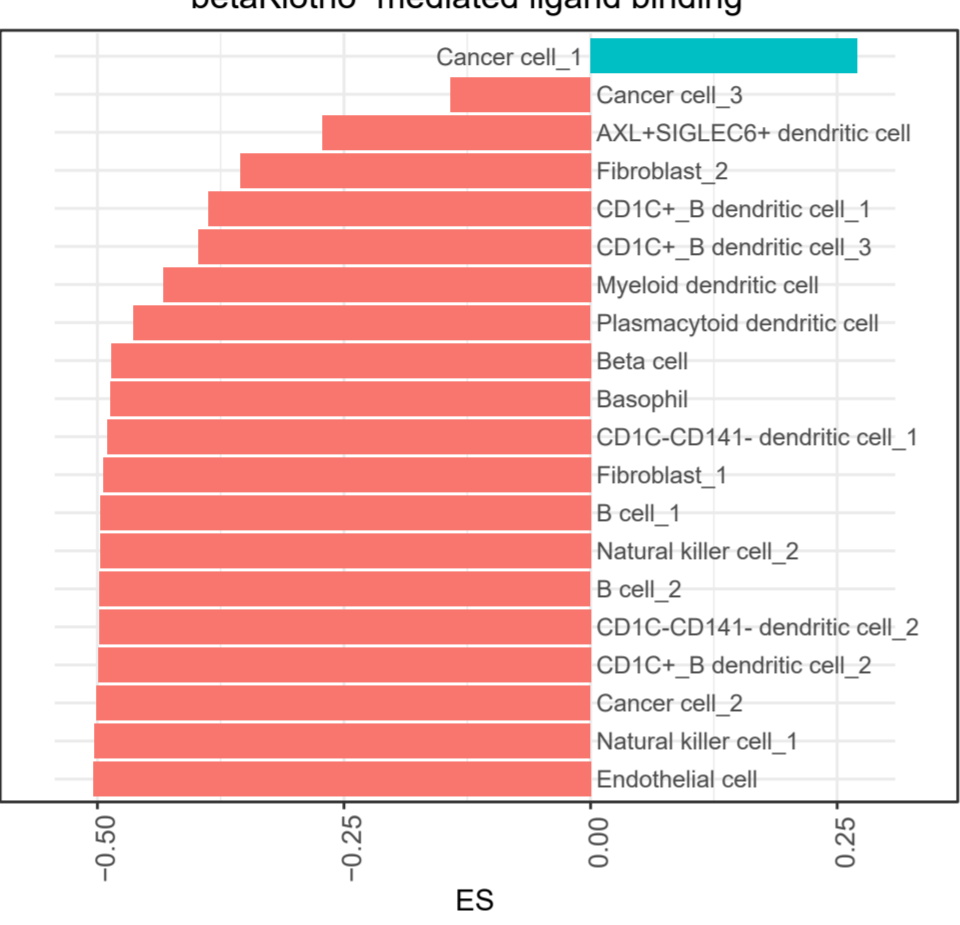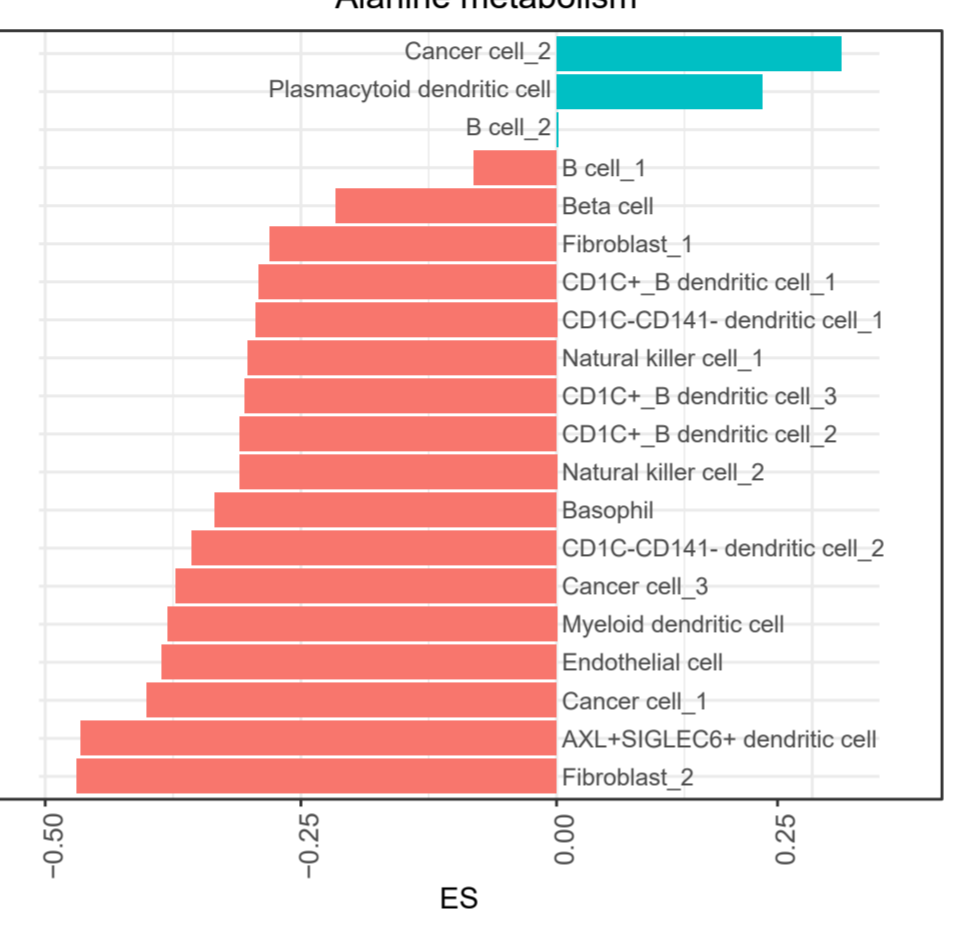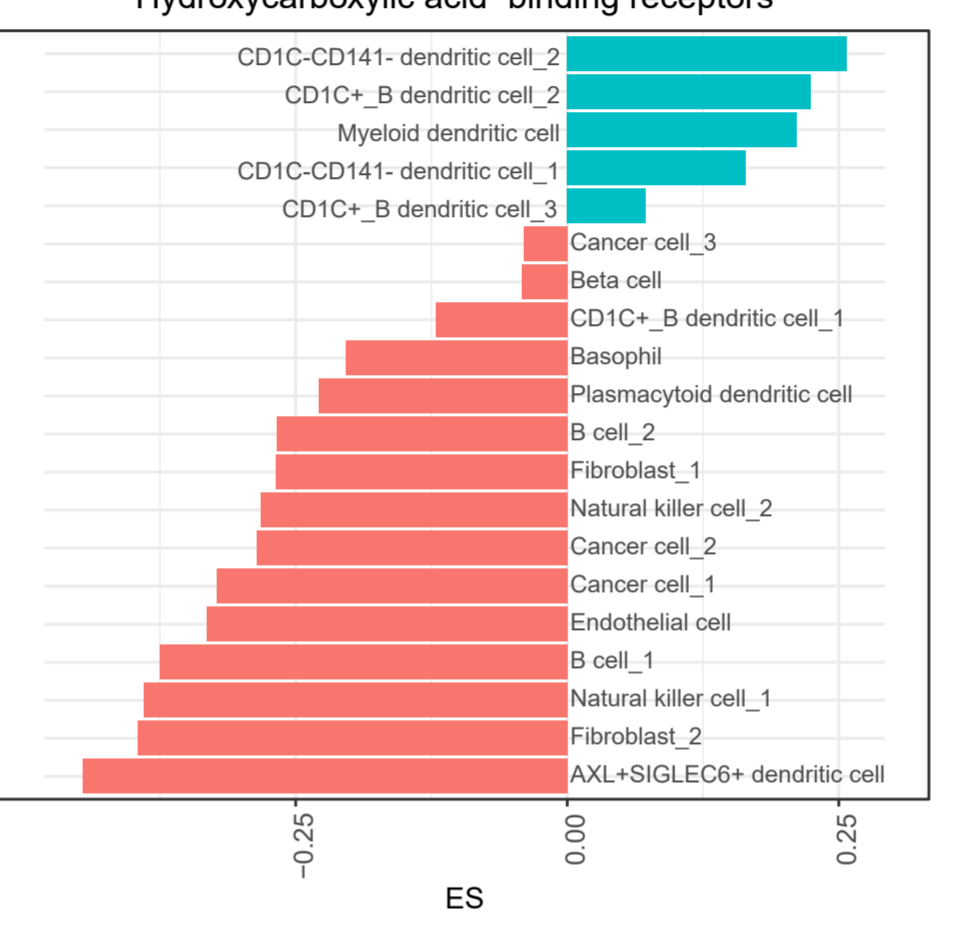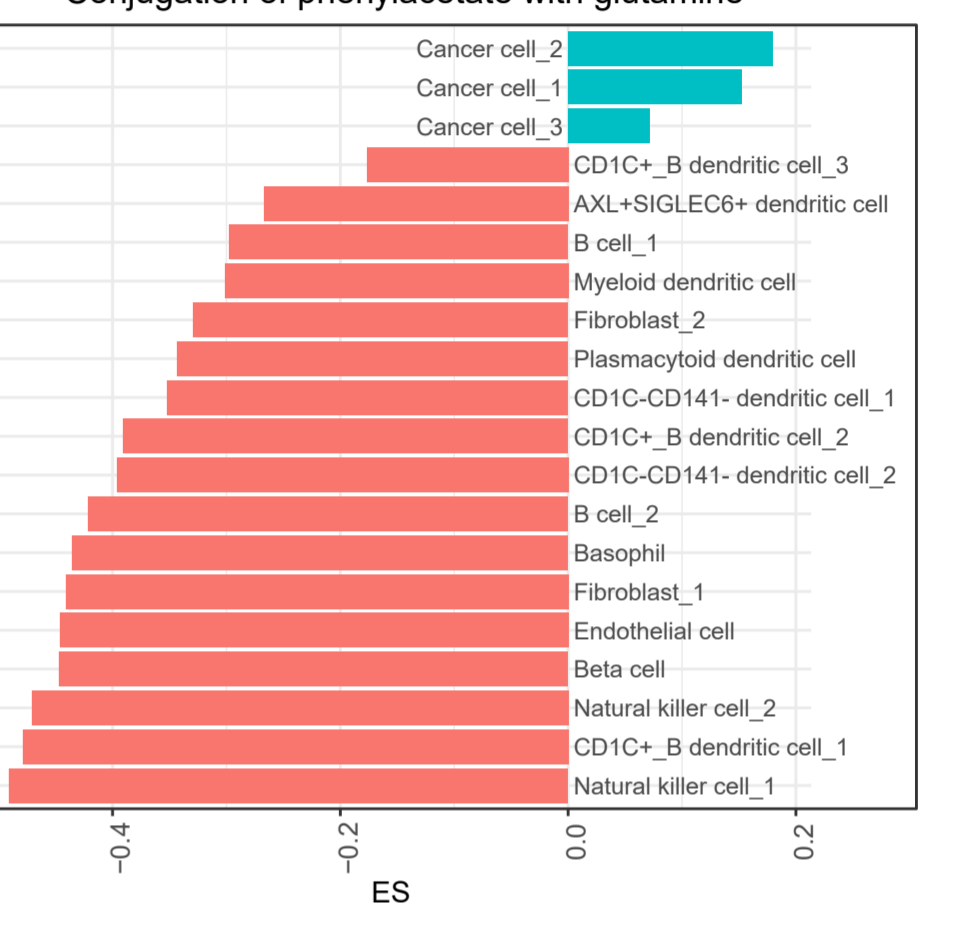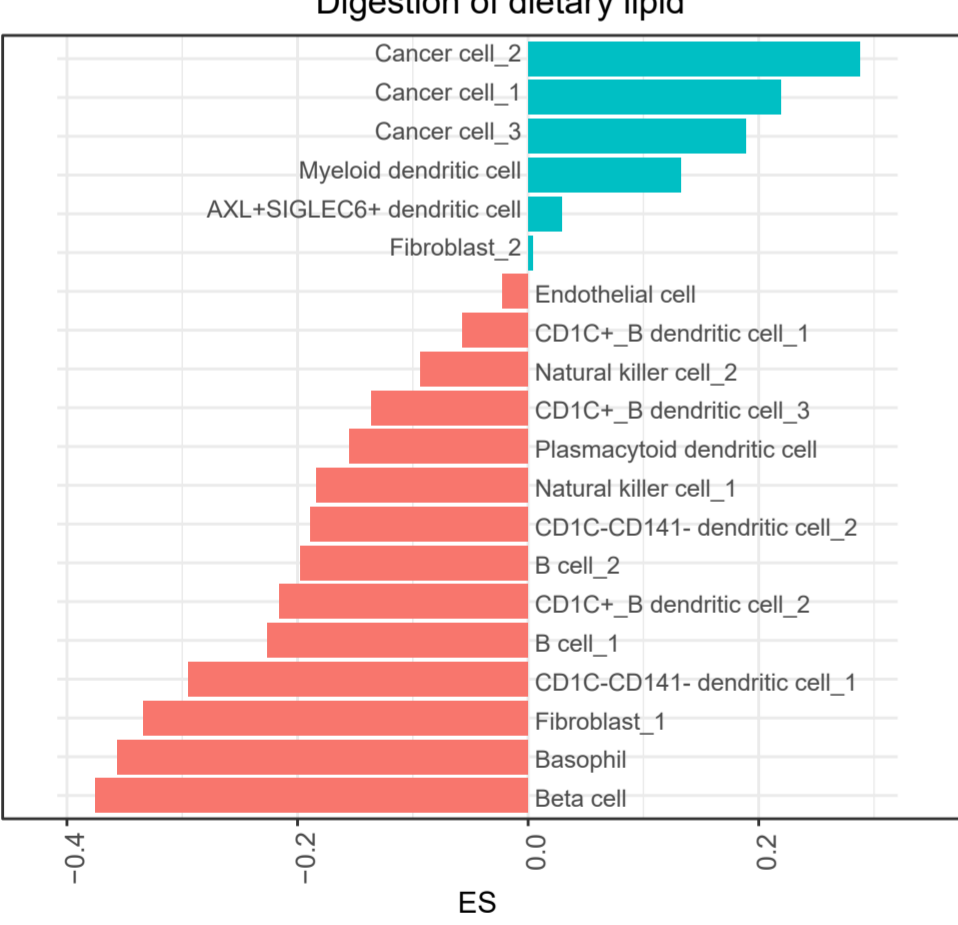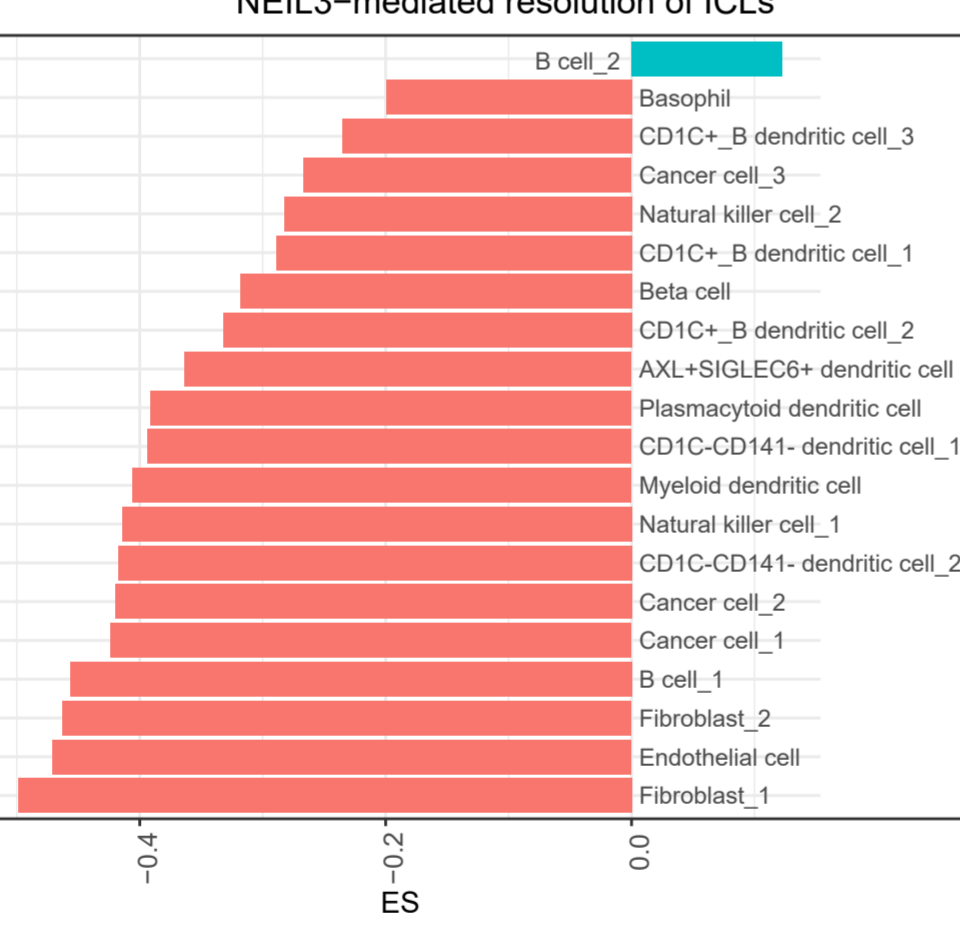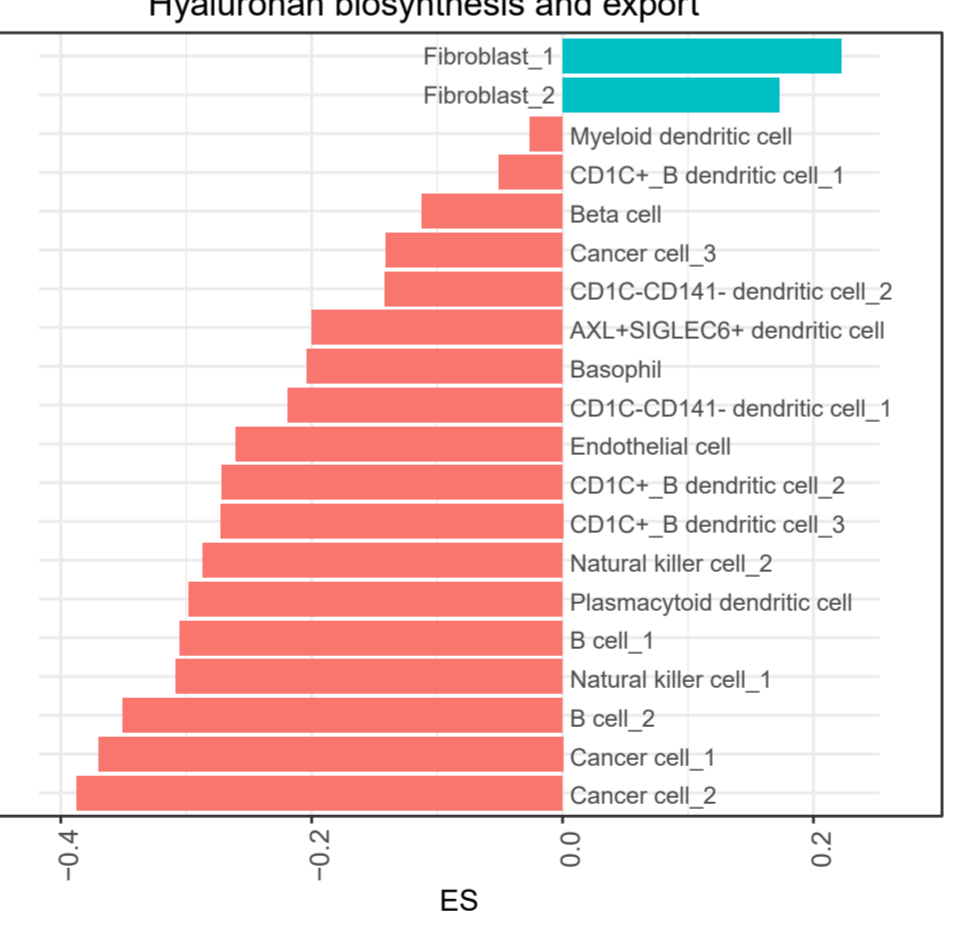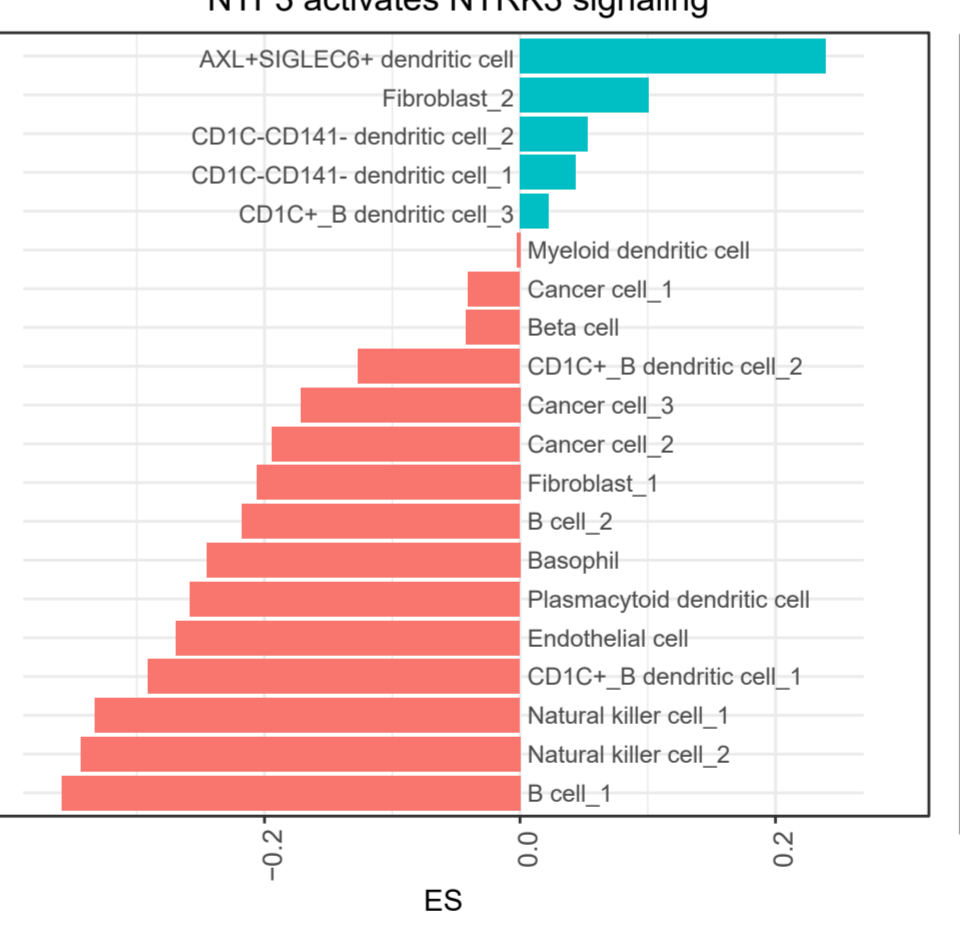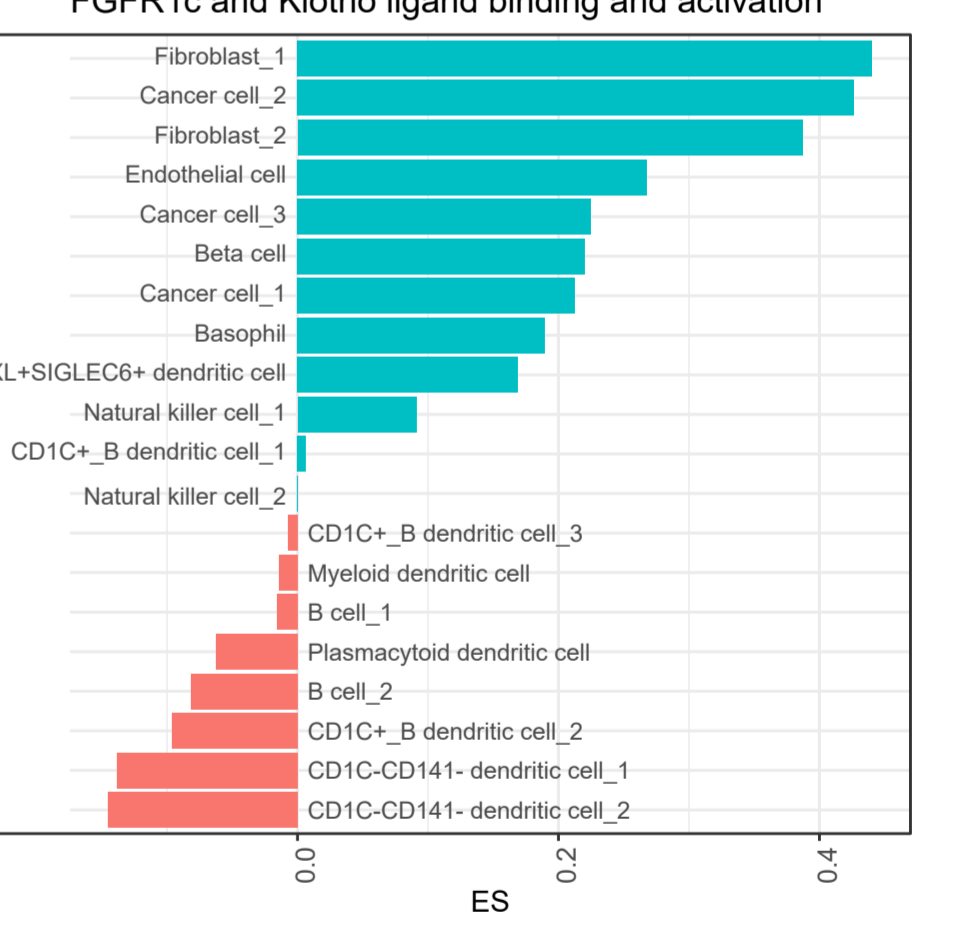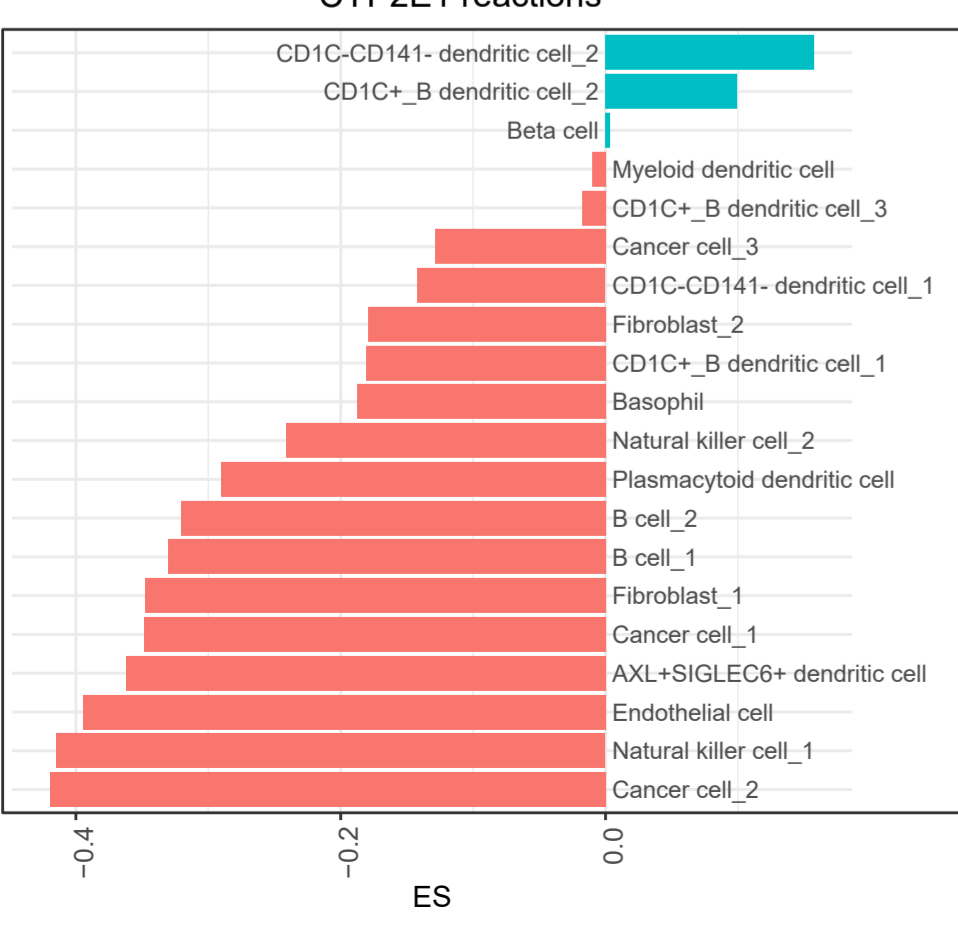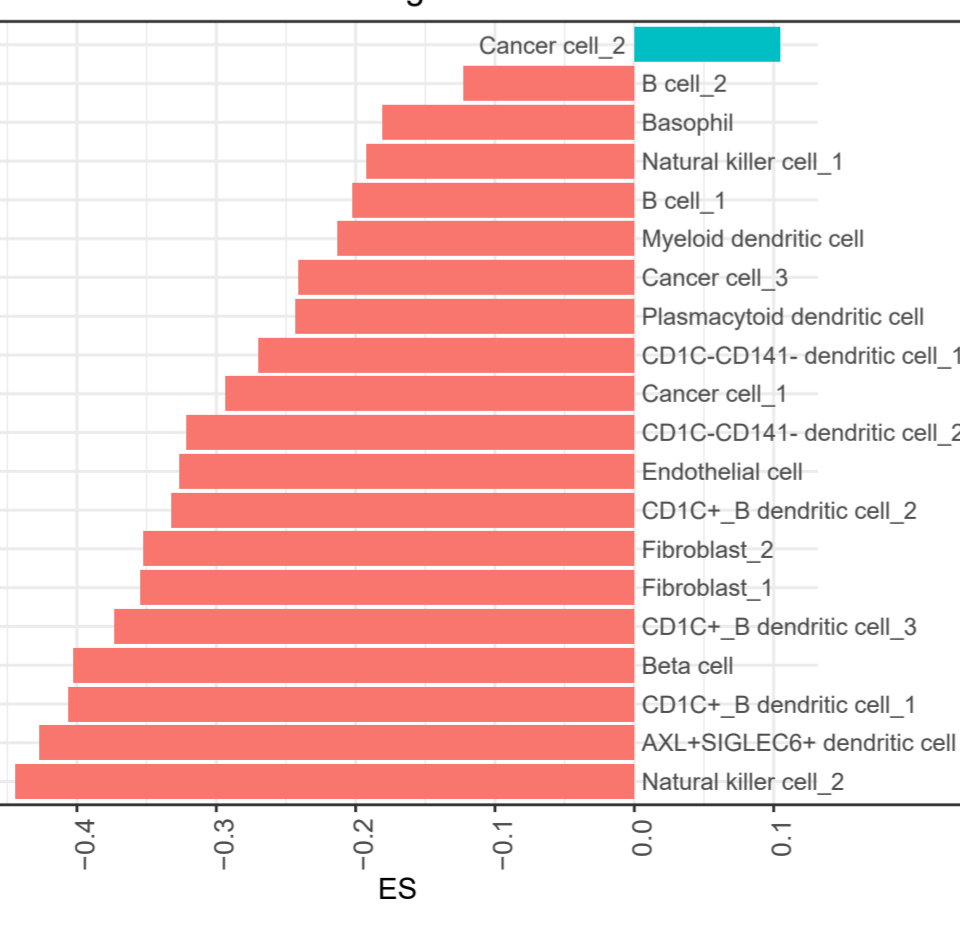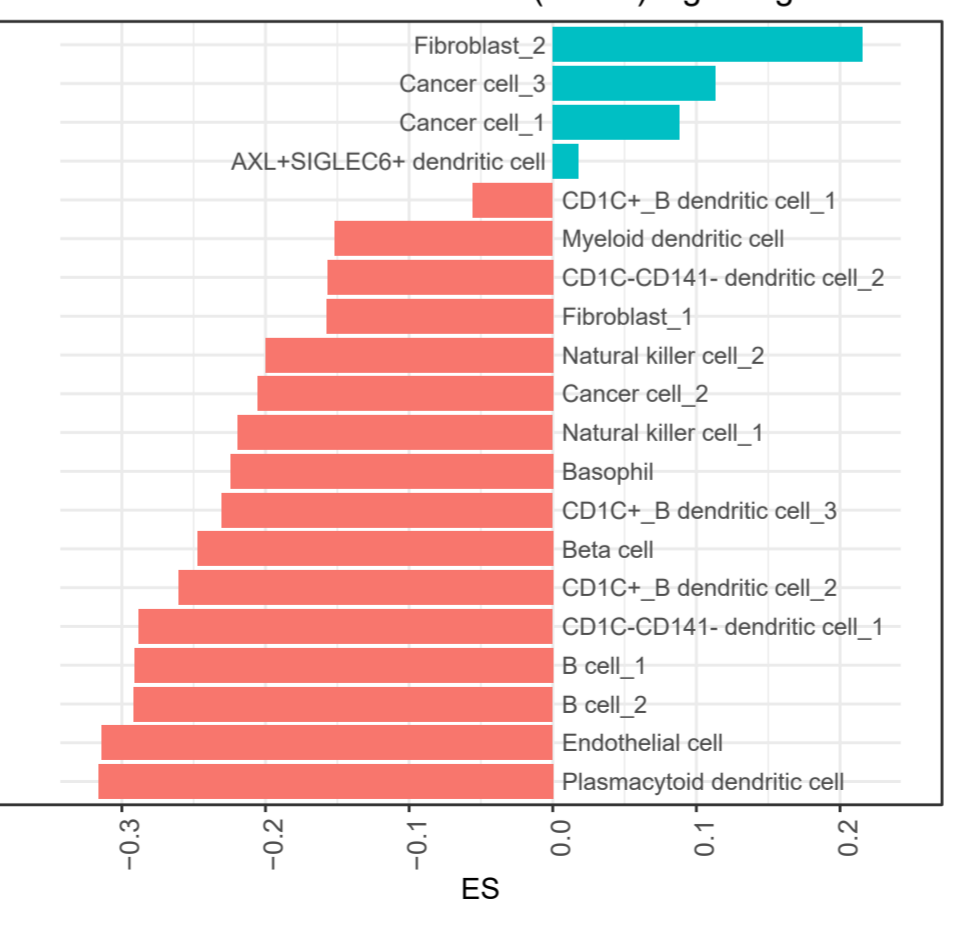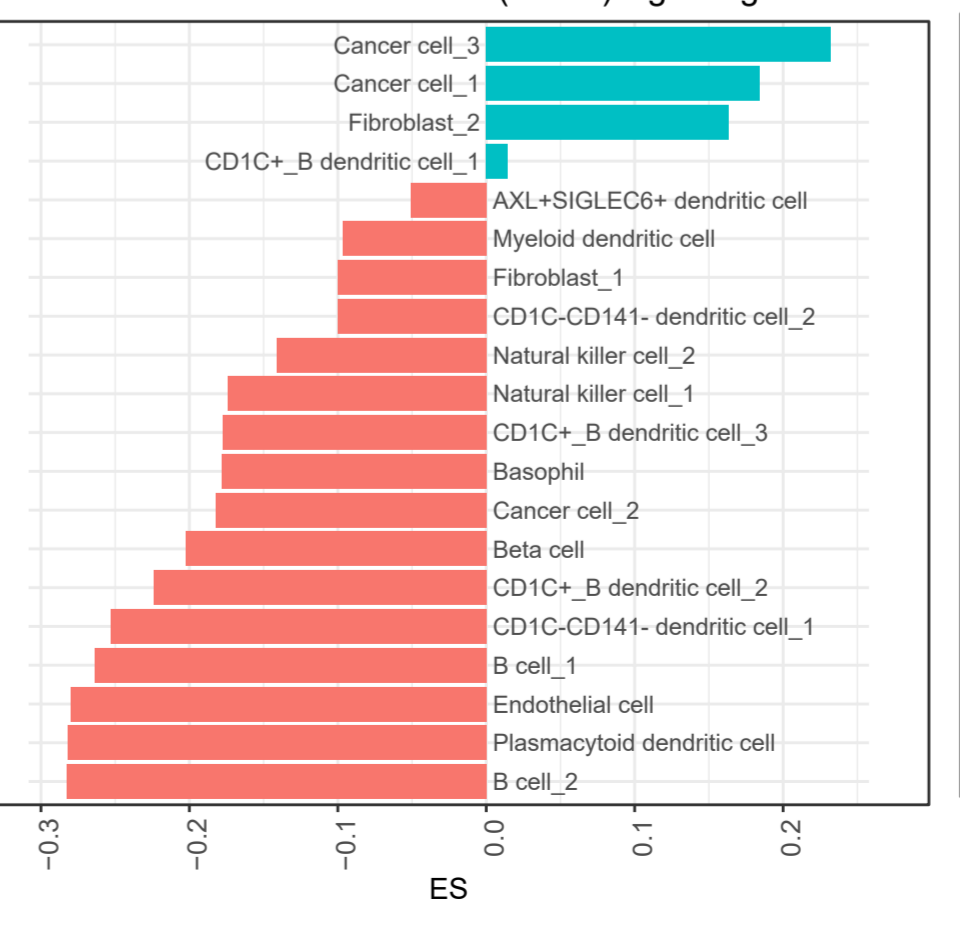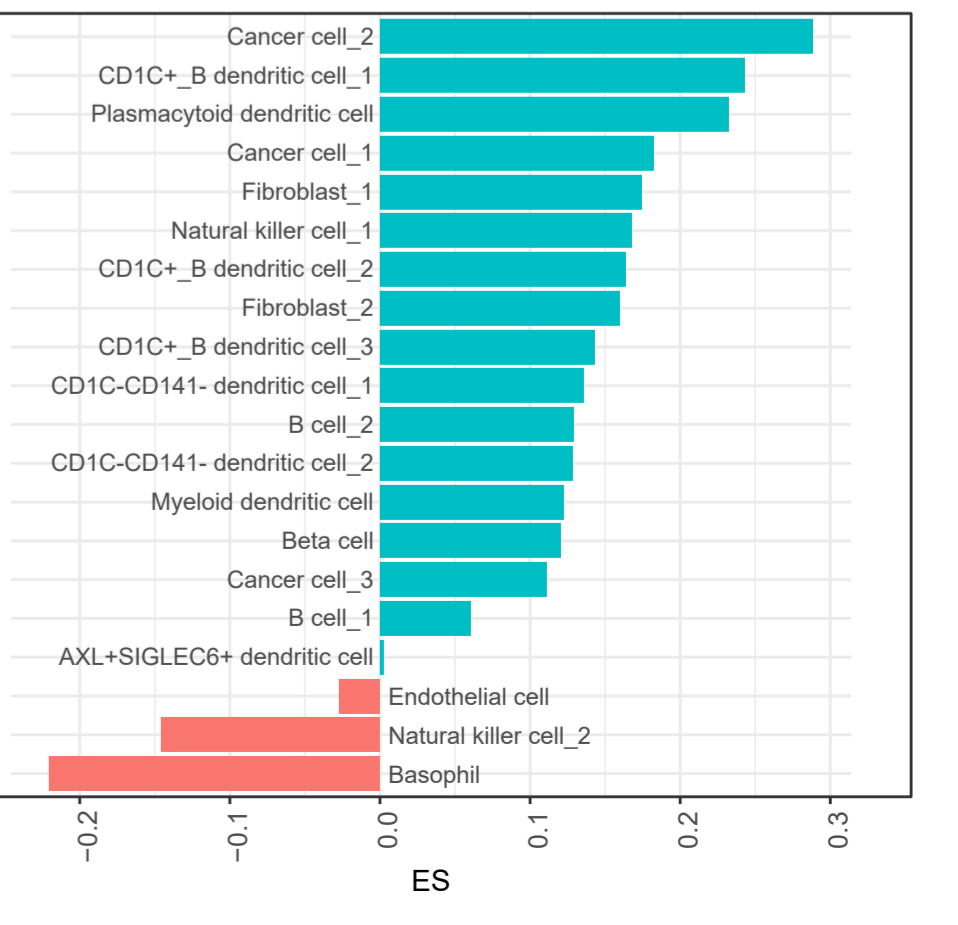

Supplement: Supplementary file 10 [file Data_Sheet_8.PDF]

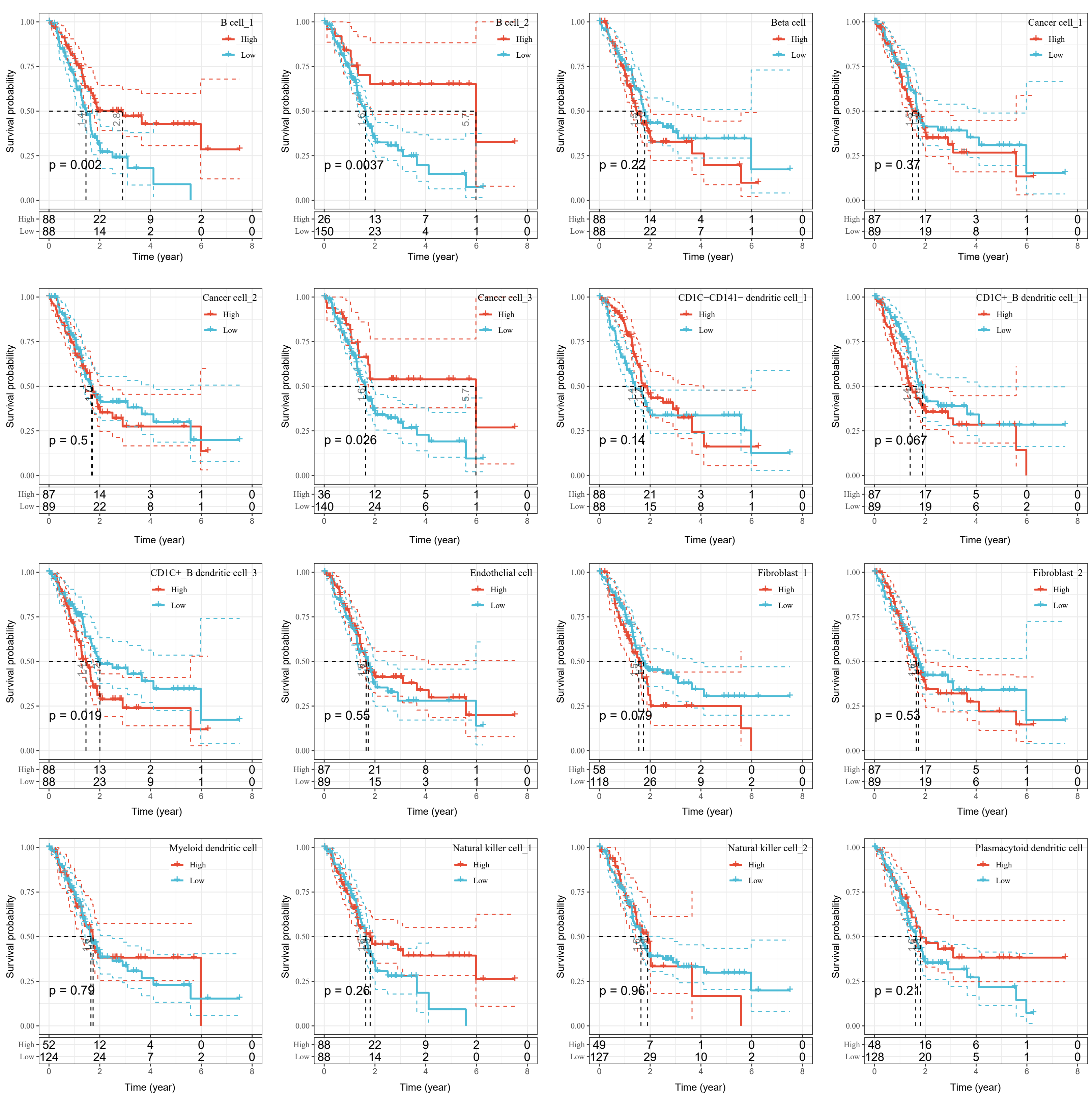

Supplement: Supplementary file 11 [file Data_Sheet_9.PDF]

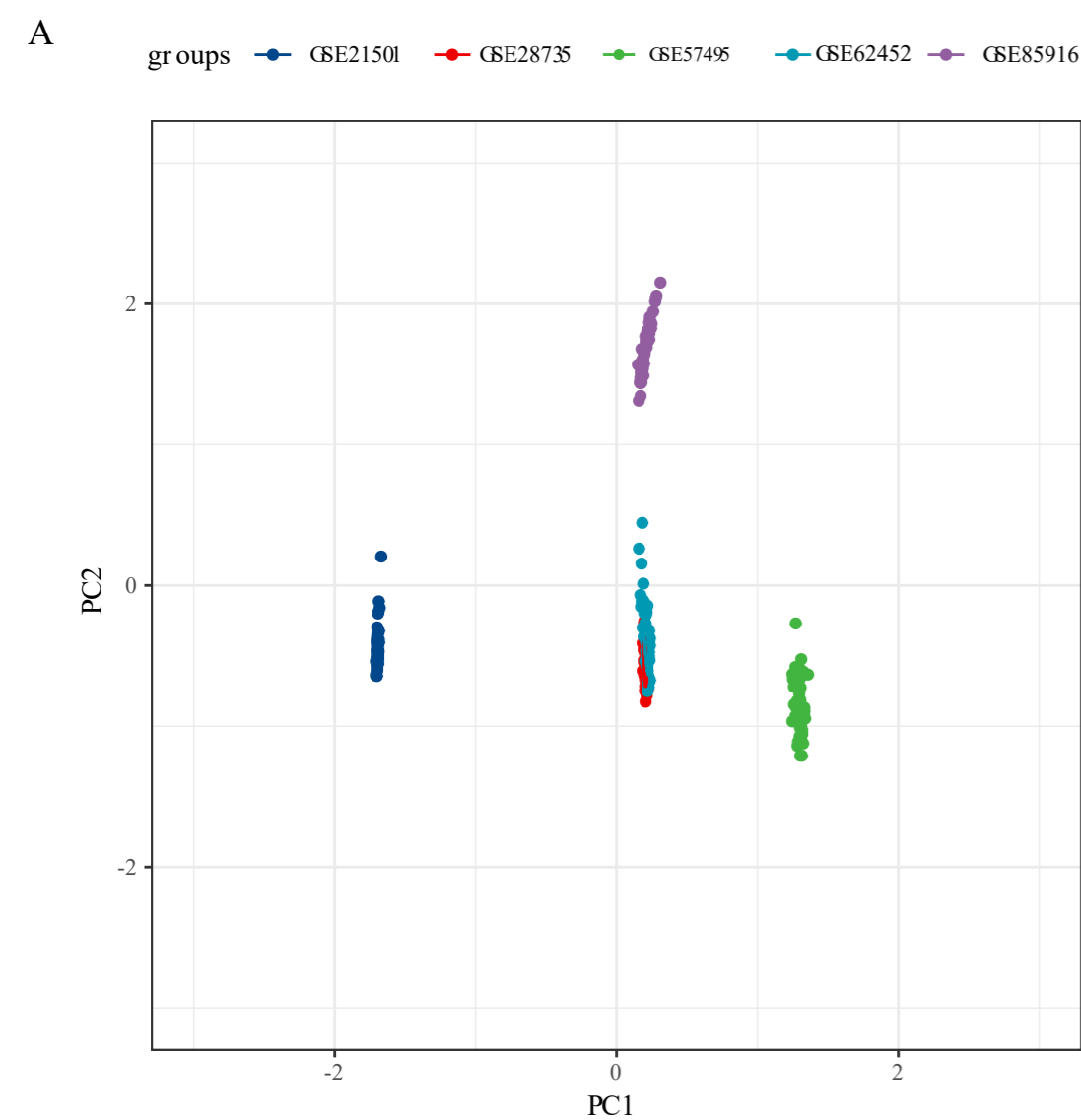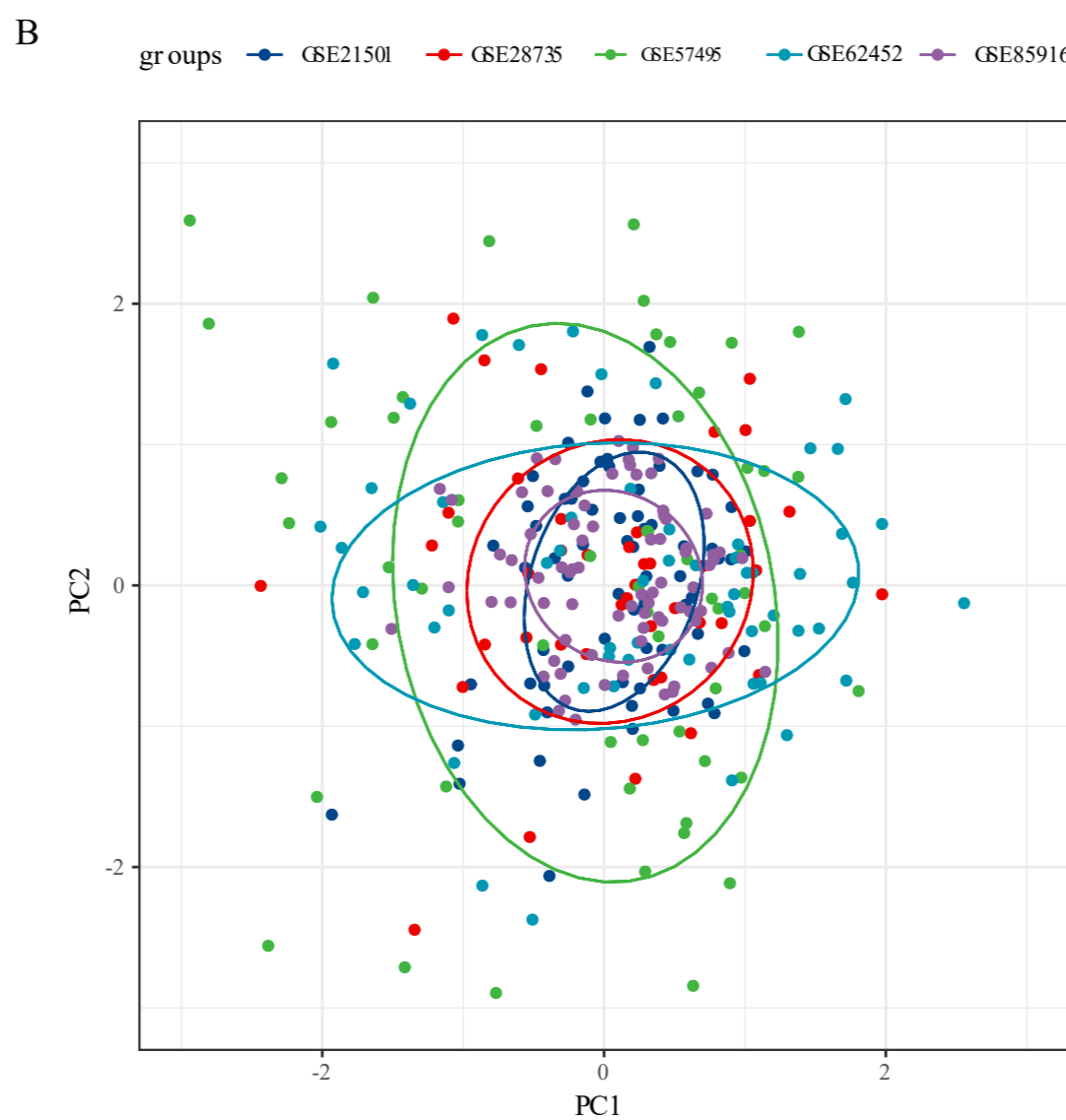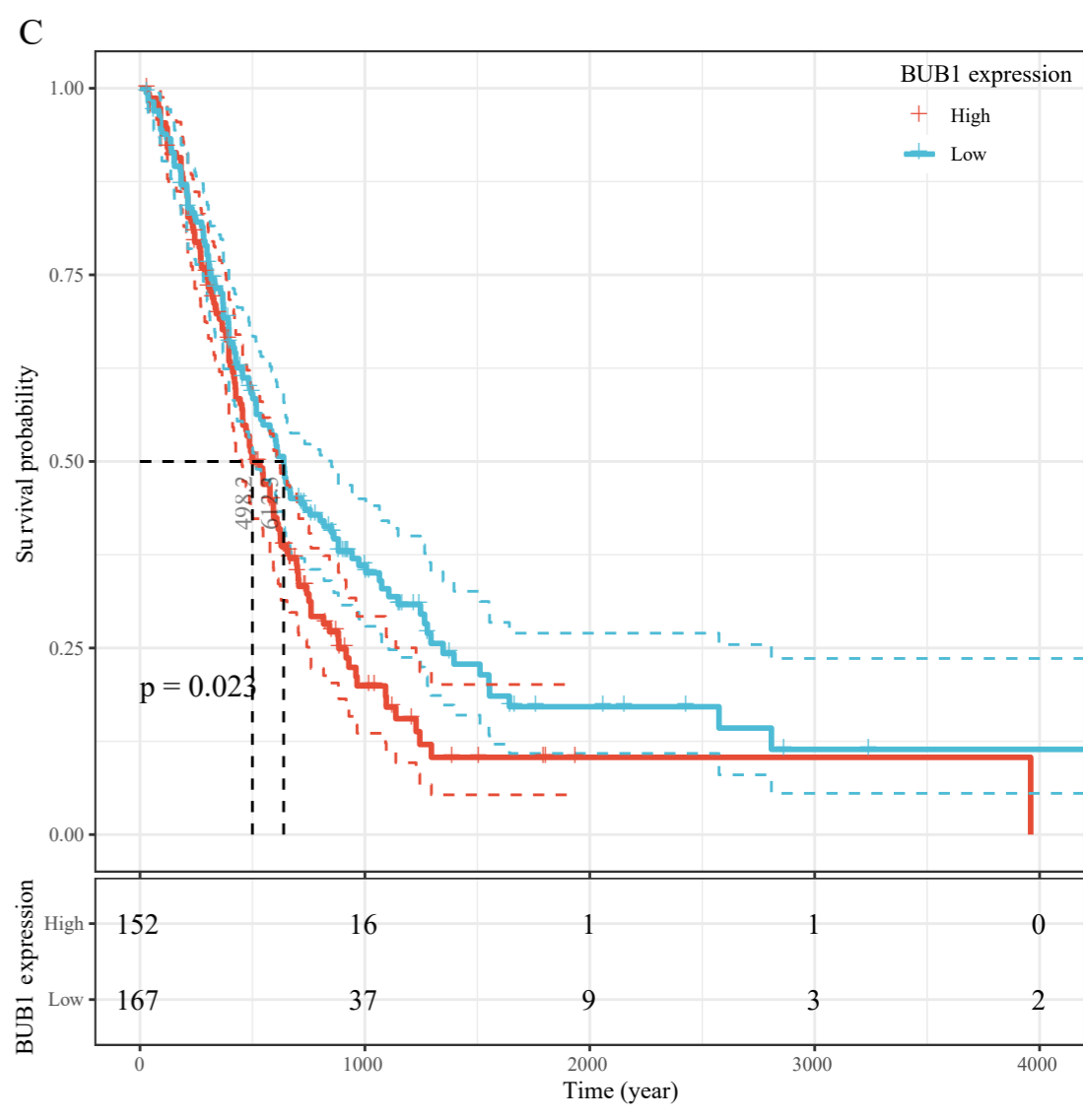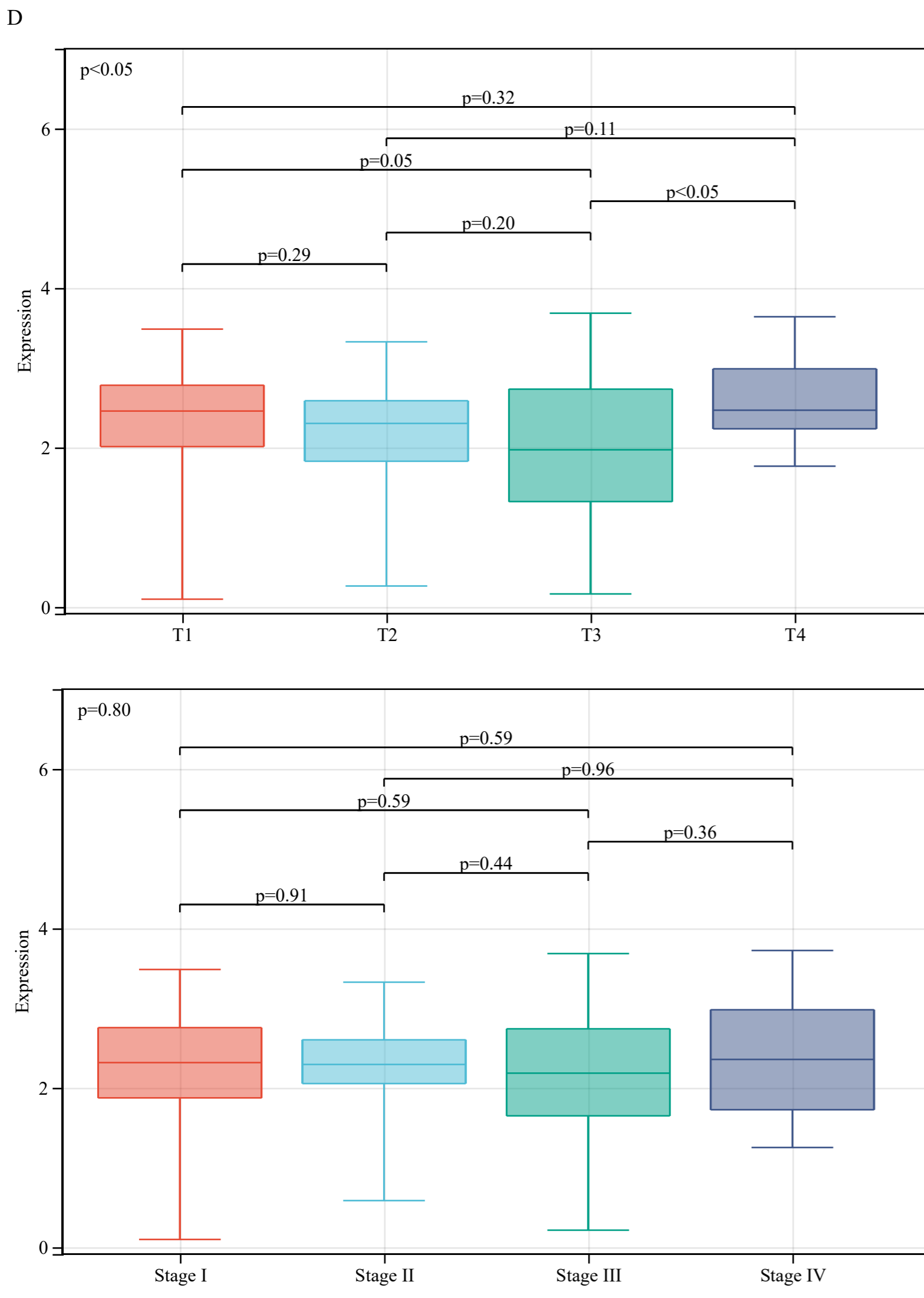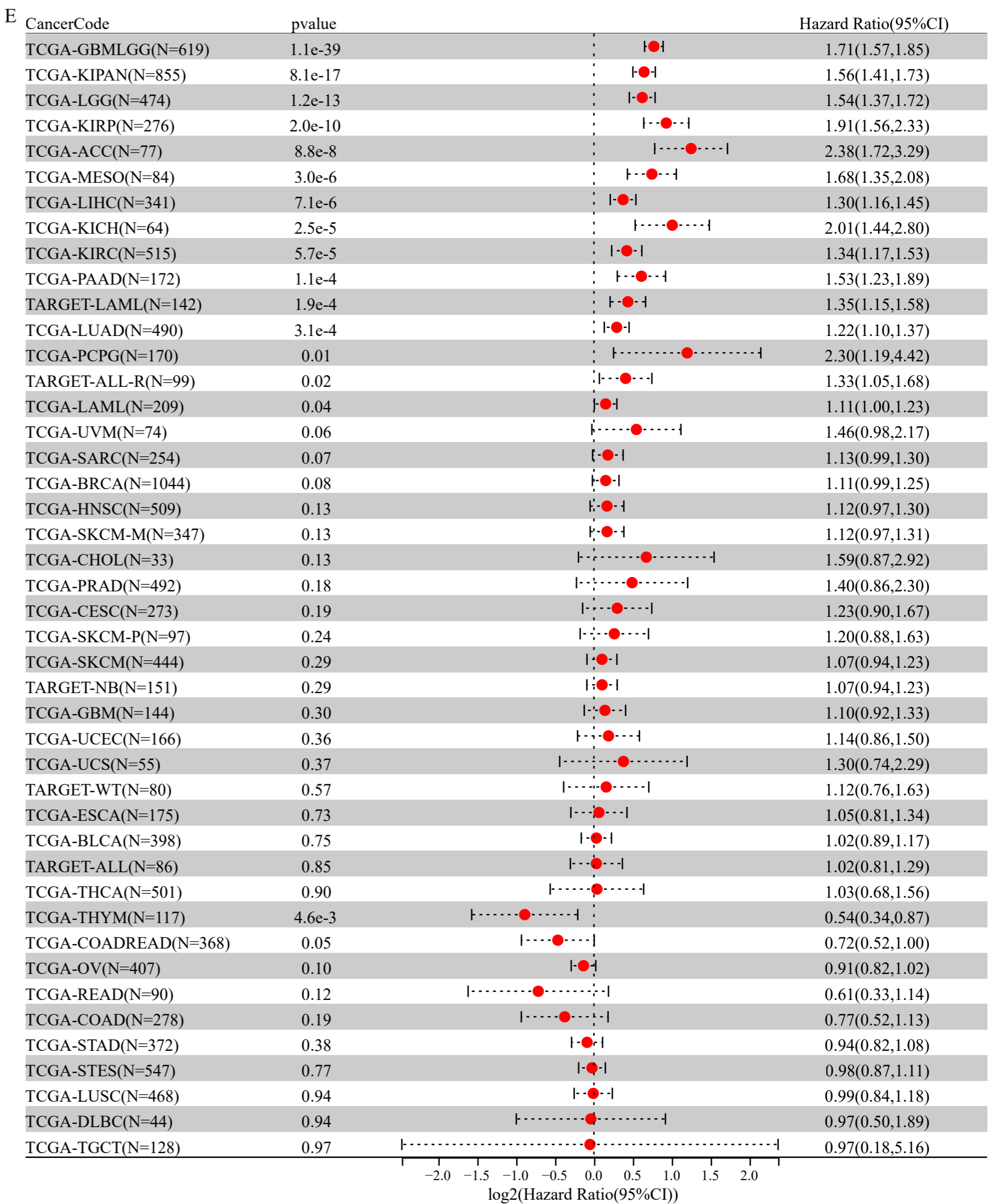

Supplement: Supplementary file 12 [file Data_Sheet_10.PDF]
