# Supplementary material for: BUB1 Is Identified as a Potential Therapeutic Target for Pancreatic Cancer Treatment
Source: Front Public Health. 2022 Jun 13;10:900853. doi: 10.3389/fpubh.2022.900853 (PMC9235519; doi:10.3389/fpubh.2022.900853)
Supplement: Supplementary file 13 [file Data_Sheet_11.PDF]

Description of supplementary materials

Supplementary Figure S1. Quality control of three single-cell samples (GSM5032771, GSM5032772 and GSM5032773) before preprocessing.

Supplementary Figure S2. Quality control of three single-cell samples (GSM5032771, GSM5032772 and GSM5032773) after preprocessing.

Supplementary Figure S3. The cell counts of each sample before and after preprocessing.

Supplementary Figure S4. Identification of highly variable genes. The top 20 variable genes were indicated.

Supplementary Figure S5. Confirmation of the optimal plane for dimensionality reduction.

Supplementary Figure S6. A heatmap of expression of 127 genes associated with cell development. Clustering was applied and three clusters were labeled in the left by different colors. Colors in the heatmap from red to blue indicates expression from high to low.

Supplementary Figure S7. The expression of 127 genes in three types of status. Colors from red to blue indicates expression from high to low.

Supplementary Figure S8. Enrichment score of 20 functional pathways in each subgroup.

Supplementary Figure S9. Kaplan-Meier survival plots of 16 subgroups grouped by high and low enrichment.

Supplementary Figure S10. Validation of BUB1 in other independent datasets. (A) The PCA plot of five datasets before removing batch effects. (B) The PCA plot of five datasets after removing batch effects. (C) Kaplan-Meier survival plot of BUB1 expression. (D) Differential distribution of BUB1 expression in clinical features. (E) Prognostic correlation forest map of BUB1 in Pan cancer.

Supplementary Table S1. The correlation of 14 gene pairs associated with prognosis.

Supplementary Table S2. The core code of the study.
